# NGS allele counts versus called genotypes for testing genetic association

**DOI:** 10.1016/j.csbj.2022.07.016

**Published:** 2022-07-11

**Authors:** Rosa González Silos, Christine Fischer, Justo Lorenzo Bermejo

**Affiliations:** a Institute of Medical Biometry, University of Heidelberg, 69120, Germany; b Institute of Human Genetics, University of Heidelberg, 69120, Germany

**Keywords:** Genotype calling, Next generation sequencing, Allele counts, Genetic association tests, Statistical power, LD, Linkage disequilibrium, MAF, Minor allele frequency, GATK, Genomic analysis toolkit, NGS, Next-generation sequencing, SD, Standard deviation, VCF, Variant call format, YRI, Yoruba in Ibadan, Nigeria

## Abstract

RNA sequence data are commonly summarized as read counts. By contrast, so far there is no alternative to genotype calling for investigating the relationship between genetic variants determined by next-generation sequencing (NGS) and a phenotype of interest. Here we propose and evaluate the direct analysis of allele counts for genetic association tests. Specifically, we assess the potential advantage of the ratio of alternative allele counts to the total number of reads aligned at a specific position of the genome (coverage) over called genotypes. We simulated association studies based on NGS data from HapMap individuals. Genotype quality scores and allele counts were simulated using NGS data from the Personal Genome Project. Real data from the 1000 Genomes Project was also used to compare the two competing approaches. The average proportions of probability values lower or equal to 0.05 amounted to 0.0496 for called genotypes and 0.0485 for the ratio of alternative allele counts to coverage in the null scenario, and to 0.69 for called genotypes and 0.75 for the ratio of alternative allele counts to coverage in the alternative scenario (9% power increase). The advantage in statistical power of the novel approach increased with decreasing coverage, with decreasing genotype quality and with decreasing allele frequency – 124% power increase for variants with a minor allele frequency lower than 0.05. We provide computer code in R to implement the novel approach, which does not preclude the use of complementary data quality filters before or after identification of the most promising association signals.

**Author summary:**

Genetic association tests usually rely on called genotypes. We postulate here that the direct analysis of allele counts from sequence data improves the quality of statistical inference. To evaluate this hypothesis, we investigate simulated and real data using distinct statistical approaches. We demonstrate that association tests based on allele counts rather than called genotypes achieve higher statistical power with controlled type I error rates.

## 1 Introduction

Technical advances in next-generation sequencing (NGS) have already translated into large data collections and the need for efficient techniques to analyze them. Called genotypes are typically used to investigate the relationship between genetic variants and a phenotype of interest [1]. Genotypes are usually called using probabilistic methods, which rely on genotype quality scores and allele counts computed after read alignment and base calling [2]. The development of genotype-calling algorithms is an active research area [3], [4], [5], [6], [7], [8], [9]. Here we explore an alternative approach: direct use of the number of reference and alternative reads aligned at a specific position of the genome—allele counts, also referred to as allelic depths—instead of called genotypes [10], [11]. More precisely, we assess the potential advantage of the ratio of alternative allele counts to the total number of reads aligned at a specific position of the genome (coverage) over called genotypes.

## 2 Simulated datasets

**Fig. 1:**
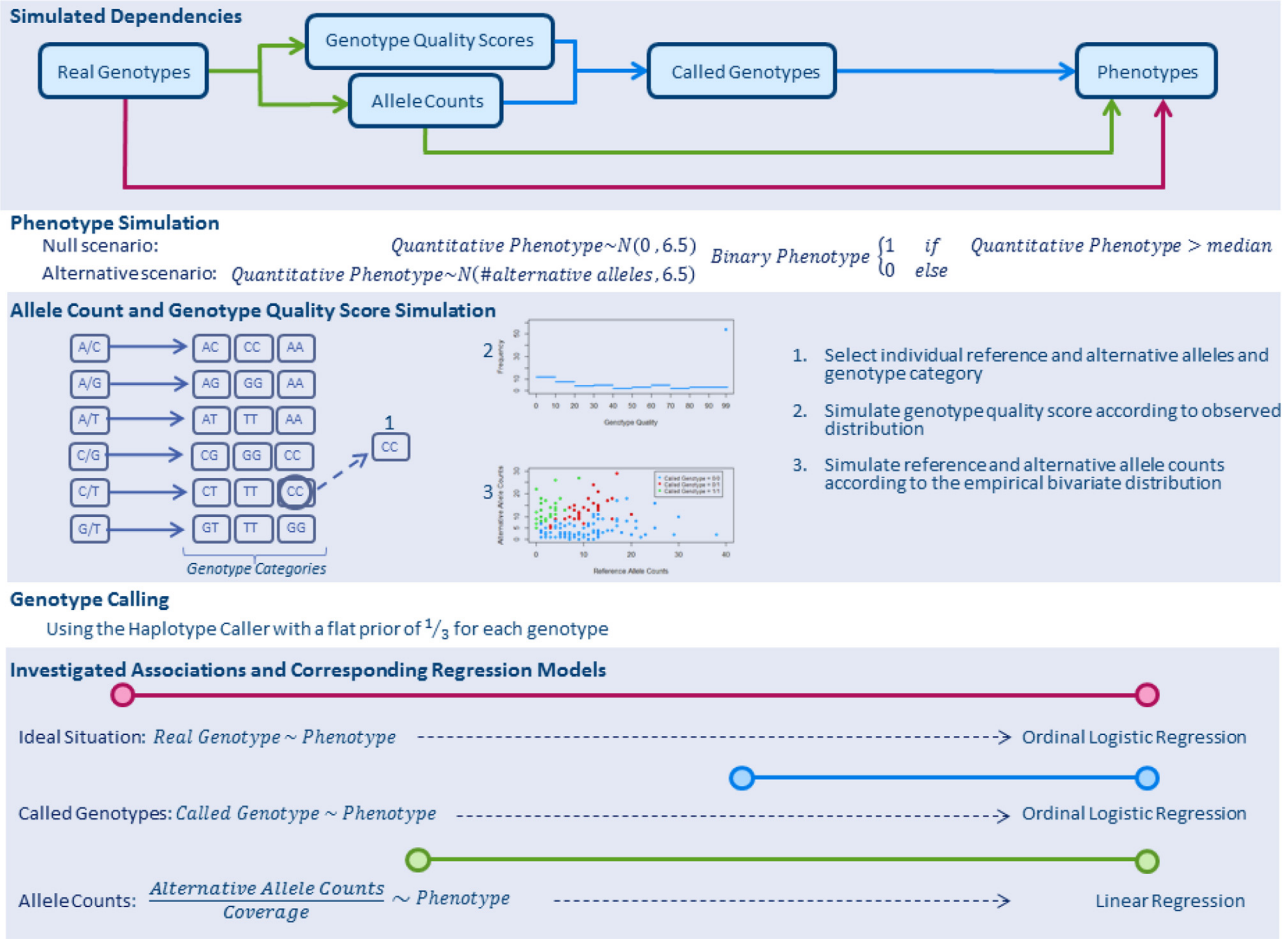
Overview of the performed simulations.

We simulated association studies relying on NGS data from 1417 HapMap individuals [12]. Fig. 1 depicts the implemented simulation steps for each of 27,139 genetic variants on chromosome 20. Quantitative phenotypes were assigned according to a null and an alternative scenario for each variant. In the null scenario, phenotypes were sampled from a normal distribution with mean 0 and standard deviation (SD) 6.5 independently of individual genotypes. In order to achieve approximately 80 % statistical power, quantitative phenotypes were sampled from a normal distribution with mean equal to the number of individual alternative alleles (0, 1, or 2) and SD equal to 6.5 in the alternative scenario. Binary phenotypes were derived from quantitative phenotypes according to median split.

We simulated genotype quality scores and allele counts for each individual genotype based on NGS data from the Personal Genome Project [13]. First, individual genotypes were grouped into 18 different categories according to the reference allele, the alternative allele and the combined genotype (Fig. 1). Next, genotype quality scores were randomly sampled from the observed (genotype category-specific) distribution of genotype quality scores. Based on the selected genotype quality score, allele counts were randomly sampled from the observed bivariate distribution of reference and alternative allele counts. Finally, genotypes were called based on simulated genotype quality scores and allele counts using GATK Haplotype Caller, considering a flat prior (1/3 probability for each possible genotype, https://software.broadinstitute.org/gatk/documentation/article?id=11079), and the ratios of alternative allele counts to coverages were calculated.

## 3 Real dataset

We also compared the two competing approaches based on real data from 193 individuals in the 1000 Genomes Project: 101 Yoruba in Ibadan, Nigeria (YRI) and 92 Utah residents with northern and western European ancestry [14]. Variants in the *ASIP* gene on chromosome 20 have been associated with red hair color, freckling, burning, and sun sensitivity [15]. We therefore retrieved called genotype and allele count data on the *ASIP* gene region from a publicly available Variant Call Format (VCF) file. (ftp://ftp.1000genomes.ebi.ac.uk/vol1/ftp/data_collections/1000_genomes_project/working/20170124_grch38_chr20_recall/lc_bams.gatk.20170111.vcf.gz). The real dataset included 398 biallelic variants with complete information on called genotypes and allele counts. Binary phenotypes identified whether the individual was YRI or not (Please note that population stratification, typically a confounding factor in genetic association studies, was the phenotype of interest here).

## 4 Methods

Our goal was to compare two methods for testing genetic association: the standard method that investigates the relationship between called genotypes and phenotypes, and the novel approach that tests the direct association between allele counts and phenotypes, avoiding genotype calling.

We conducted simulations to compare both the standard method and the novel approach with the ideal situation in which real genotypes were known, sometimes referred to as the “Oracle scenario”. In other words, three different associations were tested based on simulated data: (1) between real genotypes as response variable and phenotypes as explanatory variable by ordinal logistic regression; (2) between called genotypes as response variable and phenotypes as explanatory variable by ordinal logistic regression; and (3) between the ratio of alternative allele counts to coverage as response variable and phenotypes as explanatory variable by linear regression (Fig. 1). The type I error rate was calculated in the null scenario and the statistical power was quantified in the alternative scenario for the three investigated associations.

We used not only simulated but also real data to examine the potential improvement in the quality of statistical inference by direct use of allele counts instead of called genotypes. The relationship between genetic variability in the *ASIP* gene region and Yoruban ancestry was evaluated by testing the association between (1) called genotypes as response variable and YRI descent as explanatory variable by ordinal logistic regression; and (2) the ratio of alternative allele counts to coverage as response variable and YRI descent as explanatory variable by linear regression. Probability values for each variant were represented in a Manhattan plot, which was complemented with a linkage disequilibrium (LD) plot to refine the region of interest.

VCFtools (v0.1.13 version) was used to extract the information needed: allele counts (AD field in the FORMAT tag of the VCF file), called genotypes (GT field), and genotype quality scores (GQ field). Coverage was calculated as the sum of the reference and the alternative allele counts. Minor allele frequencies (MAF) were calculated using PLINK (v1.07 version). The computer code in R to reproduce all described calculations is provided as supplementary material.

## 5 Results

**Fig. 2:**
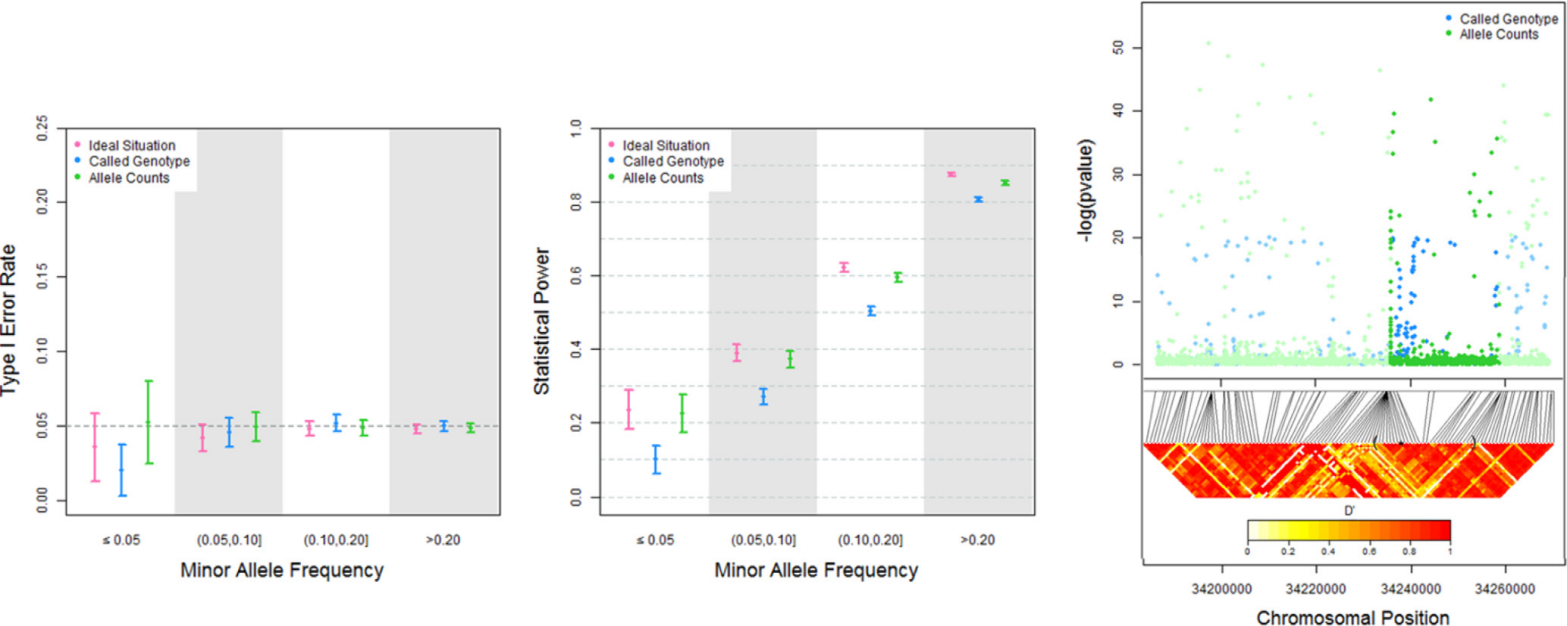
Type I error rate and statistical power for binary phenotypes stratified by minor allele frequency, and Manhattan and LD plots for the *ASIP* gene region.

The median coverage was 22 reads (SD 2.2) in the simulated datasets and 7 reads (SD 1.5) in the dataset from the 1000 Genomes Project. The analysis of simulated data revealed no inflation of type I error rates: in the null scenario the average proportion of probability values lower or equal to 0.05 amounted to 0.0472 for real genotypes, 0.0496 for called genotypes, and 0.0485 for the ratio of alternative allele counts to coverage (Table 1). Fig. 2**A** shows type I error rates stratified by MAF. With the exception of the conservative results for called genotypes and variants with MAF lower than or equal to 0.05, all three evaluated genetic association tests adequately controlled false-positive rates for each MAF category.

**Table 1:** Type I error rate and statistical power for binary phenotypes (overall and stratified by coverage and genotype quality scores).

				**Null scenario**		**Alternative scenario**
**Investigated association**	**Regression model**	**Stratification**		**#pvals**	**%non-missing pvals < 0.05**	**%non-missing pvals < 0.05**
Real genotype ∼ Phenotype	Ordinal logistic	**None**	–	27,139	0.0472	0.7717
Called genotype ∼ Phenotype	Ordinal logistic		–	27,139	0.0496	0.6878
Alternative allele counts/Coverage ∼ Phenotype	Linear		–	27,139	0.0485	0.7487

		**By coverage**	≤21	9315	0.0466	0.6454
Real genotype ∼	Ordinal	**(reads)**	(21,22]	4408	0.0429	0.7867
Phenotype	logistic		(22,24]	9085	0.0488	0.8427
			>24	4331	0.0496	0.8785
			≤21	9315	0.0479	0.5381
Called genotype ∼	Ordinal		(21,22]	4408	0.0449	0.7035
Phenotype	logistic		(22,24]	9085	0.0525	0.7717

			>24	4331	0.0517	0.8181
			≤21	9315	0.0498	0.6218
Alternative allele	Linear		(21,22]	4408	0.0420	0.7641
counts/Coverage ∼			(22,24]	9085	0.0500	0.8187
Phenotype			>24	4331	0.0494	0.8589
		**By genotype**	≤69.2	6831	0.0469	0.5475
Real genotype ∼	Ordinal	**quality**	(69.2,84.1]	6739	0.0432	0.7798
Phenotype	logistic	**(scores)**	(84.1,96.2]	6801	0.0512	0.8615
			>96.2	6768	0.0476	0.8989
			≤69.2	6831	0.0504	0.4264
Called genotype ∼	Ordinal		(69.2,84.1]	6739	0.0453	0.6930
Phenotype	logistic		(84.1,96.2]	6801	0.0538	0.7939
			>96.2	6768	0.0488	0.8400
			≤69.2	6831	0.0505	0.5232
Alternative allele	Linear		(69.2,84.1]	6739	0.0450	0.7601
counts/Coverage ∼			(84.1,96.2]	6801	0.0501	0.8377
			>96.2	6768	0.0485	0.8754

In the alternative scenario the average proportion of probability values lower or equal to 0.05 amounted to 0.77 for real genotypes (maximum attainable statistical power, “Oracle scenario”), 0.69 for called genotypes, and 0.75 for the ratio of alternative allele counts to coverage. Results from power calculations stratified by MAF are shown in Fig. 2**B**. Association tests based on allele counts achieved a higher statistical power than tests based on called genotypes for each MAF category. For example, the average statistical power for variants with MAF lower than or equal to 0.05 was 0.10 for called genotypes, compared with 0.23 for the ratio of alternative allele counts to coverage ([0.1004–0.2249]/0.1004 = 124 % relative increase in statistical power). Calling genotypes using low-coverage sequencing data is computationally challenging [16], and the advantage in statistical power of the novel approach increased with decreasing coverage: the first coverage quartile (Q1) was 21 reads, the third coverage quartile (Q3) was 24 reads, and the relative increase in statistical power amounted to 15.6 % for ≤21 reads [Q1] compared to 5 % for >24 reads [Q3] (Table 1). The advantage in statistical power of the novel approach also increased with decreasing genotype quality (22.7 % power increase for genotype quality score ≤69.2 [Q1] compared to 4.2 % power increase for genotype quality score >96.2 [Q3]). Detailed results stratified by coverage, genotype quality, MAF, reference and alternative allele, and results for quantitative phenotypes are provided as supplementary material (**Tables S1-S8**). For example, in the alternative scenario for continuous phenotypes the average proportion of probability values lower or equal to 0.05 amounted to 0.90 for real genotypes (Oracle scenario), 0.83 for called genotypes, and 0.88 for the ratio of alternative allele counts to coverage (Table S4).

Fig. 2**C** depicts the Manhattan and LD plots for the investigated *ASIP* gene region. Blue dots represent the association between Yoruban ancestry and called genotypes (standard method), while green dots show the relationship between Yoruban ancestry and the ratio of alternative allele counts to coverage (novel approach). The star in the LD plot indicates the chromosomal position of a peak where nearby correlated variants showed consistent association signals. The association peak was evident only when the new approach based on allele counts was used.

## 6 Conclusions

Results based on simulated and real data demonstrate that genetic association tests based on allele counts may result in higher statistical power, with controlled type I error rates, and clearer association signals than the classical investigation of called genotypes. The relative gain in statistical power can be particularly relevant for rare variants and positions with low coverage.

The investigation of differential gene expression based on RNA sequence data commonly relies on count data [17]. By contrast, the direct investigation of allele counts from DNA sequence data is still at a very early stage of development in diploid organisms, including humans [10]. The use of the ratio of alternative allele counts to coverage is better covered in the polyploid literature, especially in plant genetics [18], [19], [20], [21], [22], [23]. We demonstrate here that direct analysis of allele counts may boost the statistical power. It is well known that NGS data are noisy and Hardy–Weinberg equilibrium tests based on called genotypes as well as user-defined filters (for example, setting minimum coverage) are often applied to control data quality. The proposed approach does not preclude the use of complementary quality filters before or after identification of the most promising association signals relying on allele counts. This short communication may guide and motivate the comparison of alternative genotype calling approaches (e.g. different prior probabilities for the Haplotype Caller, Bcftools, VarScan2 or FreeBayes) and different handling of allele counts (e.g. categorisation to assess non-additive genetic effects) in the future [7], [8], [9].

## 7 Author information

RGS analyzed and interpreted the data, created figures and tables, searched the literature, and wrote the first draft of the paper. CF supported data analysis and interpretation and reviewed the manuscript. JLB planned, coordinated, interpreted, and supervised the work. All authors contributed to the final version of the manuscript.

## Funding

Computing-intensive calculations were supported by the high-performance computing initiative in Baden-Württemberg (bwHPC) and the German Research Foundation (DFG) through grant INST 35/1134-1 FUGG and INST 35/1503-1 FUGG. This research was funded by the European Union’s Horizon 2020 research and innovation program (grant 825741). For the publication fee we acknowledge financial support by DFG within the funding programme “Open Access Publikationskosten”, as well as by Heidelberg University. The funders had no role in study design, data collection and analysis, decision to publish or preparation of the manuscript.

## Declaration of Competing Interest

The authors declare that they have no known competing financial interests or personal relationships that could have appeared to influence the work reported in this paper.
