# Supplementary material for: NGS allele counts versus called genotypes for testing genetic association
Source: Comput Struct Biotechnol J. 2022 Jul 11;20:3729–33. doi: 10.1016/j.csbj.2022.07.016 (PMC9294184; doi:10.1016/j.csbj.2022.07.016)
Supplement: Supplementary data 1 [file mmc1.docx]

## Table S1: Type I error rate and statistical power for binary phenotypes (overall and stratified by coverage and genotype quality scores)

|  |  |  |  | **Null scenario** | | | | **Alternative scenario** | | | |
| --- | --- | --- | --- | --- | --- | --- | --- | --- | --- | --- | --- |
| **Investigated association** | **Regression model** | **Stratification** |  | **#pvals** | **#non-missing pvals** | **%non-missing pvals** | **%non-missing pvals < 0.05** | **#pvals** | **#non-missing pvals** | **%non-missing pvals** | **%non-missing pvals < 0.05** |
| Real genotype ~ Phenotype | Ordinal logistic | **None** | - | 27139 | 27114 | 99.91 | 0.0472 | 27139 | 27114 | 99.91 | 0.7717 |
| Called genotype ~ Phenotype | Ordinal logistic |  | - | 27139 | 27139 | 100.00 | 0.0496 | 27139 | 27139 | 100.00 | 0.6878 |
| Alternative allele counts/Coverage ~ Phenotype | Linear |  | - | 27139 | 27139 | 100.00 | 0.0485 | 27139 | 27139 | 100.00 | 0.7487 |
|  |  | **By coverage** | ≤21 | 9315 | 9292 | 99.75 | 0.0466 | 9315 | 9292 | 99.75 | 0.6454 |
| Real genotype ~ | Ordinal | **(reads)** | (21,22] | 4408 | 4406 | 99.95 | 0.0429 | 4408 | 4406 | 99.95 | 0.7867 |
| Phenotype | logistic |  | (22,24] | 9085 | 9085 | 100.00 | 0.0488 | 9085 | 9085 | 100.00 | 0.8427 |
|  |  |  | >24 | 4331 | 4331 | 100.00 | 0.0496 | 4331 | 4331 | 100.00 | 0.8785 |
|  |  |  | ≤21 | 9315 | 9315 | 100.00 | 0.0479 | 9315 | 9315 | 100.00 | 0.5381 |
| Called genotype ~ | Ordinal |  | (21,22] | 4408 | 4408 | 100.00 | 0.0449 | 4408 | 4408 | 100.00 | 0.7035 |
| Phenotype | logistic |  | (22,24] | 9085 | 9085 | 100.00 | 0.0525 | 9085 | 9085 | 100.00 | 0.7717 |
|  |  |  | >24 | 4331 | 4331 | 100.00 | 0.0517 | 4331 | 4331 | 100.00 | 0.8181 |
|  |  |  | ≤21 | 9315 | 9315 | 100.00 | 0.0498 | 9315 | 9315 | 100.00 | 0.6218 |
| Alternative allele | Linear |  | (21,22] | 4408 | 4408 | 100.00 | 0.0420 | 4408 | 4408 | 100.00 | 0.7641 |
| counts/Coverage ~ |  |  | (22,24] | 9085 | 9085 | 100.00 | 0.0500 | 9085 | 9085 | 100.00 | 0.8187 |
| Phenotype |  |  | >24 | 4331 | 4331 | 100.00 | 0.0494 | 4331 | 4331 | 100.00 | 0.8589 |
|  |  | **By genotype** | ≤69.2 | 6831 | 6806 | 99.63 | 0.0469 | 6831 | 6806 | 99.63 | 0.5475 |
| Real genotype ~ | Ordinal | **quality** | (69.2,84.1] | 6739 | 6739 | 100.00 | 0.0432 | 6739 | 6739 | 100.00 | 0.7798 |
| Phenotype | logistic | **(scores)** | (84.1,96.2] | 6801 | 6801 | 100.00 | 0.0512 | 6801 | 6801 | 100.00 | 0.8615 |
|  |  |  | >96.2 | 6768 | 6768 | 100.00 | 0.0476 | 6768 | 6768 | 100.00 | 0.8989 |
|  |  |  | ≤69.2 | 6831 | 6831 | 100.00 | 0.0504 | 6831 | 6831 | 100.00 | 0.4264 |
| Called genotype ~ | Ordinal |  | (69.2,84.1] | 6739 | 6739 | 100.00 | 0.0453 | 6739 | 6739 | 100.00 | 0.6930 |
| Phenotype | logistic |  | (84.1,96.2] | 6801 | 6801 | 100.00 | 0.0538 | 6801 | 6801 | 100.00 | 0.7939 |
|  |  |  | >96.2 | 6768 | 6768 | 100.00 | 0.0488 | 6768 | 6768 | 100.00 | 0.8400 |
|  |  |  | ≤69.2 | 6831 | 6831 | 100.00 | 0.0505 | 6831 | 6831 | 100.00 | 0.5232 |
| Alternative allele | Linear |  | (69.2,84.1] | 6739 | 6739 | 100.00 | 0.0450 | 6739 | 6739 | 100.00 | 0.7601 |
| counts/Coverage ~ |  |  | (84.1,96.2] | 6801 | 6801 | 100.00 | 0.0501 | 6801 | 6801 | 100.00 | 0.8377 |
|  |  |  | >96.2 | 6768 | 6768 | 100.00 | 0.0485 | 6768 | 6768 | 100.00 | 0.8754 |

## Table S2: Type I error rate and statistical power for binary phenotypes stratified by minor allele frequency

|  |  | **MAF** | **Null scenario** | | | | **Alternative scenario** | | | |
| --- | --- | --- | --- | --- | --- | --- | --- | --- | --- | --- |
| **Investigated Association** | **Regression model** |  | **#pvals** | **# non-missing pvals** | **% non-missing pvals** | **% non-missing pvals under 0.05** | **# pvals** | **# non-missing pvals** | **% non-missing pvals** | **% non-missing pvals under 0.05** |
| Real Genotype ~ Phenotype | Ordinal  logistic | ≤0.05 | 249 | 226 | 90.76 | 0.0354 | 249 | 226 | 90.76 | 0.2345 |
|  |  | (0.05,0.1] | 1850 | 1848 | 99.89 | 0.0417 | 1850 | 1848 | 99.89 | 0.3896 |
|  |  | (0.1,0.02] | 6707 | 6707 | 100.00 | 0.0480 | 6707 | 6707 | 100.00 | 0.6204 |
|  |  | >0.2 | 18317 | 18317 | 100.00 | 0.0476 | 18317 | 18317 | 100.00 | 0.8724 |
| Called Genotype ~ Phenotype | Ordinal  logistic | ≤0.05 | 249 | 249 | 100.00 | 0.0201 | 249 | 249 | 100.00 | 0.1004 |
|  |  | (0.05,0.1] | 1850 | 1850 | 100.00 | 0.0454 | 1850 | 1850 | 100.00 | 0.2703 |
|  |  | (0.1,0.02] | 6707 | 6707 | 100.00 | 0.0517 | 6707 | 6707 | 100.00 | 0.5037 |
|  |  | >0.2 | 18317 | 18317 | 100.00 | 0.0496 | 18317 | 18317 | 100.00 | 0.8055 |
| Alternative Allele Counts/ Coverage ~ Phenotype | Linear | ≤0.05 | 249 | 249 | 100.00 | 0.0522 | 249 | 249 | 100.00 | 0.2249 |
|  |  | (0.05,0.1] | 1850 | 1850 | 100.00 | 0.0492 | 1850 | 1850 | 100.00 | 0.3719 |
|  |  | (0.1,0.02] | 6707 | 6707 | 100.00 | 0.0485 | 6707 | 6707 | 100.00 | 0.5928 |
|  |  | >0.2 | 18317 | 18317 | 100.00 | 0.0484 | 18317 | 18317 | 100.00 | 0.8508 |

## Table S3: Type I error rate and statistical power for binary phenotypes stratified by reference and alternative alleles

|  |  | **Reference and**  **alternative alleles** | **Null scenario** | | | | **Alternative scenario** | | | |
| --- | --- | --- | --- | --- | --- | --- | --- | --- | --- | --- |
| **Investigated Association** | **Regression model** |  | **#pvals** | **# non-missing pvals** | **% non-missing pvals** | **% non-missing pvals under 0.05** | **# pvals** | **# non-missing pvals** | **% non-missing pvals** | **% non-missing pvals under 0.05** |
| Real Genotype ~ Phenotype | Ordinal  logistic | A/C | 2197 | 2191 | 99.73 | 0.0493 | 2197 | 2191 | 99.73 | 0.7910 |
|  |  | A/G | 10417 | 10410 | 99.93 | 0.0456 | 10417 | 10410 | 99.93 | 0.7698 |
|  |  | A/T | 833 | 833 | 100.00 | 0.0588 | 833 | 833 | 100.00 | 0.7671 |
|  |  | C/G | 1218 | 1217 | 99.92 | 0.0460 | 1218 | 1217 | 99.92 | 0.7790 |
|  |  | C/T | 10275 | 10264 | 99.89 | 0.0464 | 10275 | 10264 | 99.89 | 0.7708 |
|  |  | G/T | 2199 | 2199 | 100.00 | 0.0528 | 2199 | 2199 | 100.00 | 0.7631 |
| Called Genotype ~ Phenotype | Ordinal  logistic | A/C | 2197 | 2197 | 100.00 | 0.0514 | 2197 | 2197 | 100.00 | 0.6896 |
|  |  | A/G | 10417 | 10417 | 100.00 | 0.0504 | 10417 | 10417 | 100.00 | 0.6888 |
|  |  | A/T | 833 | 833 | 100.00 | 0.0660 | 833 | 833 | 100.00 | 0.6891 |
|  |  | C/G | 1218 | 1218 | 100.00 | 0.0583 | 1218 | 1218 | 100.00 | 0.6913 |
|  |  | C/T | 10275 | 10275 | 100.00 | 0.0456 | 10275 | 10275 | 100.00 | 0.6910 |
|  |  | G/T | 2199 | 2199 | 100.00 | 0.0509 | 2199 | 2199 | 100.00 | 0.6644 |
| Alternative Allele Counts/ Coverage ~ Phenotype | Linear | A/C | 2197 | 2197 | 100.00 | 0.0487 | 2197 | 2197 | 100.00 | 0.7510 |
|  |  | A/G | 10417 | 10417 | 100.00 | 0.0497 | 10417 | 10417 | 100.00 | 0.7498 |
|  |  | A/T | 833 | 833 | 100.00 | 0.0552 | 833 | 833 | 100.00 | 0.7371 |
|  |  | C/G | 1218 | 1218 | 100.00 | 0.0501 | 1218 | 1218 | 100.00 | 0.7455 |
|  |  | C/T | 10275 | 10275 | 100.00 | 0.0448 | 10275 | 10275 | 100.00 | 0.7495 |
|  |  | G/T | 2199 | 2199 | 100.00 | 0.0568 | 2199 | 2199 | 100.00 | 0.7431 |

## Table S4: Type I error rate and statistical power for continuous phenotypes

|  |  | **Null scenario** | | | | **Alternative scenario** | | | |
| --- | --- | --- | --- | --- | --- | --- | --- | --- | --- |
| **Investigated Association** | **Regression model** | **#pvals** | **# non-missing pvals** | **% non-missing pvals** | **% non-missing pvals under 0.05** | **# pvals** | **# non-missing pvals** | **% non-missing pvals** | **% non-missing pvals under 0.05** |
| Real Genotype ~ Phenotype | Ordinal  logistic | 27139 | 27114 | 99.91 | 0.0498 | 27139 | 27114 | 99.91 | 0.8958 |
| Called Genotype ~ Phenotype | Ordinal  logistic | 27139 | 27139 | 100.00 | 0.0508 | 27139 | 27139 | 100.00 | 0.8287 |
| Alternative Allele Counts/ Coverage ~ Phenotype | Linear | 27139 | 27139 | 100.00 | 0.0505 | 27139 | 27139 | 100.00 | 0.8777 |

## Table S5: Type I error rate and statistical power for continuous phenotypes stratified by minor allele frequency

|  |  | **MAF** | **Null scenario** | | | | **Alternative scenario** | | | |
| --- | --- | --- | --- | --- | --- | --- | --- | --- | --- | --- |
| **Investigated Association** | **Regression model** |  | **#pvals** | **# non-missing pvals** | **% non-missing pvals** | **% non-missing pvals under 0.05** | **# pvals** | **# non-missing pvals** | **% non-missing pvals** | **% non-missing pvals under 0.05** |
| Real Genotype ~ Phenotype | Ordinal  logistic | ≤0.05 | 249 | 226 | 90.76 | 0.0487 | 249 | 226 | 90.76 | 0.3628 |
|  |  | (0.05,0.1] | 1850 | 1848 | 99.89 | 0.0444 | 1850 | 1848 | 99.89 | 0.5639 |
|  |  | (0.1,0.02] | 6707 | 6707 | 100.00 | 0.0504 | 6707 | 6707 | 100.00 | 0.8118 |
|  |  | >0.2 | 18317 | 18317 | 100.00 | 0.0502 | 18317 | 18317 | 100.00 | 0.9666 |
| Called Genotype ~ Phenotype | Ordinal  logistic | ≤0.05 | 249 | 249 | 100.00 | 0.0562 | 249 | 249 | 100.00 | 0.1888 |
|  |  | (0.05,0.1] | 1850 | 1850 | 100.00 | 0.0427 | 1850 | 1850 | 100.00 | 0.4043 |
|  |  | (0.1,0.02] | 6707 | 6707 | 100.00 | 0.0543 | 6707 | 6707 | 100.00 | 0.6893 |
|  |  | >0.2 | 18317 | 18317 | 100.00 | 0.0504 | 18317 | 18317 | 100.00 | 0.9315 |
| Alternative Allele Counts/ Coverage ~ Phenotype | Linear | ≤0.05 | 249 | 249 | 100.00 | 0.0562 | 249 | 249 | 100.00 | 0.3133 |
|  |  | (0.05,0.1] | 1850 | 1850 | 100.00 | 0.0486 | 1850 | 1850 | 100.00 | 0.5162 |
|  |  | (0.1,0.02] | 6707 | 6707 | 100.00 | 0.0522 | 6707 | 6707 | 100.00 | 0.7831 |
|  |  | >0.2 | 18317 | 18317 | 100.00 | 0.0500 | 18317 | 18317 | 100.00 | 0.9567 |

## Table S6: Type I error rate and statistical power for continuous phenotypes stratified by coverage

|  |  | **Coverage** | **Null scenario** | | | | **Alternative scenario** | | | |
| --- | --- | --- | --- | --- | --- | --- | --- | --- | --- | --- |
| **Investigated Association** | **Regression model** |  | **#pvals** | **# non-missing pvals** | **% non-missing pvals** | **% non-missing pvals under 0.05** | **# pvals** | **# non-missing pvals** | **% non-missing pvals** | **% non-missing pvals under 0.05** |
| Real Genotype ~ Phenotype | Ordinal  logistic | ≤21 | 9315 | 9292 | 99.75 | 0.0476 | 9315 | 9292 | 99.75 | 0.8031 |
|  |  | (21,22] | 4408 | 4406 | 99.95 | 0.0477 | 4408 | 4406 | 99.95 | 0.9117 |
|  |  | (22,24] | 9085 | 9085 | 100.00 | 0.0534 | 9085 | 9085 | 100.00 | 0.9482 |
|  |  | >24 | 4331 | 4331 | 100.00 | 0.0494 | 4331 | 4331 | 100.00 | 0.9688 |
| Called Genotype ~ Phenotype | Ordinal  logistic | ≤21 | 9315 | 9315 | 100.00 | 0.0492 | 9315 | 9315 | 100.00 | 0.6924 |
|  |  | (21,22] | 4408 | 4408 | 100.00 | 0.0508 | 4408 | 4408 | 100.00 | 0.8514 |
|  |  | (22,24] | 9085 | 9085 | 100.00 | 0.0533 | 9085 | 9085 | 100.00 | 0.9052 |
|  |  | >24 | 4331 | 4331 | 100.00 | 0.0494 | 4331 | 4331 | 100.00 | 0.9384 |
| Alternative Allele Counts/ Coverage ~ Phenotype | Linear | ≤21 | 9315 | 9315 | 100.00 | 0.0486 | 9315 | 9315 | 100.00 | 0.7733 |
|  |  | (21,22] | 4408 | 4408 | 100.00 | 0.0490 | 4408 | 4408 | 100.00 | 0.8938 |
|  |  | (22,24] | 9085 | 9085 | 100.00 | 0.0539 | 9085 | 9085 | 100.00 | 0.9376 |
|  |  | >24 | 4331 | 4331 | 100.00 | 0.0487 | 4331 | 4331 | 100.00 | 0.9603 |

## Table S7: Type I error rate and statistical power for continuous phenotypes stratified by genotype quality scores

|  |  | **Genotype**  **quality** | **Null scenario** | | | | **Alternative scenario** | | | |
| --- | --- | --- | --- | --- | --- | --- | --- | --- | --- | --- |
| **Investigated Association** | **Regression model** |  | **#pvals** | **# non-missing pvals** | **% non-missing pvals** | **% non-missing pvals under 0.05** | **# pvals** | **# non-missing pvals** | **% non-missing pvals** | **% non-missing pvals under 0.05** |
| Real Genotype ~ Phenotype | Ordinal  logistic | ≤69.2 | 9315 | 9292 | 99.75 | 0.0466 | 9315 | 9292 | 99.75 | 0.6454 |
|  |  | (69.2,84.1] | 4408 | 4406 | 99.95 | 0.0429 | 4408 | 4406 | 99.95 | 0.7867 |
|  |  | (84.1,96.2] | 9085 | 9085 | 100.00 | 0.0488 | 9085 | 9085 | 100.00 | 0.8427 |
|  |  | >96.2 | 4331 | 4331 | 100.00 | 0.0496 | 4331 | 4331 | 100.00 | 0.8785 |
| Called Genotype ~ Phenotype | Ordinal  logistic | ≤69.2 | 9315 | 9315 | 100.00 | 0.0479 | 9315 | 9315 | 100.00 | 0.5381 |
|  |  | (69.2,84.1] | 4408 | 4408 | 100.00 | 0.0449 | 4408 | 4408 | 100.00 | 0.7035 |
|  |  | (84.1,96.2] | 9085 | 9085 | 100.00 | 0.0525 | 9085 | 9085 | 100.00 | 0.7717 |
|  |  | >96.2 | 4331 | 4331 | 100.00 | 0.0517 | 4331 | 4331 | 100.00 | 0.8181 |
| Alternative Allele Counts/ Coverage ~ Phenotype | Linear | ≤69.2 | 9315 | 9315 | 100.00 | 0.0498 | 9315 | 9315 | 100.00 | 0.6218 |
|  |  | (69.2,84.1] | 4408 | 4408 | 100.00 | 0.0420 | 4408 | 4408 | 100.00 | 0.7641 |
|  |  | (84.1,96.2] | 9085 | 9085 | 100.00 | 0.0500 | 9085 | 9085 | 100.00 | 0.8187 |
|  |  | >96.2 | 4331 | 4331 | 100.00 | 0.0494 | 4331 | 4331 | 100.00 | 0.8589 |

## Table S8: Type I error rate and statistical power for continuous phenotypes stratified by reference and alternative alleles

|  |  | **Reference and**  **alternative alleles** | **Null scenario** | | | | **Alternative scenario** | | | |
| --- | --- | --- | --- | --- | --- | --- | --- | --- | --- | --- |
| **Investigated Association** | **Regression model** |  | **#pvals** | **# non-missing pvals** | **% non-missing pvals** | **% non-missing pvals under 0.05** | **# pvals** | **# non-missing pvals** | **% non-missing pvals** | **% non-missing pvals under 0.05** |
| Real Genotype ~ Phenotype | Ordinal  logistic | A/C | 2197 | 2191 | 99.73 | 0.0507 | 2197 | 2191 | 99.73 | 0.9060 |
|  |  | A/G | 10417 | 10410 | 99.93 | 0.0495 | 10417 | 10410 | 99.93 | 0.8969 |
|  |  | A/T | 833 | 833 | 100.00 | 0.0588 | 833 | 833 | 100.00 | 0.8872 |
|  |  | C/G | 1218 | 1217 | 99.92 | 0.0452 | 1218 | 1217 | 99.92 | 0.9039 |
|  |  | C/T | 10275 | 10264 | 99.89 | 0.0506 | 10275 | 10264 | 99.89 | 0.8933 |
|  |  | G/T | 2199 | 2199 | 100.00 | 0.0464 | 2199 | 2199 | 100.00 | 0.8909 |
| Called Genotype ~ Phenotype | Ordinal  logistic | A/C | 2197 | 2197 | 100.00 | 0.0496 | 2197 | 2197 | 100.00 | 0.8284 |
|  |  | A/G | 10417 | 10417 | 100.00 | 0.0501 | 10417 | 10417 | 100.00 | 0.8306 |
|  |  | A/T | 833 | 833 | 100.00 | 0.0612 | 833 | 833 | 100.00 | 0.8079 |
|  |  | C/G | 1218 | 1218 | 100.00 | 0.0468 | 1218 | 1218 | 100.00 | 0.8383 |
|  |  | C/T | 10275 | 10275 | 100.00 | 0.0506 | 10275 | 10275 | 100.00 | 0.8325 |
|  |  | G/T | 2199 | 2199 | 100.00 | 0.0550 | 2199 | 2199 | 100.00 | 0.8054 |
| Alternative Allele Counts/ Coverage ~ Phenotype | Linear | A/C | 2197 | 2197 | 100.00 | 0.0487 | 2197 | 2197 | 100.00 | 0.8817 |
|  |  | A/G | 10417 | 10417 | 100.00 | 0.0506 | 10417 | 10417 | 100.00 | 0.8809 |
|  |  | A/T | 833 | 833 | 100.00 | 0.0624 | 833 | 833 | 100.00 | 0.8691 |
|  |  | C/G | 1218 | 1218 | 100.00 | 0.0476 | 1218 | 1218 | 100.00 | 0.8793 |
|  |  | C/T | 10275 | 10275 | 100.00 | 0.0494 | 10275 | 10275 | 100.00 | 0.8763 |
|  |  | G/T | 2199 | 2199 | 100.00 | 0.0537 | 2199 | 2199 | 100.00 | 0.8677 |

## Computer code in the R language to reproduce all described calculations

#simulation of the alternative scenario

#function for the estimation of GT based on the Haplotype caller from GATK

estim_gt<-function(data){

data<-cbind(data,GT="5/5")

data$GT<-as.character(data$GT)

for (i in 1:length(data[,1])){

hom_ref<-(1/3)*((1-10^(-data$GQ[i]/10))^data$AD1[i])*((10^(-data$GQ[i]/10))^data$AD2[i])

het<-(1/3)*(((1)/2)^data$AD1[i])*(((1)/2)^data$AD2[i])

hom_alt<-(1/3)*((10^(-data$GQ[i]/10))^data$AD1[i])*((1-10^(-data$GQ[i]/10))^data$AD2[i])

if(which.max(c(hom_ref,het,hom_alt))==1) {data[i,4]<-"0/0"}

if(which.max(c(hom_ref,het,hom_alt))==2) {data[i,4]<-as.character("0/1")}

if(which.max(c(hom_ref,het,hom_alt))==3) {data[i,4]<-"1/1"}

}

return(data)

}

#loading libraries

print("loading libraries")

library("tmvtnorm")

library(robust)

library(pscl)

library(gamlss.dist)

library("grDevices", lib.loc="~/R/R-3.2.2/library")

Sys.setenv(LANG = "en")

#to save the results

a<-Sys.time()

results_pvalue<-data.frame(rs=0,

alleles=0,

chrom=0,

pos=0,

ad2_ph_poisson=0,

ad2_ph_nb=0,

ad2_ph_nb_zi=0,

ad2_ph_nb_hur=0,

ratio_ph_lm=0,

ph_ratio_lm=0,

gt_ph_ordinal_logit=0,

gt_ph_logit=0,

ph_gt_lm=0,

gtr_ph_ordinal_logit=0,

gtr_ph_logit=0,

ph_gtr_lm=0,

ad2_phd_poisson=0,

ad2_phd_nb=0,

ad2_phd_nb_zi=0,

ad2_phd_nb_hur=0,

ratio_phd_lm=0,

phd_ratio_logit=0,

gt_phd_ordinal_logit=0,

gt_phd_logit=0,

phd_gt_logit=0,

gtr_phd_ordinal_logit=0,

gtr_phd_logit=0,

phd_gtr_logit=0)

results_estim<-data.frame(rs=0,

alleles=0,

chrom=0,

pos=0,

e_ad2_phd_poisson_beta0=0,

e_ad2_phd_poisson_beta0_se=0,

e_ad2_phd_poisson_beta1=0,

e_ad2_phd_poisson_beta1_se=0,

e_ad2_phd_nb_beta0=0,

e_ad2_phd_nb_beta0_se=0,

e_ad2_phd_nb_beta1=0,

e_ad2_phd_nb_beta1_se=0,

e_ad2_phd_nb_zi_beta0c=0,

e_ad2_phd_nb_zi_beta0c_se=0,

e_ad2_phd_nb_zi_beta1c=0,

e_ad2_phd_nb_zi_beta1c_se=0,

e_ad2_phd_nb_zi_logthetac=0,

e_ad2_phd_nb_zi_logthetac_se=0,

e_ad2_phd_nb_zi_beta0z=0,

e_ad2_phd_nb_zi_beta0z_se=0,

e_ad2_phd_nb_zi_beta1z=0,

e_ad2_phd_nb_zi_beta1z_se=0,

e_ad2_phd_nb_hur_beta0c=0,

e_ad2_phd_nb_hur_beta0c_se=0,

e_ad2_phd_nb_hur_beta1c=0,

e_ad2_phd_nb_hur_beta1c_se=0,

e_ad2_phd_nb_hur_logthetac=0,

e_ad2_phd_nb_hur_logthetac_se=0,

e_ad2_phd_nb_hur_beta0z=0,

e_ad2_phd_nb_hur_beta0z_se=0,

e_ad2_phd_nb_hur_beta1z=0,

e_ad2_phd_nb_hur_beta1z_se=0,

e_ratio_phd_lm_beta0=0,

e_ratio_phd_lm_beta0_se=0,

e_ratio_phd_lm_beta1=0,

e_ratio_phd_lm_beta1_se=0,

e_phd_ratio_logit_beta0=0,

e_phd_ratio_logit_beta0_se=0,

e_phd_ratio_logit_beta1=0,

e_phd_ratio_logit_beta1_se=0,

e_gt_phd_ordinal_logit_coef=0,

e_gt_phd_ordinal_logit_coef_se=0,

e_gt_phd_ordinal_logit_int1=0,

e_gt_phd_ordinal_logit_int1_se=0,

e_gt_phd_ordinal_logit_int2=0,

e_gt_phd_ordinal_logit_int2_se=0,

e_gt_phd_logit_beta0=0,

e_gt_phd_logit_beta0_se=0,

e_gt_phd_logit_beta1=0,

e_gt_phd_logit_beta1_se=0,

e_phd_gt_logit_beta0=0,

e_phd_gt_logit_beta0_se=0,

e_phd_gt_logit_beta1=0,

e_phd_gt_logit_beta1_se=0,

e_gtr_phd_ordinal_logit_coef=0,

e_gtr_phd_ordinal_logit_coef_se=0,

e_gtr_phd_ordinal_logit_int1=0,

e_gtr_phd_ordinal_logit_int1_se=0,

e_gtr_phd_ordinal_logit_int2=0,

e_gtr_phd_ordinal_logit_int2_se=0,

e_gtr_phd_logit_beta0=0,

e_gtr_phd_logit_beta0_se=0,

e_gtr_phd_logit_beta1=0,

e_gtr_phd_logit_beta1_se=0,

e_phd_gtr_logit_beta0=0,

e_phd_gtr_logit_beta0_se=0,

e_phd_gtr_logit_beta1=0,

e_phd_gtr_logit_beta1_se=0)

results_variant<-data.frame(rs=0,

alleles=0,

chrom=0,

pos=0,

medianGQ=0,

medianSAD=0)

results_table<-data.frame(rs=0,

alleles=0,

chrom=0,

pos=0,

r0e0=0,

r0e1=0,

r0e2=0,

r1e0=0,

r1e1=0,

r1e2=0,

r2e0=0,

r2e1=0,

r2e2=0)

#read real genotypes

print("reading files")

gt<- read.table("gt_simulated.txt", header=TRUE, quote="\"")

#preparation

print("preparing files")

#CC TT CT

#read and modify lists

C_T_CT <- read.table("C_T_CT.txt", header=TRUE, quote="\"")

C_T_TT <- read.table("C_T_TT.txt", header=TRUE, quote="\"")

C_T_CC <- read.table("C_T_CC.txt", header=TRUE, quote="\"")

#make c_T_CC in the correct order

C_T_CC<-data.frame(C_T_CC[,1:4],AD1=C_T_CC$AD1,AD2=C_T_CC$AD2,C_T_CC[,5:7])

#modify AD1 and AD2

C_T_CT<-data.frame(C_T_CT,AD=paste(C_T_CT$AD1,",",C_T_CT$AD2,",",C_T_CT$GQ))

C_T_CT$AD<-as.character(C_T_CT$AD)

C_T_CT$AD1<-as.numeric(C_T_CT$AD1)

C_T_CT$AD2<-as.numeric(C_T_CT$AD2)

C_T_CT$GQ<-as.numeric(C_T_CT$GQ)

C_T_CC<-data.frame(C_T_CC,AD=paste(C_T_CC$AD1,",",C_T_CC$AD2,",",C_T_CC$GQ))

C_T_CC$AD<-as.character(C_T_CC$AD)

C_T_CC$AD1<-as.numeric(C_T_CC$AD1)

C_T_CC$AD2<-as.numeric(C_T_CC$AD2)

C_T_CC$GQ<-as.numeric(C_T_CC$GQ)

C_T_TT<-data.frame(C_T_TT,AD=paste(C_T_TT$AD1,",",C_T_TT$AD2,",",C_T_TT$GQ))

C_T_TT$AD<-as.character(C_T_TT$AD)

C_T_TT$AD1<-as.numeric(C_T_TT$AD1)

C_T_TT$AD2<-as.numeric(C_T_TT$AD2)

C_T_TT$GQ<-as.numeric(C_T_TT$GQ)

for( i in 1:length(C_T_CT$GQ)){

if(C_T_CT$GQ[i]<=20){C_T_CT$G_GQ[i]<-1}

if(C_T_CT$GQ[i]>20&&C_T_CT$GQ[i]<=40){C_T_CT$G_GQ[i]<-2}

if(C_T_CT$GQ[i]>40&&C_T_CT$GQ[i]<=60){C_T_CT$G_GQ[i]<-3}

if(C_T_CT$GQ[i]>60&&C_T_CT$GQ[i]<=80){C_T_CT$G_GQ[i]<-4}

if(C_T_CT$GQ[i]>80&&C_T_CT$GQ[i]<99){C_T_CT$G_GQ[i]<-5}

if(C_T_CT$GQ[i]>=99){C_T_CT$G_GQ[i]<-6}

}

f_GQ_C_T_CT<-table(C_T_CT$G_GQ)/length(C_T_CT$G_GQ)

for( i in 1:length(C_T_CC$GQ)){

if(C_T_CC$GQ[i]<=20){C_T_CC$G_GQ[i]<-1}

if(C_T_CC$GQ[i]>20&&C_T_CC$GQ[i]<=40){C_T_CC$G_GQ[i]<-2}

if(C_T_CC$GQ[i]>40&&C_T_CC$GQ[i]<=60){C_T_CC$G_GQ[i]<-3}

if(C_T_CC$GQ[i]>60&&C_T_CC$GQ[i]<=80){C_T_CC$G_GQ[i]<-4}

if(C_T_CC$GQ[i]>80&&C_T_CC$GQ[i]<99){C_T_CC$G_GQ[i]<-5}

if(C_T_CC$GQ[i]>=99){C_T_CC$G_GQ[i]<-6}

}

f_GQ_C_T_CC<-table(C_T_CC$G_GQ)/length(C_T_CC$G_GQ)

for( i in 1:length(C_T_TT$GQ)){

if(C_T_TT$GQ[i]<=20){C_T_TT$G_GQ[i]<-1}

if(C_T_TT$GQ[i]>20&&C_T_TT$GQ[i]<=40){C_T_TT$G_GQ[i]<-2}

if(C_T_TT$GQ[i]>40&&C_T_TT$GQ[i]<=60){C_T_TT$G_GQ[i]<-3}

if(C_T_TT$GQ[i]>60&&C_T_TT$GQ[i]<=80){C_T_TT$G_GQ[i]<-4}

if(C_T_TT$GQ[i]>80&&C_T_TT$GQ[i]<99){C_T_TT$G_GQ[i]<-5}

if(C_T_TT$GQ[i]>=99){C_T_TT$G_GQ[i]<-6}

}

f_GQ_C_T_TT<-table(C_T_TT$G_GQ)/length(C_T_TT$G_GQ)

#GG TT GT

#read and modify lists

G_T_GT <- read.table("G_T_GT.txt", header=TRUE, quote="\"")

G_T_TT <- read.table("G_T_TT.txt", header=TRUE, quote="\"")

G_T_GG <- read.table("G_T_GG.txt", header=TRUE, quote="\"")

#make c_T_CC in the correct order

G_T_GG<-data.frame(G_T_GG[,1:4],AD1=G_T_GG$AD1,AD2=G_T_GG$AD2,G_T_GG[,5:7])

#modify AD1 and AD2

G_T_GT<-data.frame(G_T_GT,AD=paste(G_T_GT$AD1,",",G_T_GT$AD2,",",G_T_GT$GQ))

G_T_GT$AD<-as.character(G_T_GT$AD)

G_T_GT$AD1<-as.numeric(G_T_GT$AD1)

G_T_GT$AD2<-as.numeric(G_T_GT$AD2)

G_T_GT$GQ<-as.numeric(G_T_GT$GQ)

G_T_GG<-data.frame(G_T_GG,AD=paste(G_T_GG$AD1,",",G_T_GG$AD2,",",G_T_GG$GQ))

G_T_GG$AD<-as.character(G_T_GG$AD)

G_T_GG$AD1<-as.numeric(G_T_GG$AD1)

G_T_GG$AD2<-as.numeric(G_T_GG$AD2)

G_T_GG$GQ<-as.numeric(G_T_GG$GQ)

G_T_TT<-data.frame(G_T_TT,AD=paste(G_T_TT$AD1,",",G_T_TT$AD2,",",G_T_TT$GQ))

G_T_TT$AD<-as.character(G_T_TT$AD)

G_T_TT$AD1<-as.numeric(G_T_TT$AD1)

G_T_TT$AD2<-as.numeric(G_T_TT$AD2)

G_T_TT$GQ<-as.numeric(G_T_TT$GQ)

for( i in 1:length(G_T_GT$GQ)){

if(G_T_GT$GQ[i]<=20){G_T_GT$G_GQ[i]<-1}

if(G_T_GT$GQ[i]>20&&G_T_GT$GQ[i]<=40){G_T_GT$G_GQ[i]<-2}

if(G_T_GT$GQ[i]>40&&G_T_GT$GQ[i]<=60){G_T_GT$G_GQ[i]<-3}

if(G_T_GT$GQ[i]>60&&G_T_GT$GQ[i]<=80){G_T_GT$G_GQ[i]<-4}

if(G_T_GT$GQ[i]>80&&G_T_GT$GQ[i]<99){G_T_GT$G_GQ[i]<-5}

if(G_T_GT$GQ[i]>=99){G_T_GT$G_GQ[i]<-6}

}

f_GQ_G_T_GT<-table(G_T_GT$G_GQ)/length(G_T_GT$G_GQ)

for( i in 1:length(G_T_GG$GQ)){

if(G_T_GG$GQ[i]<=20){G_T_GG$G_GQ[i]<-1}

if(G_T_GG$GQ[i]>20&&G_T_GG$GQ[i]<=40){G_T_GG$G_GQ[i]<-2}

if(G_T_GG$GQ[i]>40&&G_T_GG$GQ[i]<=60){G_T_GG$G_GQ[i]<-3}

if(G_T_GG$GQ[i]>60&&G_T_GG$GQ[i]<=80){G_T_GG$G_GQ[i]<-4}

if(G_T_GG$GQ[i]>80&&G_T_GG$GQ[i]<99){G_T_GG$G_GQ[i]<-5}

if(G_T_GG$GQ[i]>=99){G_T_GG$G_GQ[i]<-6}

}

f_GQ_G_T_GG<-table(G_T_GG$G_GQ)/length(G_T_GG$G_GQ)

for( i in 1:length(G_T_TT$GQ)){

if(G_T_TT$GQ[i]<=20){G_T_TT$G_GQ[i]<-1}

if(G_T_TT$GQ[i]>20&&G_T_TT$GQ[i]<=40){G_T_TT$G_GQ[i]<-2}

if(G_T_TT$GQ[i]>40&&G_T_TT$GQ[i]<=60){G_T_TT$G_GQ[i]<-3}

if(G_T_TT$GQ[i]>60&&G_T_TT$GQ[i]<=80){G_T_TT$G_GQ[i]<-4}

if(G_T_TT$GQ[i]>80&&G_T_TT$GQ[i]<99){G_T_TT$G_GQ[i]<-5}

if(G_T_TT$GQ[i]>=99){G_T_TT$G_GQ[i]<-6}

}

f_GQ_G_T_TT<-table(G_T_TT$G_GQ)/length(G_T_TT$G_GQ)

#CG GG CC

#read and modify lists

C_G_CG <- read.table("C_G_CG.txt", header=TRUE, quote="\"")

C_G_GG <- read.table("C_G_GG.txt", header=TRUE, quote="\"")

C_G_CC <- read.table("C_G_CC.txt", header=TRUE, quote="\"")

#make c_T_CC in the correct order

C_G_CC<-data.frame(C_G_CC[,1:4],AD1=C_G_CC$AD1,AD2=C_G_CC$AD2,C_G_CC[,5:7])

#modify AD1 and AD2

C_G_CG<-data.frame(C_G_CG,AD=paste(C_G_CG$AD1,",",C_G_CG$AD2,",",C_G_CG$GQ))

C_G_CG$AD<-as.character(C_G_CG$AD)

C_G_CG$AD1<-as.numeric(C_G_CG$AD1)

C_G_CG$AD2<-as.numeric(C_G_CG$AD2)

C_G_CG$GQ<-as.numeric(C_G_CG$GQ)

C_G_CC<-data.frame(C_G_CC,AD=paste(C_G_CC$AD1,",",C_G_CC$AD2,",",C_G_CC$GQ))

C_G_CC$AD<-as.character(C_G_CC$AD)

C_G_CC$AD1<-as.numeric(C_G_CC$AD1)

C_G_CC$AD2<-as.numeric(C_G_CC$AD2)

C_G_CC$GQ<-as.numeric(C_G_CC$GQ)

C_G_GG<-data.frame(C_G_GG,AD=paste(C_G_GG$AD1,",",C_G_GG$AD2,",",C_G_GG$GQ))

C_G_GG$AD<-as.character(C_G_GG$AD)

C_G_GG$AD1<-as.numeric(C_G_GG$AD1)

C_G_GG$AD2<-as.numeric(C_G_GG$AD2)

C_G_GG$GQ<-as.numeric(C_G_GG$GQ)

for( i in 1:length(C_G_CG$GQ)){

if(C_G_CG$GQ[i]<=20){C_G_CG$G_GQ[i]<-1}

if(C_G_CG$GQ[i]>20&&C_G_CG$GQ[i]<=40){C_G_CG$G_GQ[i]<-2}

if(C_G_CG$GQ[i]>40&&C_G_CG$GQ[i]<=60){C_G_CG$G_GQ[i]<-3}

if(C_G_CG$GQ[i]>60&&C_G_CG$GQ[i]<=80){C_G_CG$G_GQ[i]<-4}

if(C_G_CG$GQ[i]>80&&C_G_CG$GQ[i]<99){C_G_CG$G_GQ[i]<-5}

if(C_G_CG$GQ[i]>=99){C_G_CG$G_GQ[i]<-6}

}

f_GQ_C_G_CG<-table(C_G_CG$G_GQ)/length(C_G_CG$G_GQ)

for( i in 1:length(C_G_CC$GQ)){

if(C_G_CC$GQ[i]<=20){C_G_CC$G_GQ[i]<-1}

if(C_G_CC$GQ[i]>20&&C_G_CC$GQ[i]<=40){C_G_CC$G_GQ[i]<-2}

if(C_G_CC$GQ[i]>40&&C_G_CC$GQ[i]<=60){C_G_CC$G_GQ[i]<-3}

if(C_G_CC$GQ[i]>60&&C_G_CC$GQ[i]<=80){C_G_CC$G_GQ[i]<-4}

if(C_G_CC$GQ[i]>80&&C_G_CC$GQ[i]<99){C_G_CC$G_GQ[i]<-5}

if(C_G_CC$GQ[i]>=99){C_G_CC$G_GQ[i]<-6}

}

f_GQ_C_G_CC<-table(C_G_CC$G_GQ)/length(C_G_CC$G_GQ)

for( i in 1:length(C_G_GG$GQ)){

if(C_G_GG$GQ[i]<=20){C_G_GG$G_GQ[i]<-1}

if(C_G_GG$GQ[i]>20&&C_G_GG$GQ[i]<=40){C_G_GG$G_GQ[i]<-2}

if(C_G_GG$GQ[i]>40&&C_G_GG$GQ[i]<=60){C_G_GG$G_GQ[i]<-3}

if(C_G_GG$GQ[i]>60&&C_G_GG$GQ[i]<=80){C_G_GG$G_GQ[i]<-4}

if(C_G_GG$GQ[i]>80&&C_G_GG$GQ[i]<99){C_G_GG$G_GQ[i]<-5}

if(C_G_GG$GQ[i]>=99){C_G_GG$G_GQ[i]<-6}

}

f_GQ_C_G_GG<-table(C_G_GG$G_GQ)/length(C_G_GG$G_GQ)

#AA TT AT

#read and modify lists

A_T_AT <- read.table("A_T_AT.txt", header=TRUE, quote="\"")

A_T_TT <- read.table("A_T_TT.txt", header=TRUE, quote="\"")

A_T_AA <- read.table("A_T_AA.txt", header=TRUE, quote="\"")

#make c_T_CC in the correct order

A_T_AA<-data.frame(A_T_AA[,1:4],AD1=A_T_AA$AD1,AD2=A_T_AA$AD2,A_T_AA[,5:7])

#modify AD1 and AD2

A_T_AT<-data.frame(A_T_AT,AD=paste(A_T_AT$AD1,",",A_T_AT$AD2,",",A_T_AT$GQ))

A_T_AT$AD<-as.character(A_T_AT$AD)

A_T_AT$AD1<-as.numeric(A_T_AT$AD1)

A_T_AT$AD2<-as.numeric(A_T_AT$AD2)

A_T_AT$GQ<-as.numeric(A_T_AT$GQ)

A_T_AA<-data.frame(A_T_AA,AD=paste(A_T_AA$AD1,",",A_T_AA$AD2,",",A_T_AA$GQ))

A_T_AA$AD<-as.character(A_T_AA$AD)

A_T_AA$AD1<-as.numeric(A_T_AA$AD1)

A_T_AA$AD2<-as.numeric(A_T_AA$AD2)

A_T_AA$GQ<-as.numeric(A_T_AA$GQ)

A_T_TT<-data.frame(A_T_TT,AD=paste(A_T_TT$AD1,",",A_T_TT$AD2,",",A_T_TT$GQ))

A_T_TT$AD<-as.character(A_T_TT$AD)

A_T_TT$AD1<-as.numeric(A_T_TT$AD1)

A_T_TT$AD2<-as.numeric(A_T_TT$AD2)

A_T_TT$GQ<-as.numeric(A_T_TT$GQ)

for( i in 1:length(A_T_AT$GQ)){

if(A_T_AT$GQ[i]<=20){A_T_AT$G_GQ[i]<-1}

if(A_T_AT$GQ[i]>20&&A_T_AT$GQ[i]<=40){A_T_AT$G_GQ[i]<-2}

if(A_T_AT$GQ[i]>40&&A_T_AT$GQ[i]<=60){A_T_AT$G_GQ[i]<-3}

if(A_T_AT$GQ[i]>60&&A_T_AT$GQ[i]<=80){A_T_AT$G_GQ[i]<-4}

if(A_T_AT$GQ[i]>80&&A_T_AT$GQ[i]<99){A_T_AT$G_GQ[i]<-5}

if(A_T_AT$GQ[i]>=99){A_T_AT$G_GQ[i]<-6}

}

f_GQ_A_T_AT<-table(A_T_AT$G_GQ)/length(A_T_AT$G_GQ)

for( i in 1:length(A_T_AA$GQ)){

if(A_T_AA$GQ[i]<=20){A_T_AA$G_GQ[i]<-1}

if(A_T_AA$GQ[i]>20&&A_T_AA$GQ[i]<=40){A_T_AA$G_GQ[i]<-2}

if(A_T_AA$GQ[i]>40&&A_T_AA$GQ[i]<=60){A_T_AA$G_GQ[i]<-3}

if(A_T_AA$GQ[i]>60&&A_T_AA$GQ[i]<=80){A_T_AA$G_GQ[i]<-4}

if(A_T_AA$GQ[i]>80&&A_T_AA$GQ[i]<99){A_T_AA$G_GQ[i]<-5}

if(A_T_AA$GQ[i]>=99){A_T_AA$G_GQ[i]<-6}

}

f_GQ_A_T_AA<-table(A_T_AA$G_GQ)/length(A_T_AA$G_GQ)

for( i in 1:length(A_T_TT$GQ)){

if(A_T_TT$GQ[i]<=20){A_T_TT$G_GQ[i]<-1}

if(A_T_TT$GQ[i]>20&&A_T_TT$GQ[i]<=40){A_T_TT$G_GQ[i]<-2}

if(A_T_TT$GQ[i]>40&&A_T_TT$GQ[i]<=60){A_T_TT$G_GQ[i]<-3}

if(A_T_TT$GQ[i]>60&&A_T_TT$GQ[i]<=80){A_T_TT$G_GQ[i]<-4}

if(A_T_TT$GQ[i]>80&&A_T_TT$GQ[i]<99){A_T_TT$G_GQ[i]<-5}

if(A_T_TT$GQ[i]>=99){A_T_TT$G_GQ[i]<-6}

}

f_GQ_A_T_TT<-table(A_T_TT$G_GQ)/length(A_T_TT$G_GQ)

#AG GG AA

#read and modify lists

A_G_AG <- read.table("A_G_AG.txt", header=TRUE, quote="\"")

A_G_GG <- read.table("A_G_GG.txt", header=TRUE, quote="\"")

A_G_AA <- read.table("A_G_AA.txt", header=TRUE, quote="\"")

#make c_T_CC in the correct order

A_G_AA<-data.frame(A_G_AA[,1:4],AD1=A_G_AA$AD1,AD2=A_G_AA$AD2,A_G_AA[,5:7])

#modify AD1 and AD2

A_G_AG<-data.frame(A_G_AG,AD=paste(A_G_AG$AD1,",",A_G_AG$AD2,",",A_G_AG$GQ))

A_G_AG$AD<-as.character(A_G_AG$AD)

A_G_AG$AD1<-as.numeric(A_G_AG$AD1)

A_G_AG$AD2<-as.numeric(A_G_AG$AD2)

A_G_AG$GQ<-as.numeric(A_G_AG$GQ)

A_G_AA<-data.frame(A_G_AA,AD=paste(A_G_AA$AD1,",",A_G_AA$AD2,",",A_G_AA$GQ))

A_G_AA$AD<-as.character(A_G_AA$AD)

A_G_AA$AD1<-as.numeric(A_G_AA$AD1)

A_G_AA$AD2<-as.numeric(A_G_AA$AD2)

A_G_AA$GQ<-as.numeric(A_G_AA$GQ)

A_G_GG<-data.frame(A_G_GG,AD=paste(A_G_GG$AD1,",",A_G_GG$AD2,",",A_G_GG$GQ))

A_G_GG$AD<-as.character(A_G_GG$AD)

A_G_GG$AD1<-as.numeric(A_G_GG$AD1)

A_G_GG$AD2<-as.numeric(A_G_GG$AD2)

A_G_GG$GQ<-as.numeric(A_G_GG$GQ)

for( i in 1:length(A_G_AG$GQ)){

if(A_G_AG$GQ[i]<=20){A_G_AG$G_GQ[i]<-1}

if(A_G_AG$GQ[i]>20&&A_G_AG$GQ[i]<=40){A_G_AG$G_GQ[i]<-2}

if(A_G_AG$GQ[i]>40&&A_G_AG$GQ[i]<=60){A_G_AG$G_GQ[i]<-3}

if(A_G_AG$GQ[i]>60&&A_G_AG$GQ[i]<=80){A_G_AG$G_GQ[i]<-4}

if(A_G_AG$GQ[i]>80&&A_G_AG$GQ[i]<99){A_G_AG$G_GQ[i]<-5}

if(A_G_AG$GQ[i]>=99){A_G_AG$G_GQ[i]<-6}

}

f_GQ_A_G_AG<-table(A_G_AG$G_GQ)/length(A_G_AG$G_GQ)

for( i in 1:length(A_G_AA$GQ)){

if(A_G_AA$GQ[i]<=20){A_G_AA$G_GQ[i]<-1}

if(A_G_AA$GQ[i]>20&&A_G_AA$GQ[i]<=40){A_G_AA$G_GQ[i]<-2}

if(A_G_AA$GQ[i]>40&&A_G_AA$GQ[i]<=60){A_G_AA$G_GQ[i]<-3}

if(A_G_AA$GQ[i]>60&&A_G_AA$GQ[i]<=80){A_G_AA$G_GQ[i]<-4}

if(A_G_AA$GQ[i]>80&&A_G_AA$GQ[i]<99){A_G_AA$G_GQ[i]<-5}

if(A_G_AA$GQ[i]>=99){A_G_AA$G_GQ[i]<-6}

}

f_GQ_A_G_AA<-table(A_G_AA$G_GQ)/length(A_G_AA$G_GQ)

for( i in 1:length(A_G_GG$GQ)){

if(A_G_GG$GQ[i]<=20){A_G_GG$G_GQ[i]<-1}

if(A_G_GG$GQ[i]>20&&A_G_GG$GQ[i]<=40){A_G_GG$G_GQ[i]<-2}

if(A_G_GG$GQ[i]>40&&A_G_GG$GQ[i]<=60){A_G_GG$G_GQ[i]<-3}

if(A_G_GG$GQ[i]>60&&A_G_GG$GQ[i]<=80){A_G_GG$G_GQ[i]<-4}

if(A_G_GG$GQ[i]>80&&A_G_GG$GQ[i]<99){A_G_GG$G_GQ[i]<-5}

if(A_G_GG$GQ[i]>=99){A_G_GG$G_GQ[i]<-6}

}

f_GQ_A_G_GG<-table(A_G_GG$G_GQ)/length(A_G_GG$G_GQ)

#AC AA CC

#read and modify lists

A_C_AC <- read.table("A_C_AC.txt", header=TRUE, quote="\"")

A_C_CC <- read.table("A_C_CC.txt", header=TRUE, quote="\"")

A_C_AA <- read.table("A_C_AA.txt", header=TRUE, quote="\"")

#make c_T_CC in the correct order

A_C_AA<-data.frame(A_C_AA[,1:4],AD1=A_C_AA$AD1,AD2=A_C_AA$AD2,A_C_AA[,5:7])

#modify AD1 and AD2

A_C_AC<-data.frame(A_C_AC,AD=paste(A_C_AC$AD1,",",A_C_AC$AD2,",",A_C_AC$GQ))

A_C_AC$AD<-as.character(A_C_AC$AD)

A_C_AC$AD1<-as.numeric(A_C_AC$AD1)

A_C_AC$AD2<-as.numeric(A_C_AC$AD2)

A_C_AC$GQ<-as.numeric(A_C_AC$GQ)

A_C_AA<-data.frame(A_C_AA,AD=paste(A_C_AA$AD1,",",A_C_AA$AD2,",",A_C_AA$GQ))

A_C_AA$AD<-as.character(A_C_AA$AD)

A_C_AA$AD1<-as.numeric(A_C_AA$AD1)

A_C_AA$AD2<-as.numeric(A_C_AA$AD2)

A_C_AA$GQ<-as.numeric(A_C_AA$GQ)

A_C_CC<-data.frame(A_C_CC,AD=paste(A_C_CC$AD1,",",A_C_CC$AD2,",",A_C_CC$GQ))

A_C_CC$AD<-as.character(A_C_CC$AD)

A_C_CC$AD1<-as.numeric(A_C_CC$AD1)

A_C_CC$AD2<-as.numeric(A_C_CC$AD2)

A_C_CC$GQ<-as.numeric(A_C_CC$GQ)

for( i in 1:length(A_C_AC$GQ)){

if(A_C_AC$GQ[i]<=20){A_C_AC$G_GQ[i]<-1}

if(A_C_AC$GQ[i]>20&&A_C_AC$GQ[i]<=40){A_C_AC$G_GQ[i]<-2}

if(A_C_AC$GQ[i]>40&&A_C_AC$GQ[i]<=60){A_C_AC$G_GQ[i]<-3}

if(A_C_AC$GQ[i]>60&&A_C_AC$GQ[i]<=80){A_C_AC$G_GQ[i]<-4}

if(A_C_AC$GQ[i]>80&&A_C_AC$GQ[i]<99){A_C_AC$G_GQ[i]<-5}

if(A_C_AC$GQ[i]>=99){A_C_AC$G_GQ[i]<-6}

}

f_GQ_A_C_AC<-table(A_C_AC$G_GQ)/length(A_C_AC$G_GQ)

for( i in 1:length(A_C_AA$GQ)){

if(A_C_AA$GQ[i]<=20){A_C_AA$G_GQ[i]<-1}

if(A_C_AA$GQ[i]>20&&A_C_AA$GQ[i]<=40){A_C_AA$G_GQ[i]<-2}

if(A_C_AA$GQ[i]>40&&A_C_AA$GQ[i]<=60){A_C_AA$G_GQ[i]<-3}

if(A_C_AA$GQ[i]>60&&A_C_AA$GQ[i]<=80){A_C_AA$G_GQ[i]<-4}

if(A_C_AA$GQ[i]>80&&A_C_AA$GQ[i]<99){A_C_AA$G_GQ[i]<-5}

if(A_C_AA$GQ[i]>=99){A_C_AA$G_GQ[i]<-6}

}

f_GQ_A_C_AA<-table(A_C_AA$G_GQ)/length(A_C_AA$G_GQ)

for( i in 1:length(A_C_CC$GQ)){

if(A_C_CC$GQ[i]<=20){A_C_CC$G_GQ[i]<-1}

if(A_C_CC$GQ[i]>20&&A_C_CC$GQ[i]<=40){A_C_CC$G_GQ[i]<-2}

if(A_C_CC$GQ[i]>40&&A_C_CC$GQ[i]<=60){A_C_CC$G_GQ[i]<-3}

if(A_C_CC$GQ[i]>60&&A_C_CC$GQ[i]<=80){A_C_CC$G_GQ[i]<-4}

if(A_C_CC$GQ[i]>80&&A_C_CC$GQ[i]<99){A_C_CC$G_GQ[i]<-5}

if(A_C_CC$GQ[i]>=99){A_C_CC$G_GQ[i]<-6}

}

f_GQ_A_C_CC<-table(A_C_CC$G_GQ)/length(A_C_CC$G_GQ)

b<-Sys.time()

print(b-a)

#############################################################################################################################################

######## for every position ############################## ###################################

#############################################################################################################################################

for(p in gt$pos){

print(p)

a<-Sys.time()

gt_pos<-subset(gt,gt$pos==p)

gt_r<-gt_pos[,-(1:4)]

gt_r<-t(gt_r)

#numeric genotypes

GT_q<-gt_r

for(i in 1:length(gt_r)){

if(gt_r[i]=="TT" & gt_pos$alleles=="G/T"){GT_q[i]=2}

if(gt_r[i]=="GT" & gt_pos$alleles=="G/T"){GT_q[i]=1}

if(gt_r[i]=="GG" & gt_pos$alleles=="G/T"){GT_q[i]=0}

if(gt_r[i]=="TT" & gt_pos$alleles=="C/T"){GT_q[i]=2}

if(gt_r[i]=="CT" & gt_pos$alleles=="C/T"){GT_q[i]=1}

if(gt_r[i]=="CC" & gt_pos$alleles=="C/T"){GT_q[i]=0}

if(gt_r[i]=="GG" & gt_pos$alleles=="C/G"){GT_q[i]=2}

if(gt_r[i]=="CG" & gt_pos$alleles=="C/G"){GT_q[i]=1}

if(gt_r[i]=="CC" & gt_pos$alleles=="C/G"){GT_q[i]=0}

if(gt_r[i]=="TT" & gt_pos$alleles=="A/T"){GT_q[i]=2}

if(gt_r[i]=="AT" & gt_pos$alleles=="A/T"){GT_q[i]=1}

if(gt_r[i]=="AA" & gt_pos$alleles=="A/T"){GT_q[i]=0}

if(gt_r[i]=="GG" & gt_pos$alleles=="A/G"){GT_q[i]=2}

if(gt_r[i]=="AG" & gt_pos$alleles=="A/G"){GT_q[i]=1}

if(gt_r[i]=="AA" & gt_pos$alleles=="A/G"){GT_q[i]=0}

if(gt_r[i]=="CC" & gt_pos$alleles=="A/C"){GT_q[i]=2}

if(gt_r[i]=="AC" & gt_pos$alleles=="A/C"){GT_q[i]=1}

if(gt_r[i]=="AA" & gt_pos$alleles=="A/C"){GT_q[i]=0}

}

GT_q1<-as.numeric(GT_q)

names(GT_q1)<-row.names(GT_q)

GT_q<-GT_q1

#phenotypes quantitative

Ph<-GT_q

Phq<-rep(0,length(Ph))

for(i in 1:length(GT_q)){

Phq[i]<-rnorm(1,GT_q[i],6.5)

}

data_1<-data.frame(GT_real=GT_q,Phq)

##########################################################################################################################

#phenotypes categorical

Phd<-GT_q

for(i in 1:length(GT_q)){

if(is.na(Phq[i])==T){Phd[i]<-NA}

if(is.na(Phq[i])==F && Phq[i]<=median(Phq,na.rm=T) ){Phd[i]<-0}

if(is.na(Phq[i])==F && Phq[i]>median(Phq,na.rm=T) ){Phd[i]<-1}

}

#data frame: GT,Ph1,Ph2

data_1<-data.frame(GT_real=GT_q,Phq=Phq,Phd=Phd)

#write.table(data_1,"data_1.txt")

# AD and GQ

ad_sim<-data.frame(AD1=rep(0,1417),AD2=0,GQ=0)

rownames(ad_sim)<-colnames(gt[5:1421])

for (j in 5:length(gt_pos)){

for(i in 1:length(gt_pos$rs)){

if(gt_pos[i,]$alleles == "C/T" & gt_pos[i,j]=="CT"){

#choose gq group

group<-sample(c(1,2,3,4,5,6),1,replace=T,prob=f_GQ_C_T_CT)

#choose a gp randomly for that group

test<-subset(C_T_CT,C_T_CT$G_GQ==group)[,c(5,6,9)]

n<-sample(rownames(test),1)

ad_sim[j-4,1:3]<-test[n,]

}

if(gt_pos[i,]$alleles == "C/T" & gt_pos[i,j]=="TT"){

#choose gq group

group<-sample(c(1,2,3,4,5,6),1,replace=T,prob=f_GQ_C_T_TT)

#choose a gp randomly for that group

test<-subset(C_T_TT,C_T_TT$G_GQ==group)[,c(5,6,9)]

n<-sample(rownames(test),1)

ad_sim[j-4,1:3]<-test[n,]

}

if(gt_pos[i,]$alleles == "C/T" & gt_pos[i,j]=="CC"){

#choose gq group

group<-sample(c(1,2,3,4,5,6),1,replace=T,prob=f_GQ_C_T_CC)

#choose a gp randomly for that group

test<-subset(C_T_CC,C_T_CC$G_GQ==group)[,c(5,6,9)]

n<-sample(rownames(test),1)

ad_sim[j-4,1:3]<-test[n,]

}

if(gt_pos[i,]$alleles == "G/T" & gt_pos[i,j]=="GT"){

#choose gq group

group<-sample(c(1,2,3,4,5,6),1,replace=T,prob=f_GQ_G_T_GT)

#choose a gp randomly for that group

test<-subset(G_T_GT,G_T_GT$G_GQ==group)[,c(5,6,9)]

n<-sample(rownames(test),1)

ad_sim[j-4,1:3]<-test[n,]

}

if(gt_pos[i,]$alleles == "G/T" & gt_pos[i,j]=="TT"){

#choose gq group

group<-sample(c(1,2,3,4,5,6),1,replace=T,prob=f_GQ_G_T_TT)

#choose a gp randomly for that group

test<-subset(G_T_TT,G_T_TT$G_GQ==group)[,c(5,6,9)]

n<-sample(rownames(test),1)

ad_sim[j-4,1:3]<-test[n,]

}

if(gt_pos[i,]$alleles == "G/T" & gt_pos[i,j]=="GG"){

#choose gq group

group<-sample(c(1,2,3,4,5,6),1,replace=T,prob=f_GQ_G_T_GG)

#choose a gp randomly for that group

test<-subset(G_T_GG,G_T_GG$G_GQ==group)[,c(5,6,9)]

n<-sample(rownames(test),1)

ad_sim[j-4,1:3]<-test[n,]

}

if(gt_pos[i,]$alleles == "C/G" & gt_pos[i,j]=="CG"){

#choose gq group

group<-sample(c(1,2,3,4,5,6),1,replace=T,prob=f_GQ_C_G_CG)

#choose a gp randomly for that group

test<-subset(C_G_CG,C_G_CG$G_GQ==group)[,c(5,6,9)]

n<-sample(rownames(test),1)

ad_sim[j-4,1:3]<-test[n,]

}

if(gt_pos[i,]$alleles == "C/G" & gt_pos[i,j]=="GG"){

#choose gq group

group<-sample(c(1,2,3,4,5,6),1,replace=T,prob=f_GQ_C_G_GG)

#choose a gp randomly for that group

test<-subset(C_G_GG,C_G_GG$G_GQ==group)[,c(5,6,9)]

n<-sample(rownames(test),1)

ad_sim[j-4,1:3]<-test[n,]

}

if(gt_pos[i,]$alleles == "C/G" & gt_pos[i,j]=="CC"){

#choose gq group

group<-sample(c(1,2,3,4,5,6),1,replace=T,prob=f_GQ_C_G_CC)

#choose a gp randomly for that group

test<-subset(C_G_CC,C_G_CC$G_GQ==group)[,c(5,6,9)]

n<-sample(rownames(test),1)

ad_sim[j-4,1:3]<-test[n,]

}

if(gt_pos[i,]$alleles == "A/T" & gt_pos[i,j]=="AT"){

#choose gq group

group<-sample(c(1,2,3,4,5,6),1,replace=T,prob=f_GQ_A_T_AT)

#choose a gp randomly for that group

test<-subset(A_T_AT,A_T_AT$G_GQ==group)[,c(5,6,9)]

n<-sample(rownames(test),1)

ad_sim[j-4,1:3]<-test[n,]

}

if(gt_pos[i,]$alleles == "A/T" & gt_pos[i,j]=="TT"){

#choose gq group

group<-sample(c(1,2,3,4,5,6),1,replace=T,prob=f_GQ_A_T_TT)

#choose a gp randomly for that group

test<-subset(A_T_TT,A_T_TT$G_GQ==group)[,c(5,6,9)]

n<-sample(rownames(test),1)

ad_sim[j-4,1:3]<-test[n,]

}

if(gt_pos[i,]$alleles == "A/T" & gt_pos[i,j]=="AA"){

#choose gq group

group<-sample(c(1,2,3,4,5,6),1,replace=T,prob=f_GQ_A_T_AA)

#choose a gp randomly for that group

test<-subset(A_T_AA,A_T_AA$G_GQ==group)[,c(5,6,9)]

n<-sample(rownames(test),1)

ad_sim[j-4,1:3]<-test[n,]

}

if(gt_pos[i,]$alleles == "A/G" & gt_pos[i,j]=="AG"){

#choose gq group

group<-sample(c(1,2,3,4,5,6),1,replace=T,prob=f_GQ_A_G_AG)

#choose a gp randomly for that group

test<-subset(A_G_AG,A_G_AG$G_GQ==group)[,c(5,6,9)]

n<-sample(rownames(test),1)

ad_sim[j-4,1:3]<-test[n,]

}

if(gt_pos[i,]$alleles == "A/G" & gt_pos[i,j]=="GG"){

#choose gq group

group<-sample(c(1,2,3,4,5,6),1,replace=T,prob=f_GQ_A_G_GG)

#choose a gp randomly for that group

test<-subset(A_G_GG,A_G_GG$G_GQ==group)[,c(5,6,9)]

n<-sample(rownames(test),1)

ad_sim[j-4,1:3]<-test[n,]

}

if(gt_pos[i,]$alleles == "A/G" & gt_pos[i,j]=="AA"){

#choose gq group

group<-sample(c(1,2,3,4,5,6),1,replace=T,prob=f_GQ_A_G_AA)

#choose a gp randomly for that group

test<-subset(A_G_AA,A_G_AA$G_GQ==group)[,c(5,6,9)]

n<-sample(rownames(test),1)

ad_sim[j-4,1:3]<-test[n,]

}

if(gt_pos[i,]$alleles == "A/C" & gt_pos[i,j]=="AC"){

#choose gq group

group<-sample(c(1,2,3,4,5,6),1,replace=T,prob=f_GQ_A_C_AC)

#choose a gp randomly for that group

test<-subset(A_C_AC,A_C_AC$G_GQ==group)[,c(5,6,9)]

n<-sample(rownames(test),1)

ad_sim[j-4,1:3]<-test[n,]

}

if(gt_pos[i,]$alleles == "A/C" & gt_pos[i,j]=="CC"){

#choose gq group

group<-sample(c(1,2,3,4,5,6),1,replace=T,prob=f_GQ_A_C_CC)

#choose a gp randomly for that group

test<-subset(A_C_CC,A_C_CC$G_GQ==group)[,c(5,6,9)]

n<-sample(rownames(test),1)

ad_sim[j-4,1:3]<-test[n,]

}

if(gt_pos[i,]$alleles == "A/C" & gt_pos[i,j]=="AA"){

#choose gq group

group<-sample(c(1,2,3,4,5,6),1,replace=T,prob=f_GQ_A_C_AA)

#choose a gp randomly for that group

test<-subset(A_C_AA,A_C_AA$G_GQ==group)[,c(5,6,9)]

n<-sample(rownames(test),1)

ad_sim[j-4,1:3]<-test[n,]

}

}}

# ad[v]<-ad_sim

# print(k)

#} #fin k --- sim GQ AD1 AD2

data_2<-data.frame(GT_real=GT_q,ad_sim)

#names_ad<-paste(c("AD1","AD2","GQ"),rep(1:100, each=3),sep="")

#colnames(data_2)<-c("GT_real",names_ad)

#simulate 100 data set mixed

data_3<-data.frame(GT_real=data_1$GT_real,Phq=data_1$Phq,Phd=data_1$Phd,GQ=data_2$GQ,AD1=data_2$AD1,AD2=data_2$AD2,GT_estim=estim_gt(data_2[,-1])$GT)

data_3$Phq<-as.numeric(data_3$Phq)

data<-data.frame(data_3,S_AD=data_3$AD1+data_3$AD2)

#remove individual with 0,0 AD

data<-subset(data, data$S_AD!=0)

#GT in factor and group

for( i in 1:length(data$GT_estim)){

if(data$GT_estim[i]=="0/0"){data$GT_estim_g[i]<-0}

if(data$GT_estim[i]=="0/1"){data$GT_estim_g[i]<-1}

if(data$GT_estim[i]=="1/1"){data$GT_estim_g[i]<-2}

}

data$GT_estim_g<-as.factor(data$GT_estim_g)

data$GT_estim<-as.factor(data$GT_estim)

data$GT_real<-as.factor(data$GT_real)

data<-data.frame(data,ratio=data$AD2/data$S_AD)

data<-cbind(data,GT_realn=as.numeric(as.character(data$GT_real)))

data<-cbind(data,GT_estim_gn=as.numeric(as.character(data$GT_estim_g)))

# write.table(data_3,"data_3.txt")

#save contingency table

r0e0<-length(subset(data,data$GT_real==0 & data$GT_estim_g==0)[,1])

r0e1<-length(subset(data,data$GT_real==0 & data$GT_estim_g==1)[,1])

r0e2<-length(subset(data,data$GT_real==0 & data$GT_estim_g==2)[,1])

r1e0<-length(subset(data,data$GT_real==1 & data$GT_estim_g==0)[,1])

r1e1<-length(subset(data,data$GT_real==1 & data$GT_estim_g==1)[,1])

r1e2<-length(subset(data,data$GT_real==1 & data$GT_estim_g==2)[,1])

r2e0<-length(subset(data,data$GT_real==2 & data$GT_estim_g==0)[,1])

r2e1<-length(subset(data,data$GT_real==2 & data$GT_estim_g==1)[,1])

r2e2<-length(subset(data,data$GT_real==2 & data$GT_estim_g==2)[,1])

#save

medianGQ<-median(data$GQ)

medianSAD<-median(data$S_AD)

print("fitting models")

#######################################################################################################################

#################################### AD2~Phq ###############################################################

#######################################################################################################################

#standard poisson model AD2~Phq

cc <- try(glm(AD2~Phq, family=poisson,data=data, offset=log(S_AD)), silent=T)

if(is(cc,"try-error")) {ad2_ph_poisson<-NA

e_ad2_ph_poisson_beta0<-NA

e_ad2_ph_poisson_beta0_se<-NA

e_ad2_ph_poisson_beta1<-NA

e_ad2_ph_poisson_beta1_se<-NA}

if(is(cc,"try-error")==F) {ad2_ph_poisson<-summary(glm(AD2~Phq, family=poisson,data=data, offset=log(S_AD)))$coefficients[2,4]

e_ad2_ph_poisson_beta0<-summary(glm(AD2~Phq, family=poisson,data=data, offset=log(S_AD)))$coefficients[1,1]

e_ad2_ph_poisson_beta0_se<-summary(glm(AD2~Phq, family=poisson,data=data, offset=log(S_AD)))$coefficients[1,2]

e_ad2_ph_poisson_beta1<-summary(glm(AD2~Phq, family=poisson,data=data, offset=log(S_AD)))$coefficients[2,1]

e_ad2_ph_poisson_beta1_se<-summary(glm(AD2~Phq, family=poisson,data=data, offset=log(S_AD)))$coefficients[2,2]}

#negative binomial AD2~Phq

cc <- try(glm.nb(AD2~Phq+offset(log(S_AD)), data = data), silent=T)

if(is(cc,"try-error")) { ad2_ph_nb<-NA

e_ad2_ph_nb_beta0<-NA

e_ad2_ph_nb_beta0_se<-NA

e_ad2_ph_nb_beta1<-NA

e_ad2_ph_nb_beta1_se<-NA}

if(is(cc,"try-error")==F) { ad2_ph_nb<-summary(glm.nb(AD2~Phq+offset(log(S_AD)), data = data))$coefficients[2,4]

e_ad2_ph_nb_beta0<-summary(glm.nb(AD2~Phq+offset(log(S_AD)), data = data))$coefficients[1,1]

e_ad2_ph_nb_beta0_se<-summary(glm.nb(AD2~Phq+offset(log(S_AD)), data = data))$coefficients[1,2]

e_ad2_ph_nb_beta1<-summary(glm.nb(AD2~Phq+offset(log(S_AD)), data = data))$coefficients[2,1]

e_ad2_ph_nb_beta1_se<-summary(glm.nb(AD2~Phq+offset(log(S_AD)), data = data))$coefficients[2,2]}

#zero inflated negative binomial AD2~Phq

cc <- try(zeroinfl(AD2~Phq,data=data,offset=log(S_AD),dist = "negbin"), silent=T)

xx <- try(zeroinfl(AD2~1, data=data,offset=log(S_AD),dist = "negbin"),silent=T)

if(is(cc,"try-error")) {ad2_ph_nb_zi<-NA

e_ad2_ph_nb_zi_beta0c<-NA

e_ad2_ph_nb_zi_beta0c_se<-NA

e_ad2_ph_nb_zi_beta1c<-NA

e_ad2_ph_nb_zi_beta1c_se<-NA

e_ad2_ph_nb_zi_logthetac<-NA

e_ad2_ph_nb_zi_logthetac_se<-NA

e_ad2_ph_nb_zi_beta0z<-NA

e_ad2_ph_nb_zi_beta0z_se<-NA

e_ad2_ph_nb_zi_beta1z<-NA

e_ad2_ph_nb_zi_beta1z_se<-NA}

if(is(xx,"try-error")) {ad2_ph_nb_zi<-NA

e_ad2_ph_nb_zi_beta0c<-NA

e_ad2_ph_nb_zi_beta0c_se<-NA

e_ad2_ph_nb_zi_beta1c<-NA

e_ad2_ph_nb_zi_beta1c_se<-NA

e_ad2_ph_nb_zi_logthetac<-NA

e_ad2_ph_nb_zi_logthetac_se<-NA

e_ad2_ph_nb_zi_beta0z<-NA

e_ad2_ph_nb_zi_beta0z_se<-NA

e_ad2_ph_nb_zi_beta1z<-NA

e_ad2_ph_nb_zi_beta1z_se<-NA}

if(is(cc,"try-error")==F & is(xx,"try-error")==F) { ad2_ph_nb_zi<-1-pchisq(-2*(zeroinfl(AD2~1,data=data,offset=log(S_AD),dist = "negbin")$loglik-zeroinfl(AD2~Phq,data=data,offset=log(S_AD),dist = "negbin")$loglik),2)

e_ad2_ph_nb_zi_beta0c<-summary(zeroinfl(AD2~Phq,data=data,offset=log(S_AD),dist = "negbin"))$coefficients$count[1,1]

e_ad2_ph_nb_zi_beta0c_se<-summary(zeroinfl(AD2~Phq,data=data,offset=log(S_AD),dist = "negbin"))$coefficients$count[1,2]

e_ad2_ph_nb_zi_beta1c<-summary(zeroinfl(AD2~Phq,data=data,offset=log(S_AD),dist = "negbin"))$coefficients$count[2,1]

e_ad2_ph_nb_zi_beta1c_se<-summary(zeroinfl(AD2~Phq,data=data,offset=log(S_AD),dist = "negbin"))$coefficients$count[2,2]

e_ad2_ph_nb_zi_logthetac<-summary(zeroinfl(AD2~Phq,data=data,offset=log(S_AD),dist = "negbin"))$coefficients$count[3,1]

e_ad2_ph_nb_zi_logthetac_se<-summary(zeroinfl(AD2~Phq,data=data,offset=log(S_AD),dist = "negbin"))$coefficients$count[3,2]

e_ad2_ph_nb_zi_beta0z<-summary(zeroinfl(AD2~Phq,data=data,offset=log(S_AD),dist = "negbin"))$coefficients$zero[1,1]

e_ad2_ph_nb_zi_beta0z_se<-summary(zeroinfl(AD2~Phq,data=data,offset=log(S_AD),dist = "negbin"))$coefficients$zero[1,2]

e_ad2_ph_nb_zi_beta1z<-summary(zeroinfl(AD2~Phq,data=data,offset=log(S_AD),dist = "negbin"))$coefficients$zero[2,1]

e_ad2_ph_nb_zi_beta1z_se<-summary(zeroinfl(AD2~Phq,data=data,offset=log(S_AD),dist = "negbin"))$coefficients$zero[2,2]}

#hurdle negative binomial AD2~Phq

cc <- try(hurdle(AD2~Phq, data=data,offset=log(S_AD),dist = "negbin"), silent=T)

xx <- try(hurdle(AD2~1, data=data,offset=log(S_AD),dist = "negbin"),silent=T)

if(is(cc,"try-error")) {ad2_ph_nb_hur<-NA

e_ad2_ph_nb_hur_beta0c<-NA

e_ad2_ph_nb_hur_beta0c_se<-NA

e_ad2_ph_nb_hur_beta1c<-NA

e_ad2_ph_nb_hur_beta1c_se<-NA

e_ad2_ph_nb_hur_logthetac<-NA

e_ad2_ph_nb_hur_logthetac_se<-NA

e_ad2_ph_nb_hur_beta0z<-NA

e_ad2_ph_nb_hur_beta0z_se<-NA

e_ad2_ph_nb_hur_beta1z<-NA

e_ad2_ph_nb_hur_beta1z_se<-NA }

if(is(xx,"try-error")) {ad2_ph_nb_hur<-NA

e_ad2_ph_nb_hur_beta0c<-NA

e_ad2_ph_nb_hur_beta0c_se<-NA

e_ad2_ph_nb_hur_beta1c<-NA

e_ad2_ph_nb_hur_beta1c_se<-NA

e_ad2_ph_nb_hur_logthetac<-NA

e_ad2_ph_nb_hur_logthetac_se<-NA

e_ad2_ph_nb_hur_beta0z<-NA

e_ad2_ph_nb_hur_beta0z_se<-NA

e_ad2_ph_nb_hur_beta1z<-NA

e_ad2_ph_nb_hur_beta1z_se<-NA }

if(is(cc,"try-error")==F & is(xx,"try-error")==F) { ad2_ph_nb_hur<-1-pchisq(-2*(hurdle(AD2~1, data=data,offset=log(S_AD),dist = "negbin")$loglik-hurdle(AD2~Phq, data=data,offset=log(S_AD),dist = "negbin")$loglik),2)

e_ad2_ph_nb_hur_beta0c<-summary(hurdle(AD2~Phq, data=data,offset=log(S_AD),dist = "negbin"))$coefficients$count[1,1]

e_ad2_ph_nb_hur_beta0c_se<-summary(hurdle(AD2~Phq, data=data,offset=log(S_AD),dist = "negbin"))$coefficients$count[1,2]

e_ad2_ph_nb_hur_beta1c<-summary(hurdle(AD2~Phq, data=data,offset=log(S_AD),dist = "negbin"))$coefficients$count[2,1]

e_ad2_ph_nb_hur_beta1c_se<-summary(hurdle(AD2~Phq, data=data,offset=log(S_AD),dist = "negbin"))$coefficients$count[2,2]

e_ad2_ph_nb_hur_logthetac<-summary(hurdle(AD2~Phq, data=data,offset=log(S_AD),dist = "negbin"))$coefficients$count[3,1]

e_ad2_ph_nb_hur_logthetac_se<-summary(hurdle(AD2~Phq, data=data,offset=log(S_AD),dist = "negbin"))$coefficients$count[3,2]

e_ad2_ph_nb_hur_beta0z<-summary(hurdle(AD2~Phq, data=data,offset=log(S_AD),dist = "negbin"))$coefficients$zero[1,1]

e_ad2_ph_nb_hur_beta0z_se<-summary(hurdle(AD2~Phq, data=data,offset=log(S_AD),dist = "negbin"))$coefficients$zero[1,2]

e_ad2_ph_nb_hur_beta1z<-summary(hurdle(AD2~Phq, data=data,offset=log(S_AD),dist = "negbin"))$coefficients$zero[2,1]

e_ad2_ph_nb_hur_beta1z_se<-summary(hurdle(AD2~Phq, data=data,offset=log(S_AD),dist = "negbin"))$coefficients$zero[2,2] }

#######################################################################################################################

#################################### ratio~Phq ###############################################################

#######################################################################################################################

#standard linear model Phq~AD2

cc <- try(lm(ratio~Phq, data = data), silent=T)

xx<- try(summary(lm(ratio~Phq, data = data)), silent=T)

if(is(cc,"try-error")) { ratio_ph_lm<-NA

e_ratio_ph_lm_beta0<-NA

e_ratio_ph_lm_beta0_se<-NA

e_ratio_ph_lm_beta1<-NA

e_ratio_ph_lm_beta1_se<-NA}

if(is(xx,"try-error")) { ratio_ph_lm<-NA

e_ratio_ph_lm_beta0<-NA

e_ratio_ph_lm_beta0_se<-NA

e_ratio_ph_lm_beta1<-NA

e_ratio_ph_lm_beta1_se<-NA}

if(is(cc,"try-error")==F & is(xx,"try-error")==F) { ratio_ph_lm<-summary(lm(ratio~Phq, data = data))$coefficients[2,4]

e_ratio_ph_lm_beta0<-summary(lm(ratio~Phq, data = data))$coefficients[1,1]

e_ratio_ph_lm_beta0_se<-summary(lm(ratio~Phq, data = data))$coefficients[1,2]

e_ratio_ph_lm_beta1<-summary(lm(ratio~Phq, data = data))$coefficients[2,1]

e_ratio_ph_lm_beta1_se<-summary(lm(ratio~Phq, data = data))$coefficients[2,2]}

#######################################################################################################################

#################################### Phq~ratio ###############################################################

#######################################################################################################################

#standard linear model Phq~AD2

cc <- try(lm(Phq~ratio, data = data), silent=T)

xx<- try(summary(lm(Phq~ratio, data = data)), silent=T)

if(is(cc,"try-error")) { ph_ratio_lm<-NA

e_ph_ratio_lm_beta0<-NA

e_ph_ratio_lm_beta0_se<-NA

e_ph_ratio_lm_beta1<-NA

e_ph_ratio_lm_beta1_se<-NA}

if(is(xx,"try-error")) { ph_ratio_lm<-NA

e_ph_ratio_lm_beta0<-NA

e_ph_ratio_lm_beta0_se<-NA

e_ph_ratio_lm_beta1<-NA

e_ph_ratio_lm_beta1_se<-NA}

if(is(cc,"try-error")==F & is(xx,"try-error")==F) { ph_ratio_lm<-summary(lm(Phq~ratio, data = data))$coefficients[2,4]

e_ph_ratio_lm_beta0<-summary(lm(Phq~ratio, data = data))$coefficients[1,1]

e_ph_ratio_lm_beta0_se<-summary(lm(Phq~ratio, data = data))$coefficients[1,2]

e_ph_ratio_lm_beta1<-summary(lm(Phq~ratio, data = data))$coefficients[2,1]

e_ph_ratio_lm_beta1_se<-summary(lm(Phq~ratio, data = data))$coefficients[2,2]}

#######################################################################################################################

#################################### GT_estim~Phq ############################################################

#######################################################################################################################

#ordinal logistic standard model GT_estim~Phq

cc <- try(polr(GT_estim~Phq, data = data, Hess = TRUE), silent=T)

if(is(cc,"try-error")) {gt_ph_ordinal_logit<-NA

e_gt_ph_ordinal_logit_coef<-NA

e_gt_ph_ordinal_logit_coef_se<-NA

e_gt_ph_ordinal_logit_int1<-NA

e_gt_ph_ordinal_logit_int1_se<-NA

e_gt_ph_ordinal_logit_int2<-NA

e_gt_ph_ordinal_logit_int2_se<-NA}

if(is(cc,"try-error")==F) {gt_ph_ordinal_logit<-pnorm(abs(summary( polr(GT_estim~Phq, data = data, Hess = TRUE) )$coefficients[1,3]),lower.tail=F)*2

e_gt_ph_ordinal_logit_coef<-summary( polr(GT_estim~Phq, data = data, Hess = TRUE) )$coefficients[1,1]

e_gt_ph_ordinal_logit_coef_se<-summary( polr(GT_estim~Phq, data = data, Hess = TRUE) )$coefficients[1,2]

e_gt_ph_ordinal_logit_int1<-summary( polr(GT_estim~Phq, data = data, Hess = TRUE) )$coefficients[2,1]

e_gt_ph_ordinal_logit_int1_se<-summary( polr(GT_estim~Phq, data = data, Hess = TRUE) )$coefficients[2,2]

e_gt_ph_ordinal_logit_int2<-summary( polr(GT_estim~Phq, data = data, Hess = TRUE) )$coefficients[3,1]

e_gt_ph_ordinal_logit_int2_se<-summary( polr(GT_estim~Phq, data = data, Hess = TRUE) )$coefficients[3,2]}

#logistic regression standard GT_estim_g~Phq

cc <- try(glm(GT_estim_g~Phq, family=binomial(link=logit),data=data), silent=T)

xx<- try(summary(glm(GT_estim_g~Phq, family=binomial(link=logit),data=data), silent=T))

if(is(cc,"try-error")) { gt_ph_logit<-NA

e_gt_ph_logit_beta0<-NA

e_gt_ph_logit_beta0_se<-NA

e_gt_ph_logit_beta1<-NA

e_gt_ph_logit_beta1_se<-NA}

if(is(xx,"try-error")) { gt_ph_logit<-NA

e_gt_ph_logit_beta0<-NA

e_gt_ph_logit_beta0_se<-NA

e_gt_ph_logit_beta1<-NA

e_gt_ph_logit_beta1_se<-NA}

if(is(cc,"try-error")==F & is(xx,"try-error")==F) { gt_ph_logit<-summary(glm(GT_estim_g~Phq, family=binomial(link=logit),data=data))$coefficients[2,4]

e_gt_ph_logit_beta0<-summary(glm(GT_estim_g~Phq, family=binomial(link=logit),data=data))$coefficients[1,1]

e_gt_ph_logit_beta0_se<-summary(glm(GT_estim_g~Phq, family=binomial(link=logit),data=data))$coefficients[1,2]

e_gt_ph_logit_beta1<-summary(glm(GT_estim_g~Phq, family=binomial(link=logit),data=data))$coefficients[2,1]

e_gt_ph_logit_beta1_se<-summary(glm(GT_estim_g~Phq, family=binomial(link=logit),data=data))$coefficients[2,2]}

#######################################################################################################################

#################################### Phq~GT_estim ###########################################################

#######################################################################################################################

#standard linear model Phq~GT_estim_gn

cc <- try(lm(Phq~GT_estim_gn , data = data), silent=T)

xx<- try(summary(lm(Phq~GT_estim_gn , data = data)), silent=T)

if(is(cc,"try-error")) { ph_gt_lm<-NA

e_ph_gt_lm_beta0<-NA

e_ph_gt_lm_beta0_se<-NA

e_ph_gt_lm_beta1<-NA

e_ph_gt_lm_beta1_se<-NA}

if(is(xx,"try-error")) { ph_gt_lm<-NA

e_ph_gt_lm_beta0<-NA

e_ph_gt_lm_beta0_se<-NA

e_ph_gt_lm_beta1<-NA

e_ph_gt_lm_beta1_se<-NA}

if(is(cc,"try-error")==F & is(xx,"try-error")==F) { ph_gt_lm<-summary(lm(Phq~GT_estim_gn , data = data))$coefficients[2,4]

e_ph_gt_lm_beta0<-summary(lm(Phq~GT_estim_gn , data = data))$coefficients[1,1]

e_ph_gt_lm_beta0_se<-summary(lm(Phq~GT_estim_gn , data = data))$coefficients[1,2]

e_ph_gt_lm_beta1<-summary(lm(Phq~GT_estim_gn , data = data))$coefficients[2,1]

e_ph_gt_lm_beta1_se<-summary(lm(Phq~GT_estim_gn , data = data))$coefficients[2,2]}

#######################################################################################################################

#################################### GT_real~Phq ############################################################

#######################################################################################################################

#ordinal logistic standard model GT_real~Phq

cc <- try(polr(GT_real~Phq, data = data, Hess = TRUE), silent=T)

if(is(cc,"try-error")) {gtr_ph_ordinal_logit<-NA

e_gtr_ph_ordinal_logit_coef<-NA

e_gtr_ph_ordinal_logit_coef_se<-NA

e_gtr_ph_ordinal_logit_int1<-NA

e_gtr_ph_ordinal_logit_int1_se<-NA

e_gtr_ph_ordinal_logit_int2<-NA

e_gtr_ph_ordinal_logit_int2_se<-NA}

if(is(cc,"try-error")==F) {gtr_ph_ordinal_logit<-pnorm(abs(summary( polr(GT_real~Phq, data = data, Hess = TRUE) )$coefficients[1,3]),lower.tail=F)*2

e_gtr_ph_ordinal_logit_coef<-summary( polr(GT_real~Phq, data = data, Hess = TRUE) )$coefficients[1,1]

e_gtr_ph_ordinal_logit_coef_se<-summary( polr(GT_real~Phq, data = data, Hess = TRUE) )$coefficients[1,2]

e_gtr_ph_ordinal_logit_int1<-summary( polr(GT_real~Phq, data = data, Hess = TRUE) )$coefficients[2,1]

e_gtr_ph_ordinal_logit_int1_se<-summary( polr(GT_real~Phq, data = data, Hess = TRUE) )$coefficients[2,2]

e_gtr_ph_ordinal_logit_int2<-summary( polr(GT_real~Phq, data = data, Hess = TRUE) )$coefficients[3,1]

e_gtr_ph_ordinal_logit_int2_se<-summary( polr(GT_real~Phq, data = data, Hess = TRUE) )$coefficients[3,2]}

#logistic regression standard GT_real~Phq

cc <- try(glm(GT_real~Phq, family=binomial(link=logit),data=data), silent=T)

xx<- try(summary(glm(GT_real~Phq, family=binomial(link=logit),data=data), silent=T))

if(is(cc,"try-error")) { gtr_ph_logit<-NA

e_gtr_ph_logit_beta0<-NA

e_gtr_ph_logit_beta0_se<-NA

e_gtr_ph_logit_beta1<-NA

e_gtr_ph_logit_beta1_se<-NA}

if(is(xx,"try-error")) { gtr_ph_logit<-NA

e_gtr_ph_logit_beta0<-NA

e_gtr_ph_logit_beta0_se<-NA

e_gtr_ph_logit_beta1<-NA

e_gtr_ph_logit_beta1_se<-NA}

if(is(cc,"try-error")==F & is(xx,"try-error")==F) { gtr_ph_logit<-summary(glm(GT_real~Phq, family=binomial(link=logit),data=data))$coefficients[2,4]

e_gtr_ph_logit_beta0<-summary(glm(GT_real~Phq, family=binomial(link=logit),data=data))$coefficients[1,1]

e_gtr_ph_logit_beta0_se<-summary(glm(GT_real~Phq, family=binomial(link=logit),data=data))$coefficients[1,2]

e_gtr_ph_logit_beta1<-summary(glm(GT_real~Phq, family=binomial(link=logit),data=data))$coefficients[2,1]

e_gtr_ph_logit_beta1_se<-summary(glm(GT_real~Phq, family=binomial(link=logit),data=data))$coefficients[2,2]}

#######################################################################################################################

#################################### Phq~GT_real ###########################################################

#######################################################################################################################

#standard linear model Phq~GT_realn

cc <- try(lm(Phq~GT_realn , data = data), silent=T)

xx<- try(summary(lm(Phq~GT_realn , data = data)), silent=T)

if(is(cc,"try-error")) { ph_gtr_lm<-NA

e_ph_gtr_lm_beta0<-NA

e_ph_gtr_lm_beta0_se<-NA

e_ph_gtr_lm_beta1<-NA

e_ph_gtr_lm_beta1_se<-NA}

if(is(xx,"try-error")) { ph_gtr_lm<-NA

e_ph_gtr_lm_beta0<-NA

e_ph_gtr_lm_beta0_se<-NA

e_ph_gtr_lm_beta1<-NA

e_ph_gtr_lm_beta1_se<-NA}

if(is(cc,"try-error")==F & is(xx,"try-error")==F) { ph_gtr_lm<-summary(lm(Phq~GT_realn , data = data))$coefficients[2,4]

e_ph_gtr_lm_beta0<-summary(lm(Phq~GT_realn , data = data))$coefficients[1,1]

e_ph_gtr_lm_beta0_se<-summary(lm(Phq~GT_realn , data = data))$coefficients[1,2]

e_ph_gtr_lm_beta1<-summary(lm(Phq~GT_realn , data = data))$coefficients[2,1]

e_ph_gtr_lm_beta1_se<-summary(lm(Phq~GT_realn , data = data))$coefficients[2,2]}

##############################################################################################################

###################################### PHENOTYPE CATEGORICAL ######################################

##############################################################################################################

#################################### AD2~Phd ###########################################################

#standard poisson model AD2~Phd

cc <- try(glm(AD2~Phd, family=poisson,data=data, offset=log(S_AD)), silent=T)

if(is(cc,"try-error")) {ad2_phd_poisson<-NA

e_ad2_phd_poisson_beta0<-NA

e_ad2_phd_poisson_beta0_se<-NA

e_ad2_phd_poisson_beta1<-NA

e_ad2_phd_poisson_beta1_se<-NA}

if(is(cc,"try-error")==F) {ad2_phd_poisson<-summary(glm(AD2~Phd, family=poisson,data=data, offset=log(S_AD)))$coefficients[2,4]

e_ad2_phd_poisson_beta0<-summary(glm(AD2~Phd, family=poisson,data=data, offset=log(S_AD)))$coefficients[1,1]

e_ad2_phd_poisson_beta0_se<-summary(glm(AD2~Phd, family=poisson,data=data, offset=log(S_AD)))$coefficients[1,2]

e_ad2_phd_poisson_beta1<-summary(glm(AD2~Phd, family=poisson,data=data, offset=log(S_AD)))$coefficients[2,1]

e_ad2_phd_poisson_beta1_se<-summary(glm(AD2~Phd, family=poisson,data=data, offset=log(S_AD)))$coefficients[2,2]}

#negative binomial AD2~Phd

cc <- try(glm.nb(AD2~Phd+offset(log(S_AD)), data = data), silent=T)

if(is(cc,"try-error")) { ad2_phd_nb<-NA

e_ad2_phd_nb_beta0<-NA

e_ad2_phd_nb_beta0_se<-NA

e_ad2_phd_nb_beta1<-NA

e_ad2_phd_nb_beta1_se<-NA}

if(is(cc,"try-error")==F) { ad2_phd_nb<-summary(glm.nb(AD2~Phd+offset(log(S_AD)), data = data))$coefficients[2,4]

e_ad2_phd_nb_beta0<-summary(glm.nb(AD2~Phd+offset(log(S_AD)), data = data))$coefficients[1,1]

e_ad2_phd_nb_beta0_se<-summary(glm.nb(AD2~Phd+offset(log(S_AD)), data = data))$coefficients[1,2]

e_ad2_phd_nb_beta1<-summary(glm.nb(AD2~Phd+offset(log(S_AD)), data = data))$coefficients[2,1]

e_ad2_phd_nb_beta1_se<-summary(glm.nb(AD2~Phd+offset(log(S_AD)), data = data))$coefficients[2,2]}

#zero inflated negative binomial AD2~Phd

cc <- try(zeroinfl(AD2~Phd,data=data,offset=log(S_AD),dist = "negbin"), silent=T)

xx <- try(zeroinfl(AD2~1, data=data,offset=log(S_AD),dist = "negbin"),silent=T)

if(is(cc,"try-error")) {ad2_phd_nb_zi<-NA

e_ad2_phd_nb_zi_beta0c<-NA

e_ad2_phd_nb_zi_beta0c_se<-NA

e_ad2_phd_nb_zi_beta1c<-NA

e_ad2_phd_nb_zi_beta1c_se<-NA

e_ad2_phd_nb_zi_logthetac<-NA

e_ad2_phd_nb_zi_logthetac_se<-NA

e_ad2_phd_nb_zi_beta0z<-NA

e_ad2_phd_nb_zi_beta0z_se<-NA

e_ad2_phd_nb_zi_beta1z<-NA

e_ad2_phd_nb_zi_beta1z_se<-NA}

if(is(xx,"try-error")) {ad2_phd_nb_zi<-NA

e_ad2_phd_nb_zi_beta0c<-NA

e_ad2_phd_nb_zi_beta0c_se<-NA

e_ad2_phd_nb_zi_beta1c<-NA

e_ad2_phd_nb_zi_beta1c_se<-NA

e_ad2_phd_nb_zi_logthetac<-NA

e_ad2_phd_nb_zi_logthetac_se<-NA

e_ad2_phd_nb_zi_beta0z<-NA

e_ad2_phd_nb_zi_beta0z_se<-NA

e_ad2_phd_nb_zi_beta1z<-NA

e_ad2_phd_nb_zi_beta1z_se<-NA}

if(is(cc,"try-error")==F & is(xx,"try-error")==F) { ad2_phd_nb_zi<-1-pchisq(-2*(zeroinfl(AD2~1,data=data,offset=log(S_AD),dist = "negbin")$loglik-zeroinfl(AD2~Phd,data=data,offset=log(S_AD),dist = "negbin")$loglik),2)

e_ad2_phd_nb_zi_beta0c<-summary(zeroinfl(AD2~Phd,data=data,offset=log(S_AD),dist = "negbin"))$coefficients$count[1,1]

e_ad2_phd_nb_zi_beta0c_se<-summary(zeroinfl(AD2~Phd,data=data,offset=log(S_AD),dist = "negbin"))$coefficients$count[1,2]

e_ad2_phd_nb_zi_beta1c<-summary(zeroinfl(AD2~Phd,data=data,offset=log(S_AD),dist = "negbin"))$coefficients$count[2,1]

e_ad2_phd_nb_zi_beta1c_se<-summary(zeroinfl(AD2~Phd,data=data,offset=log(S_AD),dist = "negbin"))$coefficients$count[2,2]

e_ad2_phd_nb_zi_logthetac<-summary(zeroinfl(AD2~Phd,data=data,offset=log(S_AD),dist = "negbin"))$coefficients$count[3,1]

e_ad2_phd_nb_zi_logthetac_se<-summary(zeroinfl(AD2~Phd,data=data,offset=log(S_AD),dist = "negbin"))$coefficients$count[3,2]

e_ad2_phd_nb_zi_beta0z<-summary(zeroinfl(AD2~Phd,data=data,offset=log(S_AD),dist = "negbin"))$coefficients$zero[1,1]

e_ad2_phd_nb_zi_beta0z_se<-summary(zeroinfl(AD2~Phd,data=data,offset=log(S_AD),dist = "negbin"))$coefficients$zero[1,2]

e_ad2_phd_nb_zi_beta1z<-summary(zeroinfl(AD2~Phd,data=data,offset=log(S_AD),dist = "negbin"))$coefficients$zero[2,1]

e_ad2_phd_nb_zi_beta1z_se<-summary(zeroinfl(AD2~Phd,data=data,offset=log(S_AD),dist = "negbin"))$coefficients$zero[2,2]}

#hurdle negative binomial AD2~Phd

cc <- try(hurdle(AD2~Phd, data=data,offset=log(S_AD),dist = "negbin"), silent=T)

xx <- try(hurdle(AD2~1, data=data,offset=log(S_AD),dist = "negbin"),silent=T)

if(is(cc,"try-error")) {ad2_phd_nb_hur<-NA

e_ad2_phd_nb_hur_beta0c<-NA

e_ad2_phd_nb_hur_beta0c_se<-NA

e_ad2_phd_nb_hur_beta1c<-NA

e_ad2_phd_nb_hur_beta1c_se<-NA

e_ad2_phd_nb_hur_logthetac<-NA

e_ad2_phd_nb_hur_logthetac_se<-NA

e_ad2_phd_nb_hur_beta0z<-NA

e_ad2_phd_nb_hur_beta0z_se<-NA

e_ad2_phd_nb_hur_beta1z<-NA

e_ad2_phd_nb_hur_beta1z_se<-NA }

if(is(xx,"try-error")) {ad2_phd_nb_hur<-NA

e_ad2_phd_nb_hur_beta0c<-NA

e_ad2_phd_nb_hur_beta0c_se<-NA

e_ad2_phd_nb_hur_beta1c<-NA

e_ad2_phd_nb_hur_beta1c_se<-NA

e_ad2_phd_nb_hur_logthetac<-NA

e_ad2_phd_nb_hur_logthetac_se<-NA

e_ad2_phd_nb_hur_beta0z<-NA

e_ad2_phd_nb_hur_beta0z_se<-NA

e_ad2_phd_nb_hur_beta1z<-NA

e_ad2_phd_nb_hur_beta1z_se<-NA }

if(is(cc,"try-error")==F & is(xx,"try-error")==F) { ad2_phd_nb_hur<-1-pchisq(-2*(hurdle(AD2~1, data=data,offset=log(S_AD),dist = "negbin")$loglik-hurdle(AD2~Phd, data=data,offset=log(S_AD),dist = "negbin")$loglik),2)

e_ad2_phd_nb_hur_beta0c<-summary(hurdle(AD2~Phd, data=data,offset=log(S_AD),dist = "negbin"))$coefficients$count[1,1]

e_ad2_phd_nb_hur_beta0c_se<-summary(hurdle(AD2~Phd, data=data,offset=log(S_AD),dist = "negbin"))$coefficients$count[1,2]

e_ad2_phd_nb_hur_beta1c<-summary(hurdle(AD2~Phd, data=data,offset=log(S_AD),dist = "negbin"))$coefficients$count[2,1]

e_ad2_phd_nb_hur_beta1c_se<-summary(hurdle(AD2~Phd, data=data,offset=log(S_AD),dist = "negbin"))$coefficients$count[2,2]

e_ad2_phd_nb_hur_logthetac<-summary(hurdle(AD2~Phd, data=data,offset=log(S_AD),dist = "negbin"))$coefficients$count[3,1]

e_ad2_phd_nb_hur_logthetac_se<-summary(hurdle(AD2~Phd, data=data,offset=log(S_AD),dist = "negbin"))$coefficients$count[3,2]

e_ad2_phd_nb_hur_beta0z<-summary(hurdle(AD2~Phd, data=data,offset=log(S_AD),dist = "negbin"))$coefficients$zero[1,1]

e_ad2_phd_nb_hur_beta0z_se<-summary(hurdle(AD2~Phd, data=data,offset=log(S_AD),dist = "negbin"))$coefficients$zero[1,2]

e_ad2_phd_nb_hur_beta1z<-summary(hurdle(AD2~Phd, data=data,offset=log(S_AD),dist = "negbin"))$coefficients$zero[2,1]

e_ad2_phd_nb_hur_beta1z_se<-summary(hurdle(AD2~Phd, data=data,offset=log(S_AD),dist = "negbin"))$coefficients$zero[2,2] }

#######################################################################################################################

#################################### ratio~Phd ###############################################################

#######################################################################################################################

#standard linear model Phd~AD2

cc <- try(lm(ratio~Phd, data = data), silent=T)

xx<- try(summary(lm(ratio~Phd, data = data)), silent=T)

if(is(cc,"try-error")) { ratio_phd_lm<-NA

e_ratio_phd_lm_beta0<-NA

e_ratio_phd_lm_beta0_se<-NA

e_ratio_phd_lm_beta1<-NA

e_ratio_phd_lm_beta1_se<-NA}

if(is(xx,"try-error")) { ratio_phd_lm<-NA

e_ratio_phd_lm_beta0<-NA

e_ratio_phd_lm_beta0_se<-NA

e_ratio_phd_lm_beta1<-NA

e_ratio_phd_lm_beta1_se<-NA}

if(is(cc,"try-error")==F & is(xx,"try-error")==F) { ratio_phd_lm<-summary(lm(ratio~Phd, data = data))$coefficients[2,4]

e_ratio_phd_lm_beta0<-summary(lm(ratio~Phd, data = data))$coefficients[1,1]

e_ratio_phd_lm_beta0_se<-summary(lm(ratio~Phd, data = data))$coefficients[1,2]

e_ratio_phd_lm_beta1<-summary(lm(ratio~Phd, data = data))$coefficients[2,1]

e_ratio_phd_lm_beta1_se<-summary(lm(ratio~Phd, data = data))$coefficients[2,2]}

#######################################################################################################################

#################################### Phd~ratio ###############################################################

#######################################################################################################################

#logistic regression standard Phd~ratio

cc <- try(glm(Phd~ratio, family=binomial(link=logit),data=data), silent=T)

xx<- try(summary(glm(Phd~ratio, family=binomial(link=logit),data=data), silent=T))

if(is(cc,"try-error")) { phd_ratio_logit<-NA

e_phd_ratio_logit_beta0<-NA

e_phd_ratio_logit_beta0_se<-NA

e_phd_ratio_logit_beta1<-NA

e_phd_ratio_logit_beta1_se<-NA}

if(is(xx,"try-error")) { phd_ratio_logit<-NA

e_phd_ratio_logit_beta0<-NA

e_phd_ratio_logit_beta0_se<-NA

e_phd_ratio_logit_beta1<-NA

e_phd_ratio_logit_beta1_se<-NA}

if(is(cc,"try-error")==F & is(xx,"try-error")==F) { phd_ratio_logit<-summary(glm(Phd~ratio, family=binomial(link=logit),data=data))$coefficients[2,4]

e_phd_ratio_logit_beta0<-summary(glm(Phd~ratio, family=binomial(link=logit),data=data))$coefficients[1,1]

e_phd_ratio_logit_beta0_se<-summary(glm(Phd~ratio, family=binomial(link=logit),data=data))$coefficients[1,2]

e_phd_ratio_logit_beta1<-summary(glm(Phd~ratio, family=binomial(link=logit),data=data))$coefficients[2,1]

e_phd_ratio_logit_beta1_se<-summary(glm(Phd~ratio, family=binomial(link=logit),data=data))$coefficients[2,2]}

#######################################################################################################################

#################################### GT_estim~Phd ############################################################

#######################################################################################################################

#ordinal logistic standard model GT_estim~Phd

cc <- try(polr(GT_estim~Phd, data = data, Hess = TRUE), silent=T)

if(is(cc,"try-error")) {gt_phd_ordinal_logit<-NA

e_gt_phd_ordinal_logit_coef<-NA

e_gt_phd_ordinal_logit_coef_se<-NA

e_gt_phd_ordinal_logit_int1<-NA

e_gt_phd_ordinal_logit_int1_se<-NA

e_gt_phd_ordinal_logit_int2<-NA

e_gt_phd_ordinal_logit_int2_se<-NA}

if(is(cc,"try-error")==F) {gt_phd_ordinal_logit<-pnorm(abs(summary( polr(GT_estim~Phd, data = data, Hess = TRUE) )$coefficients[1,3]),lower.tail=F)*2

e_gt_phd_ordinal_logit_coef<-summary( polr(GT_estim~Phd, data = data, Hess = TRUE) )$coefficients[1,1]

e_gt_phd_ordinal_logit_coef_se<-summary( polr(GT_estim~Phd, data = data, Hess = TRUE) )$coefficients[1,2]

e_gt_phd_ordinal_logit_int1<-summary( polr(GT_estim~Phd, data = data, Hess = TRUE) )$coefficients[2,1]

e_gt_phd_ordinal_logit_int1_se<-summary( polr(GT_estim~Phd, data = data, Hess = TRUE) )$coefficients[2,2]

e_gt_phd_ordinal_logit_int2<-summary( polr(GT_estim~Phd, data = data, Hess = TRUE) )$coefficients[3,1]

e_gt_phd_ordinal_logit_int2_se<-summary( polr(GT_estim~Phd, data = data, Hess = TRUE) )$coefficients[3,2]}

#logistic regression standard GT_estim_g~Phd

cc <- try(glm(GT_estim_g~Phd, family=binomial(link=logit),data=data), silent=T)

xx<- try(summary(glm(GT_estim_g~Phd, family=binomial(link=logit),data=data), silent=T))

if(is(cc,"try-error")) { gt_phd_logit<-NA

e_gt_phd_logit_beta0<-NA

e_gt_phd_logit_beta0_se<-NA

e_gt_phd_logit_beta1<-NA

e_gt_phd_logit_beta1_se<-NA}

if(is(xx,"try-error")) { gt_phd_logit<-NA

e_gt_phd_logit_beta0<-NA

e_gt_phd_logit_beta0_se<-NA

e_gt_phd_logit_beta1<-NA

e_gt_phd_logit_beta1_se<-NA}

if(is(cc,"try-error")==F & is(xx,"try-error")==F) { gt_phd_logit<-summary(glm(GT_estim_g~Phd, family=binomial(link=logit),data=data))$coefficients[2,4]

e_gt_phd_logit_beta0<-summary(glm(GT_estim_g~Phd, family=binomial(link=logit),data=data))$coefficients[1,1]

e_gt_phd_logit_beta0_se<-summary(glm(GT_estim_g~Phd, family=binomial(link=logit),data=data))$coefficients[1,2]

e_gt_phd_logit_beta1<-summary(glm(GT_estim_g~Phd, family=binomial(link=logit),data=data))$coefficients[2,1]

e_gt_phd_logit_beta1_se<-summary(glm(GT_estim_g~Phd, family=binomial(link=logit),data=data))$coefficients[2,2]}

#######################################################################################################################

#################################### Phd~GT_estim ###########################################################

#######################################################################################################################

#logistic regression standard Phd~GT_estim

cc <- try(glm(Phd~GT_estim, family=binomial(link=logit),data=data), silent=T)

xx<- try(summary(glm(Phd~GT_estim, family=binomial(link=logit),data=data), silent=T))

if(is(cc,"try-error")) { phd_gt_logit<-NA

e_phd_gt_logit_beta0<-NA

e_phd_gt_logit_beta0_se<-NA

e_phd_gt_logit_beta1<-NA

e_phd_gt_logit_beta1_se<-NA}

if(is(xx,"try-error")) { phd_gt_logit<-NA

e_phd_gt_logit_beta0<-NA

e_phd_gt_logit_beta0_se<-NA

e_phd_gt_logit_beta1<-NA

e_phd_gt_logit_beta1_se<-NA}

if(is(cc,"try-error")==F & is(xx,"try-error")==F) { phd_gt_logit<-summary(glm(Phd~GT_estim, family=binomial(link=logit),data=data))$coefficients[2,4]

e_phd_gt_logit_beta0<-summary(glm(Phd~GT_estim, family=binomial(link=logit),data=data))$coefficients[1,1]

e_phd_gt_logit_beta0_se<-summary(glm(Phd~GT_estim, family=binomial(link=logit),data=data))$coefficients[1,2]

e_phd_gt_logit_beta1<-summary(glm(Phd~GT_estim, family=binomial(link=logit),data=data))$coefficients[2,1]

e_phd_gt_logit_beta1_se<-summary(glm(Phd~GT_estim, family=binomial(link=logit),data=data))$coefficients[2,2]}

#######################################################################################################################

#################################### GT_real~Phd ############################################################

#######################################################################################################################

#ordinal logistic standard model GT_real~Phd

cc <- try(polr(GT_real~Phd, data = data, Hess = TRUE), silent=T)

if(is(cc,"try-error")) {gtr_phd_ordinal_logit<-NA

e_gtr_phd_ordinal_logit_coef<-NA

e_gtr_phd_ordinal_logit_coef_se<-NA

e_gtr_phd_ordinal_logit_int1<-NA

e_gtr_phd_ordinal_logit_int1_se<-NA

e_gtr_phd_ordinal_logit_int2<-NA

e_gtr_phd_ordinal_logit_int2_se<-NA}

if(is(cc,"try-error")==F) {gtr_phd_ordinal_logit<-pnorm(abs(summary( polr(GT_real~Phd, data = data, Hess = TRUE) )$coefficients[1,3]),lower.tail=F)*2

e_gtr_phd_ordinal_logit_coef<-summary( polr(GT_real~Phd, data = data, Hess = TRUE) )$coefficients[1,1]

e_gtr_phd_ordinal_logit_coef_se<-summary( polr(GT_real~Phd, data = data, Hess = TRUE) )$coefficients[1,2]

e_gtr_phd_ordinal_logit_int1<-summary( polr(GT_real~Phd, data = data, Hess = TRUE) )$coefficients[2,1]

e_gtr_phd_ordinal_logit_int1_se<-summary( polr(GT_real~Phd, data = data, Hess = TRUE) )$coefficients[2,2]

e_gtr_phd_ordinal_logit_int2<-summary( polr(GT_real~Phd, data = data, Hess = TRUE) )$coefficients[3,1]

e_gtr_phd_ordinal_logit_int2_se<-summary( polr(GT_real~Phd, data = data, Hess = TRUE) )$coefficients[3,2]}

#logistic regression standard GT_real~Phd

cc <- try(glm(GT_real~Phd, family=binomial(link=logit),data=data), silent=T)

xx<- try(summary(glm(GT_real~Phd, family=binomial(link=logit),data=data), silent=T))

if(is(cc,"try-error")) { gtr_phd_logit<-NA

e_gtr_phd_logit_beta0<-NA

e_gtr_phd_logit_beta0_se<-NA

e_gtr_phd_logit_beta1<-NA

e_gtr_phd_logit_beta1_se<-NA}

if(is(xx,"try-error")) { gtr_phd_logit<-NA

e_gtr_phd_logit_beta0<-NA

e_gtr_phd_logit_beta0_se<-NA

e_gtr_phd_logit_beta1<-NA

e_gtr_phd_logit_beta1_se<-NA}

if(is(cc,"try-error")==F & is(xx,"try-error")==F) { gtr_phd_logit<-summary(glm(GT_real~Phd, family=binomial(link=logit),data=data))$coefficients[2,4]

e_gtr_phd_logit_beta0<-summary(glm(GT_real~Phd, family=binomial(link=logit),data=data))$coefficients[1,1]

e_gtr_phd_logit_beta0_se<-summary(glm(GT_real~Phd, family=binomial(link=logit),data=data))$coefficients[1,2]

e_gtr_phd_logit_beta1<-summary(glm(GT_real~Phd, family=binomial(link=logit),data=data))$coefficients[2,1]

e_gtr_phd_logit_beta1_se<-summary(glm(GT_real~Phd, family=binomial(link=logit),data=data))$coefficients[2,2]}

#######################################################################################################################

#################################### Phd~GT_real ###########################################################

#######################################################################################################################

#logistic regression standard Phd~GT_real

cc <- try(glm(Phd~GT_real, family=binomial(link=logit),data=data), silent=T)

xx<- try(summary(glm(Phd~GT_real, family=binomial(link=logit),data=data), silent=T))

if(is(cc,"try-error")) { phd_gtr_logit<-NA

e_phd_gtr_logit_beta0<-NA

e_phd_gtr_logit_beta0_se<-NA

e_phd_gtr_logit_beta1<-NA

e_phd_gtr_logit_beta1_se<-NA}

if(is(xx,"try-error")) { phd_gtr_logit<-NA

e_phd_gtr_logit_beta0<-NA

e_phd_gtr_logit_beta0_se<-NA

e_phd_gtr_logit_beta1<-NA

e_phd_gtr_logit_beta1_se<-NA}

if(is(cc,"try-error")==F & is(xx,"try-error")==F) { phd_gtr_logit<-summary(glm(Phd~GT_real, family=binomial(link=logit),data=data))$coefficients[2,4]

e_phd_gtr_logit_beta0<-summary(glm(Phd~GT_real, family=binomial(link=logit),data=data))$coefficients[1,1]

e_phd_gtr_logit_beta0_se<-summary(glm(Phd~GT_real, family=binomial(link=logit),data=data))$coefficients[1,2]

e_phd_gtr_logit_beta1<-summary(glm(Phd~GT_real, family=binomial(link=logit),data=data))$coefficients[2,1]

e_phd_gtr_logit_beta1_se<-summary(glm(Phd~GT_real, family=binomial(link=logit),data=data))$coefficients[2,2]}

#saving results

test_df<-data.frame(rs=gt_pos$rs,

alleles=gt_pos$alleles,

chrom=gt_pos$chrom,

pos=gt_pos$pos,

ad2_ph_poisson,

ad2_ph_nb,

ad2_ph_nb_zi,

ad2_ph_nb_hur,

ratio_ph_lm,

ph_ratio_lm,

gt_ph_ordinal_logit,

gt_ph_logit,

ph_gt_lm,

gtr_ph_ordinal_logit,

gtr_ph_logit,

ph_gtr_lm,

ad2_phd_poisson,

ad2_phd_nb,

ad2_phd_nb_zi,

ad2_phd_nb_hur,

ratio_phd_lm,

phd_ratio_logit,

gt_phd_ordinal_logit,

gt_phd_logit,

phd_gt_logit,

gtr_phd_ordinal_logit,

gtr_phd_logit,

phd_gtr_logit)

results_pvalue<-rbind(results_pvalue,test_df)

estim_df<-data.frame(rs=gt_pos$rs,

alleles=gt_pos$alleles,

chrom=gt_pos$chrom,

pos=gt_pos$pos,

e_ad2_phd_poisson_beta0 ,

e_ad2_phd_poisson_beta0_se ,

e_ad2_phd_poisson_beta1 ,

e_ad2_phd_poisson_beta1_se ,

e_ad2_phd_nb_beta0 ,

e_ad2_phd_nb_beta0_se ,

e_ad2_phd_nb_beta1 ,

e_ad2_phd_nb_beta1_se ,

e_ad2_phd_nb_zi_beta0c ,

e_ad2_phd_nb_zi_beta0c_se ,

e_ad2_phd_nb_zi_beta1c ,

e_ad2_phd_nb_zi_beta1c_se ,

e_ad2_phd_nb_zi_logthetac ,

e_ad2_phd_nb_zi_logthetac_se ,

e_ad2_phd_nb_zi_beta0z ,

e_ad2_phd_nb_zi_beta0z_se ,

e_ad2_phd_nb_zi_beta1z ,

e_ad2_phd_nb_zi_beta1z_se ,

e_ad2_phd_nb_hur_beta0c ,

e_ad2_phd_nb_hur_beta0c_se ,

e_ad2_phd_nb_hur_beta1c ,

e_ad2_phd_nb_hur_beta1c_se ,

e_ad2_phd_nb_hur_logthetac ,

e_ad2_phd_nb_hur_logthetac_se ,

e_ad2_phd_nb_hur_beta0z ,

e_ad2_phd_nb_hur_beta0z_se ,

e_ad2_phd_nb_hur_beta1z ,

e_ad2_phd_nb_hur_beta1z_se ,

e_ratio_phd_lm_beta0 ,

e_ratio_phd_lm_beta0_se ,

e_ratio_phd_lm_beta1 ,

e_ratio_phd_lm_beta1_se ,

e_phd_ratio_logit_beta0 ,

e_phd_ratio_logit_beta0_se ,

e_phd_ratio_logit_beta1 ,

e_phd_ratio_logit_beta1_se ,

e_gt_phd_ordinal_logit_coef ,

e_gt_phd_ordinal_logit_coef_se ,

e_gt_phd_ordinal_logit_int1 ,

e_gt_phd_ordinal_logit_int1_se ,

e_gt_phd_ordinal_logit_int2 ,

e_gt_phd_ordinal_logit_int2_se ,

e_gt_phd_logit_beta0 ,

e_gt_phd_logit_beta0_se ,

e_gt_phd_logit_beta1 ,

e_gt_phd_logit_beta1_se ,

e_phd_gt_logit_beta0 ,

e_phd_gt_logit_beta0_se ,

e_phd_gt_logit_beta1 ,

e_phd_gt_logit_beta1_se ,

e_gtr_phd_ordinal_logit_coef ,

e_gtr_phd_ordinal_logit_coef_se ,

e_gtr_phd_ordinal_logit_int1 ,

e_gtr_phd_ordinal_logit_int1_se ,

e_gtr_phd_ordinal_logit_int2 ,

e_gtr_phd_ordinal_logit_int2_se ,

e_gtr_phd_logit_beta0 ,

e_gtr_phd_logit_beta0_se ,

e_gtr_phd_logit_beta1 ,

e_gtr_phd_logit_beta1_se ,

e_phd_gtr_logit_beta0 ,

e_phd_gtr_logit_beta0_se ,

e_phd_gtr_logit_beta1 ,

e_phd_gtr_logit_beta1_se )

results_estim<-rbind(results_estim,estim_df)

test_variant<-data.frame(rs=gt_pos$rs,

alleles=gt_pos$alleles,

chrom=gt_pos$chrom,

pos=gt_pos$pos,medianGQ,

medianSAD)

test_table<-data.frame(rs=gt_pos$rs,

alleles=gt_pos$alleles,

chrom=gt_pos$chrom,

pos=gt_pos$pos,r0e0,

r0e1,

r0e2,

r1e0,

r1e1,

r1e2,

r2e0,

r2e1,

r2e2)

results_variant<-rbind(results_variant,test_variant)

results_table<-rbind(results_table,test_table)

write.table(results_pvalue,"results_pvalue_a1.txt")

write.table(results_estim,"results_estim_a1.txt")

write.table(results_variant,"results_variant_a1.txt")

write.table(results_table,"results_table_a1.txt")

b<-Sys.time()

print(b-a)

} #fin position p

#simulating null scenario

#estimation gt

estim_gt<-function(data){

data<-cbind(data,GT="5/5")

data$GT<-as.character(data$GT)

for (i in 1:length(data[,1])){

hom_ref<-(1/3)*((1-10^(-data$GQ[i]/10))^data$AD1[i])*((10^(-data$GQ[i]/10))^data$AD2[i])

het<-(1/3)*(((1)/2)^data$AD1[i])*(((1)/2)^data$AD2[i])

hom_alt<-(1/3)*((10^(-data$GQ[i]/10))^data$AD1[i])*((1-10^(-data$GQ[i]/10))^data$AD2[i])

if(which.max(c(hom_ref,het,hom_alt))==1) {data[i,4]<-"0/0"}

if(which.max(c(hom_ref,het,hom_alt))==2) {data[i,4]<-as.character("0/1")}

if(which.max(c(hom_ref,het,hom_alt))==3) {data[i,4]<-"1/1"}

}

return(data)

}

print("loading libraries")

library("tmvtnorm")

library(robust)

library(pscl)

library(gamlss.dist)

library("grDevices", lib.loc="~/R/R-3.2.2/library")

Sys.setenv(LANG = "en")

a<-Sys.time()

results_pvalue<-data.frame(rs=0,

alleles=0,

chrom=0,

pos=0,

ad2_ph_poisson=0,

ad2_ph_nb=0,

ad2_ph_nb_zi=0,

ad2_ph_nb_hur=0,

ratio_ph_lm=0,

ph_ratio_lm=0,

gt_ph_ordinal_logit=0,

gt_ph_logit=0,

ph_gt_lm=0,

gtr_ph_ordinal_logit=0,

gtr_ph_logit=0,

ph_gtr_lm=0,

ad2_phd_poisson=0,

ad2_phd_nb=0,

ad2_phd_nb_zi=0,

ad2_phd_nb_hur=0,

ratio_phd_lm=0,

phd_ratio_logit=0,

gt_phd_ordinal_logit=0,

gt_phd_logit=0,

phd_gt_logit=0,

gtr_phd_ordinal_logit=0,

gtr_phd_logit=0,

phd_gtr_logit=0)

results_estim<-data.frame(rs=0,

alleles=0,

chrom=0,

pos=0,

e_ad2_phd_poisson_beta0=0,

e_ad2_phd_poisson_beta0_se=0,

e_ad2_phd_poisson_beta1=0,

e_ad2_phd_poisson_beta1_se=0,

e_ad2_phd_nb_beta0=0,

e_ad2_phd_nb_beta0_se=0,

e_ad2_phd_nb_beta1=0,

e_ad2_phd_nb_beta1_se=0,

e_ad2_phd_nb_zi_beta0c=0,

e_ad2_phd_nb_zi_beta0c_se=0,

e_ad2_phd_nb_zi_beta1c=0,

e_ad2_phd_nb_zi_beta1c_se=0,

e_ad2_phd_nb_zi_logthetac=0,

e_ad2_phd_nb_zi_logthetac_se=0,

e_ad2_phd_nb_zi_beta0z=0,

e_ad2_phd_nb_zi_beta0z_se=0,

e_ad2_phd_nb_zi_beta1z=0,

e_ad2_phd_nb_zi_beta1z_se=0,

e_ad2_phd_nb_hur_beta0c=0,

e_ad2_phd_nb_hur_beta0c_se=0,

e_ad2_phd_nb_hur_beta1c=0,

e_ad2_phd_nb_hur_beta1c_se=0,

e_ad2_phd_nb_hur_logthetac=0,

e_ad2_phd_nb_hur_logthetac_se=0,

e_ad2_phd_nb_hur_beta0z=0,

e_ad2_phd_nb_hur_beta0z_se=0,

e_ad2_phd_nb_hur_beta1z=0,

e_ad2_phd_nb_hur_beta1z_se=0,

e_ratio_phd_lm_beta0=0,

e_ratio_phd_lm_beta0_se=0,

e_ratio_phd_lm_beta1=0,

e_ratio_phd_lm_beta1_se=0,

e_phd_ratio_logit_beta0=0,

e_phd_ratio_logit_beta0_se=0,

e_phd_ratio_logit_beta1=0,

e_phd_ratio_logit_beta1_se=0,

e_gt_phd_ordinal_logit_coef=0,

e_gt_phd_ordinal_logit_coef_se=0,

e_gt_phd_ordinal_logit_int1=0,

e_gt_phd_ordinal_logit_int1_se=0,

e_gt_phd_ordinal_logit_int2=0,

e_gt_phd_ordinal_logit_int2_se=0,

e_gt_phd_logit_beta0=0,

e_gt_phd_logit_beta0_se=0,

e_gt_phd_logit_beta1=0,

e_gt_phd_logit_beta1_se=0,

e_phd_gt_logit_beta0=0,

e_phd_gt_logit_beta0_se=0,

e_phd_gt_logit_beta1=0,

e_phd_gt_logit_beta1_se=0,

e_gtr_phd_ordinal_logit_coef=0,

e_gtr_phd_ordinal_logit_coef_se=0,

e_gtr_phd_ordinal_logit_int1=0,

e_gtr_phd_ordinal_logit_int1_se=0,

e_gtr_phd_ordinal_logit_int2=0,

e_gtr_phd_ordinal_logit_int2_se=0,

e_gtr_phd_logit_beta0=0,

e_gtr_phd_logit_beta0_se=0,

e_gtr_phd_logit_beta1=0,

e_gtr_phd_logit_beta1_se=0,

e_phd_gtr_logit_beta0=0,

e_phd_gtr_logit_beta0_se=0,

e_phd_gtr_logit_beta1=0,

e_phd_gtr_logit_beta1_se=0)

results_variant<-data.frame(rs=0,

alleles=0,

chrom=0,

pos=0,

medianGQ=0,

medianSAD=0)

results_table<-data.frame(rs=0,

alleles=0,

chrom=0,

pos=0,

r0e0=0,

r0e1=0,

r0e2=0,

r1e0=0,

r1e1=0,

r1e2=0,

r2e0=0,

r2e1=0,

r2e2=0)

#read real genotypes

print("reading files")

gt<- read.table("gt_simulated.txt", header=TRUE, quote="\"")

#preparation

print("preparing files")

#CC TT CT

#read and modify lists

C_T_CT <- read.table("C_T_CT.txt", header=TRUE, quote="\"")

C_T_TT <- read.table("C_T_TT.txt", header=TRUE, quote="\"")

C_T_CC <- read.table("C_T_CC.txt", header=TRUE, quote="\"")

#make c_T_CC in the correct order

C_T_CC<-data.frame(C_T_CC[,1:4],AD1=C_T_CC$AD1,AD2=C_T_CC$AD2,C_T_CC[,5:7])

#modify AD1 and AD2

C_T_CT<-data.frame(C_T_CT,AD=paste(C_T_CT$AD1,",",C_T_CT$AD2,",",C_T_CT$GQ))

C_T_CT$AD<-as.character(C_T_CT$AD)

C_T_CT$AD1<-as.numeric(C_T_CT$AD1)

C_T_CT$AD2<-as.numeric(C_T_CT$AD2)

C_T_CT$GQ<-as.numeric(C_T_CT$GQ)

C_T_CC<-data.frame(C_T_CC,AD=paste(C_T_CC$AD1,",",C_T_CC$AD2,",",C_T_CC$GQ))

C_T_CC$AD<-as.character(C_T_CC$AD)

C_T_CC$AD1<-as.numeric(C_T_CC$AD1)

C_T_CC$AD2<-as.numeric(C_T_CC$AD2)

C_T_CC$GQ<-as.numeric(C_T_CC$GQ)

C_T_TT<-data.frame(C_T_TT,AD=paste(C_T_TT$AD1,",",C_T_TT$AD2,",",C_T_TT$GQ))

C_T_TT$AD<-as.character(C_T_TT$AD)

C_T_TT$AD1<-as.numeric(C_T_TT$AD1)

C_T_TT$AD2<-as.numeric(C_T_TT$AD2)

C_T_TT$GQ<-as.numeric(C_T_TT$GQ)

for( i in 1:length(C_T_CT$GQ)){

if(C_T_CT$GQ[i]<=20){C_T_CT$G_GQ[i]<-1}

if(C_T_CT$GQ[i]>20&&C_T_CT$GQ[i]<=40){C_T_CT$G_GQ[i]<-2}

if(C_T_CT$GQ[i]>40&&C_T_CT$GQ[i]<=60){C_T_CT$G_GQ[i]<-3}

if(C_T_CT$GQ[i]>60&&C_T_CT$GQ[i]<=80){C_T_CT$G_GQ[i]<-4}

if(C_T_CT$GQ[i]>80&&C_T_CT$GQ[i]<99){C_T_CT$G_GQ[i]<-5}

if(C_T_CT$GQ[i]>=99){C_T_CT$G_GQ[i]<-6}

}

f_GQ_C_T_CT<-table(C_T_CT$G_GQ)/length(C_T_CT$G_GQ)

for( i in 1:length(C_T_CC$GQ)){

if(C_T_CC$GQ[i]<=20){C_T_CC$G_GQ[i]<-1}

if(C_T_CC$GQ[i]>20&&C_T_CC$GQ[i]<=40){C_T_CC$G_GQ[i]<-2}

if(C_T_CC$GQ[i]>40&&C_T_CC$GQ[i]<=60){C_T_CC$G_GQ[i]<-3}

if(C_T_CC$GQ[i]>60&&C_T_CC$GQ[i]<=80){C_T_CC$G_GQ[i]<-4}

if(C_T_CC$GQ[i]>80&&C_T_CC$GQ[i]<99){C_T_CC$G_GQ[i]<-5}

if(C_T_CC$GQ[i]>=99){C_T_CC$G_GQ[i]<-6}

}

f_GQ_C_T_CC<-table(C_T_CC$G_GQ)/length(C_T_CC$G_GQ)

for( i in 1:length(C_T_TT$GQ)){

if(C_T_TT$GQ[i]<=20){C_T_TT$G_GQ[i]<-1}

if(C_T_TT$GQ[i]>20&&C_T_TT$GQ[i]<=40){C_T_TT$G_GQ[i]<-2}

if(C_T_TT$GQ[i]>40&&C_T_TT$GQ[i]<=60){C_T_TT$G_GQ[i]<-3}

if(C_T_TT$GQ[i]>60&&C_T_TT$GQ[i]<=80){C_T_TT$G_GQ[i]<-4}

if(C_T_TT$GQ[i]>80&&C_T_TT$GQ[i]<99){C_T_TT$G_GQ[i]<-5}

if(C_T_TT$GQ[i]>=99){C_T_TT$G_GQ[i]<-6}

}

f_GQ_C_T_TT<-table(C_T_TT$G_GQ)/length(C_T_TT$G_GQ)

#GG TT GT

#read and modify lists

G_T_GT <- read.table("G_T_GT.txt", header=TRUE, quote="\"")

G_T_TT <- read.table("G_T_TT.txt", header=TRUE, quote="\"")

G_T_GG <- read.table("G_T_GG.txt", header=TRUE, quote="\"")

#make c_T_CC in the correct order

G_T_GG<-data.frame(G_T_GG[,1:4],AD1=G_T_GG$AD1,AD2=G_T_GG$AD2,G_T_GG[,5:7])

#modify AD1 and AD2

G_T_GT<-data.frame(G_T_GT,AD=paste(G_T_GT$AD1,",",G_T_GT$AD2,",",G_T_GT$GQ))

G_T_GT$AD<-as.character(G_T_GT$AD)

G_T_GT$AD1<-as.numeric(G_T_GT$AD1)

G_T_GT$AD2<-as.numeric(G_T_GT$AD2)

G_T_GT$GQ<-as.numeric(G_T_GT$GQ)

G_T_GG<-data.frame(G_T_GG,AD=paste(G_T_GG$AD1,",",G_T_GG$AD2,",",G_T_GG$GQ))

G_T_GG$AD<-as.character(G_T_GG$AD)

G_T_GG$AD1<-as.numeric(G_T_GG$AD1)

G_T_GG$AD2<-as.numeric(G_T_GG$AD2)

G_T_GG$GQ<-as.numeric(G_T_GG$GQ)

G_T_TT<-data.frame(G_T_TT,AD=paste(G_T_TT$AD1,",",G_T_TT$AD2,",",G_T_TT$GQ))

G_T_TT$AD<-as.character(G_T_TT$AD)

G_T_TT$AD1<-as.numeric(G_T_TT$AD1)

G_T_TT$AD2<-as.numeric(G_T_TT$AD2)

G_T_TT$GQ<-as.numeric(G_T_TT$GQ)

for( i in 1:length(G_T_GT$GQ)){

if(G_T_GT$GQ[i]<=20){G_T_GT$G_GQ[i]<-1}

if(G_T_GT$GQ[i]>20&&G_T_GT$GQ[i]<=40){G_T_GT$G_GQ[i]<-2}

if(G_T_GT$GQ[i]>40&&G_T_GT$GQ[i]<=60){G_T_GT$G_GQ[i]<-3}

if(G_T_GT$GQ[i]>60&&G_T_GT$GQ[i]<=80){G_T_GT$G_GQ[i]<-4}

if(G_T_GT$GQ[i]>80&&G_T_GT$GQ[i]<99){G_T_GT$G_GQ[i]<-5}

if(G_T_GT$GQ[i]>=99){G_T_GT$G_GQ[i]<-6}

}

f_GQ_G_T_GT<-table(G_T_GT$G_GQ)/length(G_T_GT$G_GQ)

for( i in 1:length(G_T_GG$GQ)){

if(G_T_GG$GQ[i]<=20){G_T_GG$G_GQ[i]<-1}

if(G_T_GG$GQ[i]>20&&G_T_GG$GQ[i]<=40){G_T_GG$G_GQ[i]<-2}

if(G_T_GG$GQ[i]>40&&G_T_GG$GQ[i]<=60){G_T_GG$G_GQ[i]<-3}

if(G_T_GG$GQ[i]>60&&G_T_GG$GQ[i]<=80){G_T_GG$G_GQ[i]<-4}

if(G_T_GG$GQ[i]>80&&G_T_GG$GQ[i]<99){G_T_GG$G_GQ[i]<-5}

if(G_T_GG$GQ[i]>=99){G_T_GG$G_GQ[i]<-6}

}

f_GQ_G_T_GG<-table(G_T_GG$G_GQ)/length(G_T_GG$G_GQ)

for( i in 1:length(G_T_TT$GQ)){

if(G_T_TT$GQ[i]<=20){G_T_TT$G_GQ[i]<-1}

if(G_T_TT$GQ[i]>20&&G_T_TT$GQ[i]<=40){G_T_TT$G_GQ[i]<-2}

if(G_T_TT$GQ[i]>40&&G_T_TT$GQ[i]<=60){G_T_TT$G_GQ[i]<-3}

if(G_T_TT$GQ[i]>60&&G_T_TT$GQ[i]<=80){G_T_TT$G_GQ[i]<-4}

if(G_T_TT$GQ[i]>80&&G_T_TT$GQ[i]<99){G_T_TT$G_GQ[i]<-5}

if(G_T_TT$GQ[i]>=99){G_T_TT$G_GQ[i]<-6}

}

f_GQ_G_T_TT<-table(G_T_TT$G_GQ)/length(G_T_TT$G_GQ)

#CG GG CC

#read and modify lists

C_G_CG <- read.table("C_G_CG.txt", header=TRUE, quote="\"")

C_G_GG <- read.table("C_G_GG.txt", header=TRUE, quote="\"")

C_G_CC <- read.table("C_G_CC.txt", header=TRUE, quote="\"")

#make c_T_CC in the correct order

C_G_CC<-data.frame(C_G_CC[,1:4],AD1=C_G_CC$AD1,AD2=C_G_CC$AD2,C_G_CC[,5:7])

#modify AD1 and AD2

C_G_CG<-data.frame(C_G_CG,AD=paste(C_G_CG$AD1,",",C_G_CG$AD2,",",C_G_CG$GQ))

C_G_CG$AD<-as.character(C_G_CG$AD)

C_G_CG$AD1<-as.numeric(C_G_CG$AD1)

C_G_CG$AD2<-as.numeric(C_G_CG$AD2)

C_G_CG$GQ<-as.numeric(C_G_CG$GQ)

C_G_CC<-data.frame(C_G_CC,AD=paste(C_G_CC$AD1,",",C_G_CC$AD2,",",C_G_CC$GQ))

C_G_CC$AD<-as.character(C_G_CC$AD)

C_G_CC$AD1<-as.numeric(C_G_CC$AD1)

C_G_CC$AD2<-as.numeric(C_G_CC$AD2)

C_G_CC$GQ<-as.numeric(C_G_CC$GQ)

C_G_GG<-data.frame(C_G_GG,AD=paste(C_G_GG$AD1,",",C_G_GG$AD2,",",C_G_GG$GQ))

C_G_GG$AD<-as.character(C_G_GG$AD)

C_G_GG$AD1<-as.numeric(C_G_GG$AD1)

C_G_GG$AD2<-as.numeric(C_G_GG$AD2)

C_G_GG$GQ<-as.numeric(C_G_GG$GQ)

for( i in 1:length(C_G_CG$GQ)){

if(C_G_CG$GQ[i]<=20){C_G_CG$G_GQ[i]<-1}

if(C_G_CG$GQ[i]>20&&C_G_CG$GQ[i]<=40){C_G_CG$G_GQ[i]<-2}

if(C_G_CG$GQ[i]>40&&C_G_CG$GQ[i]<=60){C_G_CG$G_GQ[i]<-3}

if(C_G_CG$GQ[i]>60&&C_G_CG$GQ[i]<=80){C_G_CG$G_GQ[i]<-4}

if(C_G_CG$GQ[i]>80&&C_G_CG$GQ[i]<99){C_G_CG$G_GQ[i]<-5}

if(C_G_CG$GQ[i]>=99){C_G_CG$G_GQ[i]<-6}

}

f_GQ_C_G_CG<-table(C_G_CG$G_GQ)/length(C_G_CG$G_GQ)

for( i in 1:length(C_G_CC$GQ)){

if(C_G_CC$GQ[i]<=20){C_G_CC$G_GQ[i]<-1}

if(C_G_CC$GQ[i]>20&&C_G_CC$GQ[i]<=40){C_G_CC$G_GQ[i]<-2}

if(C_G_CC$GQ[i]>40&&C_G_CC$GQ[i]<=60){C_G_CC$G_GQ[i]<-3}

if(C_G_CC$GQ[i]>60&&C_G_CC$GQ[i]<=80){C_G_CC$G_GQ[i]<-4}

if(C_G_CC$GQ[i]>80&&C_G_CC$GQ[i]<99){C_G_CC$G_GQ[i]<-5}

if(C_G_CC$GQ[i]>=99){C_G_CC$G_GQ[i]<-6}

}

f_GQ_C_G_CC<-table(C_G_CC$G_GQ)/length(C_G_CC$G_GQ)

for( i in 1:length(C_G_GG$GQ)){

if(C_G_GG$GQ[i]<=20){C_G_GG$G_GQ[i]<-1}

if(C_G_GG$GQ[i]>20&&C_G_GG$GQ[i]<=40){C_G_GG$G_GQ[i]<-2}

if(C_G_GG$GQ[i]>40&&C_G_GG$GQ[i]<=60){C_G_GG$G_GQ[i]<-3}

if(C_G_GG$GQ[i]>60&&C_G_GG$GQ[i]<=80){C_G_GG$G_GQ[i]<-4}

if(C_G_GG$GQ[i]>80&&C_G_GG$GQ[i]<99){C_G_GG$G_GQ[i]<-5}

if(C_G_GG$GQ[i]>=99){C_G_GG$G_GQ[i]<-6}

}

f_GQ_C_G_GG<-table(C_G_GG$G_GQ)/length(C_G_GG$G_GQ)

#AA TT AT

#read and modify lists

A_T_AT <- read.table("A_T_AT.txt", header=TRUE, quote="\"")

A_T_TT <- read.table("A_T_TT.txt", header=TRUE, quote="\"")

A_T_AA <- read.table("A_T_AA.txt", header=TRUE, quote="\"")

#make c_T_CC in the correct order

A_T_AA<-data.frame(A_T_AA[,1:4],AD1=A_T_AA$AD1,AD2=A_T_AA$AD2,A_T_AA[,5:7])

#modify AD1 and AD2

A_T_AT<-data.frame(A_T_AT,AD=paste(A_T_AT$AD1,",",A_T_AT$AD2,",",A_T_AT$GQ))

A_T_AT$AD<-as.character(A_T_AT$AD)

A_T_AT$AD1<-as.numeric(A_T_AT$AD1)

A_T_AT$AD2<-as.numeric(A_T_AT$AD2)

A_T_AT$GQ<-as.numeric(A_T_AT$GQ)

A_T_AA<-data.frame(A_T_AA,AD=paste(A_T_AA$AD1,",",A_T_AA$AD2,",",A_T_AA$GQ))

A_T_AA$AD<-as.character(A_T_AA$AD)

A_T_AA$AD1<-as.numeric(A_T_AA$AD1)

A_T_AA$AD2<-as.numeric(A_T_AA$AD2)

A_T_AA$GQ<-as.numeric(A_T_AA$GQ)

A_T_TT<-data.frame(A_T_TT,AD=paste(A_T_TT$AD1,",",A_T_TT$AD2,",",A_T_TT$GQ))

A_T_TT$AD<-as.character(A_T_TT$AD)

A_T_TT$AD1<-as.numeric(A_T_TT$AD1)

A_T_TT$AD2<-as.numeric(A_T_TT$AD2)

A_T_TT$GQ<-as.numeric(A_T_TT$GQ)

for( i in 1:length(A_T_AT$GQ)){

if(A_T_AT$GQ[i]<=20){A_T_AT$G_GQ[i]<-1}

if(A_T_AT$GQ[i]>20&&A_T_AT$GQ[i]<=40){A_T_AT$G_GQ[i]<-2}

if(A_T_AT$GQ[i]>40&&A_T_AT$GQ[i]<=60){A_T_AT$G_GQ[i]<-3}

if(A_T_AT$GQ[i]>60&&A_T_AT$GQ[i]<=80){A_T_AT$G_GQ[i]<-4}

if(A_T_AT$GQ[i]>80&&A_T_AT$GQ[i]<99){A_T_AT$G_GQ[i]<-5}

if(A_T_AT$GQ[i]>=99){A_T_AT$G_GQ[i]<-6}

}

f_GQ_A_T_AT<-table(A_T_AT$G_GQ)/length(A_T_AT$G_GQ)

for( i in 1:length(A_T_AA$GQ)){

if(A_T_AA$GQ[i]<=20){A_T_AA$G_GQ[i]<-1}

if(A_T_AA$GQ[i]>20&&A_T_AA$GQ[i]<=40){A_T_AA$G_GQ[i]<-2}

if(A_T_AA$GQ[i]>40&&A_T_AA$GQ[i]<=60){A_T_AA$G_GQ[i]<-3}

if(A_T_AA$GQ[i]>60&&A_T_AA$GQ[i]<=80){A_T_AA$G_GQ[i]<-4}

if(A_T_AA$GQ[i]>80&&A_T_AA$GQ[i]<99){A_T_AA$G_GQ[i]<-5}

if(A_T_AA$GQ[i]>=99){A_T_AA$G_GQ[i]<-6}

}

f_GQ_A_T_AA<-table(A_T_AA$G_GQ)/length(A_T_AA$G_GQ)

for( i in 1:length(A_T_TT$GQ)){

if(A_T_TT$GQ[i]<=20){A_T_TT$G_GQ[i]<-1}

if(A_T_TT$GQ[i]>20&&A_T_TT$GQ[i]<=40){A_T_TT$G_GQ[i]<-2}

if(A_T_TT$GQ[i]>40&&A_T_TT$GQ[i]<=60){A_T_TT$G_GQ[i]<-3}

if(A_T_TT$GQ[i]>60&&A_T_TT$GQ[i]<=80){A_T_TT$G_GQ[i]<-4}

if(A_T_TT$GQ[i]>80&&A_T_TT$GQ[i]<99){A_T_TT$G_GQ[i]<-5}

if(A_T_TT$GQ[i]>=99){A_T_TT$G_GQ[i]<-6}

}

f_GQ_A_T_TT<-table(A_T_TT$G_GQ)/length(A_T_TT$G_GQ)

#AG GG AA

#read and modify lists

A_G_AG <- read.table("A_G_AG.txt", header=TRUE, quote="\"")

A_G_GG <- read.table("A_G_GG.txt", header=TRUE, quote="\"")

A_G_AA <- read.table("A_G_AA.txt", header=TRUE, quote="\"")

#make c_T_CC in the correct order

A_G_AA<-data.frame(A_G_AA[,1:4],AD1=A_G_AA$AD1,AD2=A_G_AA$AD2,A_G_AA[,5:7])

#modify AD1 and AD2

A_G_AG<-data.frame(A_G_AG,AD=paste(A_G_AG$AD1,",",A_G_AG$AD2,",",A_G_AG$GQ))

A_G_AG$AD<-as.character(A_G_AG$AD)

A_G_AG$AD1<-as.numeric(A_G_AG$AD1)

A_G_AG$AD2<-as.numeric(A_G_AG$AD2)

A_G_AG$GQ<-as.numeric(A_G_AG$GQ)

A_G_AA<-data.frame(A_G_AA,AD=paste(A_G_AA$AD1,",",A_G_AA$AD2,",",A_G_AA$GQ))

A_G_AA$AD<-as.character(A_G_AA$AD)

A_G_AA$AD1<-as.numeric(A_G_AA$AD1)

A_G_AA$AD2<-as.numeric(A_G_AA$AD2)

A_G_AA$GQ<-as.numeric(A_G_AA$GQ)

A_G_GG<-data.frame(A_G_GG,AD=paste(A_G_GG$AD1,",",A_G_GG$AD2,",",A_G_GG$GQ))

A_G_GG$AD<-as.character(A_G_GG$AD)

A_G_GG$AD1<-as.numeric(A_G_GG$AD1)

A_G_GG$AD2<-as.numeric(A_G_GG$AD2)

A_G_GG$GQ<-as.numeric(A_G_GG$GQ)

for( i in 1:length(A_G_AG$GQ)){

if(A_G_AG$GQ[i]<=20){A_G_AG$G_GQ[i]<-1}

if(A_G_AG$GQ[i]>20&&A_G_AG$GQ[i]<=40){A_G_AG$G_GQ[i]<-2}

if(A_G_AG$GQ[i]>40&&A_G_AG$GQ[i]<=60){A_G_AG$G_GQ[i]<-3}

if(A_G_AG$GQ[i]>60&&A_G_AG$GQ[i]<=80){A_G_AG$G_GQ[i]<-4}

if(A_G_AG$GQ[i]>80&&A_G_AG$GQ[i]<99){A_G_AG$G_GQ[i]<-5}

if(A_G_AG$GQ[i]>=99){A_G_AG$G_GQ[i]<-6}

}

f_GQ_A_G_AG<-table(A_G_AG$G_GQ)/length(A_G_AG$G_GQ)

for( i in 1:length(A_G_AA$GQ)){

if(A_G_AA$GQ[i]<=20){A_G_AA$G_GQ[i]<-1}

if(A_G_AA$GQ[i]>20&&A_G_AA$GQ[i]<=40){A_G_AA$G_GQ[i]<-2}

if(A_G_AA$GQ[i]>40&&A_G_AA$GQ[i]<=60){A_G_AA$G_GQ[i]<-3}

if(A_G_AA$GQ[i]>60&&A_G_AA$GQ[i]<=80){A_G_AA$G_GQ[i]<-4}

if(A_G_AA$GQ[i]>80&&A_G_AA$GQ[i]<99){A_G_AA$G_GQ[i]<-5}

if(A_G_AA$GQ[i]>=99){A_G_AA$G_GQ[i]<-6}

}

f_GQ_A_G_AA<-table(A_G_AA$G_GQ)/length(A_G_AA$G_GQ)

for( i in 1:length(A_G_GG$GQ)){

if(A_G_GG$GQ[i]<=20){A_G_GG$G_GQ[i]<-1}

if(A_G_GG$GQ[i]>20&&A_G_GG$GQ[i]<=40){A_G_GG$G_GQ[i]<-2}

if(A_G_GG$GQ[i]>40&&A_G_GG$GQ[i]<=60){A_G_GG$G_GQ[i]<-3}

if(A_G_GG$GQ[i]>60&&A_G_GG$GQ[i]<=80){A_G_GG$G_GQ[i]<-4}

if(A_G_GG$GQ[i]>80&&A_G_GG$GQ[i]<99){A_G_GG$G_GQ[i]<-5}

if(A_G_GG$GQ[i]>=99){A_G_GG$G_GQ[i]<-6}

}

f_GQ_A_G_GG<-table(A_G_GG$G_GQ)/length(A_G_GG$G_GQ)

#AC AA CC

#read and modify lists

A_C_AC <- read.table("A_C_AC.txt", header=TRUE, quote="\"")

A_C_CC <- read.table("A_C_CC.txt", header=TRUE, quote="\"")

A_C_AA <- read.table("A_C_AA.txt", header=TRUE, quote="\"")

#make c_T_CC in the correct order

A_C_AA<-data.frame(A_C_AA[,1:4],AD1=A_C_AA$AD1,AD2=A_C_AA$AD2,A_C_AA[,5:7])

#modify AD1 and AD2

A_C_AC<-data.frame(A_C_AC,AD=paste(A_C_AC$AD1,",",A_C_AC$AD2,",",A_C_AC$GQ))

A_C_AC$AD<-as.character(A_C_AC$AD)

A_C_AC$AD1<-as.numeric(A_C_AC$AD1)

A_C_AC$AD2<-as.numeric(A_C_AC$AD2)

A_C_AC$GQ<-as.numeric(A_C_AC$GQ)

A_C_AA<-data.frame(A_C_AA,AD=paste(A_C_AA$AD1,",",A_C_AA$AD2,",",A_C_AA$GQ))

A_C_AA$AD<-as.character(A_C_AA$AD)

A_C_AA$AD1<-as.numeric(A_C_AA$AD1)

A_C_AA$AD2<-as.numeric(A_C_AA$AD2)

A_C_AA$GQ<-as.numeric(A_C_AA$GQ)

A_C_CC<-data.frame(A_C_CC,AD=paste(A_C_CC$AD1,",",A_C_CC$AD2,",",A_C_CC$GQ))

A_C_CC$AD<-as.character(A_C_CC$AD)

A_C_CC$AD1<-as.numeric(A_C_CC$AD1)

A_C_CC$AD2<-as.numeric(A_C_CC$AD2)

A_C_CC$GQ<-as.numeric(A_C_CC$GQ)

for( i in 1:length(A_C_AC$GQ)){

if(A_C_AC$GQ[i]<=20){A_C_AC$G_GQ[i]<-1}

if(A_C_AC$GQ[i]>20&&A_C_AC$GQ[i]<=40){A_C_AC$G_GQ[i]<-2}

if(A_C_AC$GQ[i]>40&&A_C_AC$GQ[i]<=60){A_C_AC$G_GQ[i]<-3}

if(A_C_AC$GQ[i]>60&&A_C_AC$GQ[i]<=80){A_C_AC$G_GQ[i]<-4}

if(A_C_AC$GQ[i]>80&&A_C_AC$GQ[i]<99){A_C_AC$G_GQ[i]<-5}

if(A_C_AC$GQ[i]>=99){A_C_AC$G_GQ[i]<-6}

}

f_GQ_A_C_AC<-table(A_C_AC$G_GQ)/length(A_C_AC$G_GQ)

for( i in 1:length(A_C_AA$GQ)){

if(A_C_AA$GQ[i]<=20){A_C_AA$G_GQ[i]<-1}

if(A_C_AA$GQ[i]>20&&A_C_AA$GQ[i]<=40){A_C_AA$G_GQ[i]<-2}

if(A_C_AA$GQ[i]>40&&A_C_AA$GQ[i]<=60){A_C_AA$G_GQ[i]<-3}

if(A_C_AA$GQ[i]>60&&A_C_AA$GQ[i]<=80){A_C_AA$G_GQ[i]<-4}

if(A_C_AA$GQ[i]>80&&A_C_AA$GQ[i]<99){A_C_AA$G_GQ[i]<-5}

if(A_C_AA$GQ[i]>=99){A_C_AA$G_GQ[i]<-6}

}

f_GQ_A_C_AA<-table(A_C_AA$G_GQ)/length(A_C_AA$G_GQ)

for( i in 1:length(A_C_CC$GQ)){

if(A_C_CC$GQ[i]<=20){A_C_CC$G_GQ[i]<-1}

if(A_C_CC$GQ[i]>20&&A_C_CC$GQ[i]<=40){A_C_CC$G_GQ[i]<-2}

if(A_C_CC$GQ[i]>40&&A_C_CC$GQ[i]<=60){A_C_CC$G_GQ[i]<-3}

if(A_C_CC$GQ[i]>60&&A_C_CC$GQ[i]<=80){A_C_CC$G_GQ[i]<-4}

if(A_C_CC$GQ[i]>80&&A_C_CC$GQ[i]<99){A_C_CC$G_GQ[i]<-5}

if(A_C_CC$GQ[i]>=99){A_C_CC$G_GQ[i]<-6}

}

f_GQ_A_C_CC<-table(A_C_CC$G_GQ)/length(A_C_CC$G_GQ)

b<-Sys.time()

print(b-a)

#############################################################################################################################################

########################## for every position #################################################################

#############################################################################################################################################

for(p in gt$pos[pa[1]:fa[1]]){

print(p)

a<-Sys.time()

gt_pos<-subset(gt,gt$pos==p)

gt_r<-gt_pos[,-(1:4)]

gt_r<-t(gt_r)

#numeric genotypes

GT_q<-gt_r

for(i in 1:length(gt_r)){

if(gt_r[i]=="TT" & gt_pos$alleles=="G/T"){GT_q[i]=2}

if(gt_r[i]=="GT" & gt_pos$alleles=="G/T"){GT_q[i]=1}

if(gt_r[i]=="GG" & gt_pos$alleles=="G/T"){GT_q[i]=0}

if(gt_r[i]=="TT" & gt_pos$alleles=="C/T"){GT_q[i]=2}

if(gt_r[i]=="CT" & gt_pos$alleles=="C/T"){GT_q[i]=1}

if(gt_r[i]=="CC" & gt_pos$alleles=="C/T"){GT_q[i]=0}

if(gt_r[i]=="GG" & gt_pos$alleles=="C/G"){GT_q[i]=2}

if(gt_r[i]=="CG" & gt_pos$alleles=="C/G"){GT_q[i]=1}

if(gt_r[i]=="CC" & gt_pos$alleles=="C/G"){GT_q[i]=0}

if(gt_r[i]=="TT" & gt_pos$alleles=="A/T"){GT_q[i]=2}

if(gt_r[i]=="AT" & gt_pos$alleles=="A/T"){GT_q[i]=1}

if(gt_r[i]=="AA" & gt_pos$alleles=="A/T"){GT_q[i]=0}

if(gt_r[i]=="GG" & gt_pos$alleles=="A/G"){GT_q[i]=2}

if(gt_r[i]=="AG" & gt_pos$alleles=="A/G"){GT_q[i]=1}

if(gt_r[i]=="AA" & gt_pos$alleles=="A/G"){GT_q[i]=0}

if(gt_r[i]=="CC" & gt_pos$alleles=="A/C"){GT_q[i]=2}

if(gt_r[i]=="AC" & gt_pos$alleles=="A/C"){GT_q[i]=1}

if(gt_r[i]=="AA" & gt_pos$alleles=="A/C"){GT_q[i]=0}

}

GT_q1<-as.numeric(GT_q)

names(GT_q1)<-row.names(GT_q)

GT_q<-GT_q1

#phenotypes quantitative

Ph<-GT_q

Phq<-rep(0,length(Ph))

for(i in 1:length(GT_q)){

Phq[i]<-rnorm(1,GT_q[i],6.5)

}

data_1<-data.frame(GT_real=GT_q,Phq)

##########################################################################################################################

#phenotypes categorical

Phd<-GT_q

for(i in 1:length(GT_q)){

if(is.na(Phq[i])==T){Phd[i]<-NA}

if(is.na(Phq[i])==F && Phq[i]<=median(Phq,na.rm=T) ){Phd[i]<-0}

if(is.na(Phq[i])==F && Phq[i]>median(Phq,na.rm=T) ){Phd[i]<-1}

}

#data frame: GT,Ph1,Ph2

data_1<-data.frame(GT_real=GT_q,Phq=Phq,Phd=Phd)

#write.table(data_1,"data_1.txt")

# AD and GQ

ad_sim<-data.frame(AD1=rep(0,1417),AD2=0,GQ=0)

rownames(ad_sim)<-colnames(gt[5:1421])

for (j in 5:length(gt_pos)){

for(i in 1:length(gt_pos$rs)){

if(gt_pos[i,]$alleles == "C/T" & gt_pos[i,j]=="CT"){

#choose gq group

group<-sample(c(1,2,3,4,5,6),1,replace=T,prob=f_GQ_C_T_CT)

#choose a gp randomly for that group

test<-subset(C_T_CT,C_T_CT$G_GQ==group)[,c(5,6,9)]

n<-sample(rownames(test),1)

ad_sim[j-4,1:3]<-test[n,]

}

if(gt_pos[i,]$alleles == "C/T" & gt_pos[i,j]=="TT"){

#choose gq group

group<-sample(c(1,2,3,4,5,6),1,replace=T,prob=f_GQ_C_T_TT)

#choose a gp randomly for that group

test<-subset(C_T_TT,C_T_TT$G_GQ==group)[,c(5,6,9)]

n<-sample(rownames(test),1)

ad_sim[j-4,1:3]<-test[n,]

}

if(gt_pos[i,]$alleles == "C/T" & gt_pos[i,j]=="CC"){

#choose gq group

group<-sample(c(1,2,3,4,5,6),1,replace=T,prob=f_GQ_C_T_CC)

#choose a gp randomly for that group

test<-subset(C_T_CC,C_T_CC$G_GQ==group)[,c(5,6,9)]

n<-sample(rownames(test),1)

ad_sim[j-4,1:3]<-test[n,]

}

if(gt_pos[i,]$alleles == "G/T" & gt_pos[i,j]=="GT"){

#choose gq group

group<-sample(c(1,2,3,4,5,6),1,replace=T,prob=f_GQ_G_T_GT)

#choose a gp randomly for that group

test<-subset(G_T_GT,G_T_GT$G_GQ==group)[,c(5,6,9)]

n<-sample(rownames(test),1)

ad_sim[j-4,1:3]<-test[n,]

}

if(gt_pos[i,]$alleles == "G/T" & gt_pos[i,j]=="TT"){

#choose gq group

group<-sample(c(1,2,3,4,5,6),1,replace=T,prob=f_GQ_G_T_TT)

#choose a gp randomly for that group

test<-subset(G_T_TT,G_T_TT$G_GQ==group)[,c(5,6,9)]

n<-sample(rownames(test),1)

ad_sim[j-4,1:3]<-test[n,]

}

if(gt_pos[i,]$alleles == "G/T" & gt_pos[i,j]=="GG"){

#choose gq group

group<-sample(c(1,2,3,4,5,6),1,replace=T,prob=f_GQ_G_T_GG)

#choose a gp randomly for that group

test<-subset(G_T_GG,G_T_GG$G_GQ==group)[,c(5,6,9)]

n<-sample(rownames(test),1)

ad_sim[j-4,1:3]<-test[n,]

}

if(gt_pos[i,]$alleles == "C/G" & gt_pos[i,j]=="CG"){

#choose gq group

group<-sample(c(1,2,3,4,5,6),1,replace=T,prob=f_GQ_C_G_CG)

#choose a gp randomly for that group

test<-subset(C_G_CG,C_G_CG$G_GQ==group)[,c(5,6,9)]

n<-sample(rownames(test),1)

ad_sim[j-4,1:3]<-test[n,]

}

if(gt_pos[i,]$alleles == "C/G" & gt_pos[i,j]=="GG"){

#choose gq group

group<-sample(c(1,2,3,4,5,6),1,replace=T,prob=f_GQ_C_G_GG)

#choose a gp randomly for that group

test<-subset(C_G_GG,C_G_GG$G_GQ==group)[,c(5,6,9)]

n<-sample(rownames(test),1)

ad_sim[j-4,1:3]<-test[n,]

}

if(gt_pos[i,]$alleles == "C/G" & gt_pos[i,j]=="CC"){

#choose gq group

group<-sample(c(1,2,3,4,5,6),1,replace=T,prob=f_GQ_C_G_CC)

#choose a gp randomly for that group

test<-subset(C_G_CC,C_G_CC$G_GQ==group)[,c(5,6,9)]

n<-sample(rownames(test),1)

ad_sim[j-4,1:3]<-test[n,]

}

if(gt_pos[i,]$alleles == "A/T" & gt_pos[i,j]=="AT"){

#choose gq group

group<-sample(c(1,2,3,4,5,6),1,replace=T,prob=f_GQ_A_T_AT)

#choose a gp randomly for that group

test<-subset(A_T_AT,A_T_AT$G_GQ==group)[,c(5,6,9)]

n<-sample(rownames(test),1)

ad_sim[j-4,1:3]<-test[n,]

}

if(gt_pos[i,]$alleles == "A/T" & gt_pos[i,j]=="TT"){

#choose gq group

group<-sample(c(1,2,3,4,5,6),1,replace=T,prob=f_GQ_A_T_TT)

#choose a gp randomly for that group

test<-subset(A_T_TT,A_T_TT$G_GQ==group)[,c(5,6,9)]

n<-sample(rownames(test),1)

ad_sim[j-4,1:3]<-test[n,]

}

if(gt_pos[i,]$alleles == "A/T" & gt_pos[i,j]=="AA"){

#choose gq group

group<-sample(c(1,2,3,4,5,6),1,replace=T,prob=f_GQ_A_T_AA)

#choose a gp randomly for that group

test<-subset(A_T_AA,A_T_AA$G_GQ==group)[,c(5,6,9)]

n<-sample(rownames(test),1)

ad_sim[j-4,1:3]<-test[n,]

}

if(gt_pos[i,]$alleles == "A/G" & gt_pos[i,j]=="AG"){

#choose gq group

group<-sample(c(1,2,3,4,5,6),1,replace=T,prob=f_GQ_A_G_AG)

#choose a gp randomly for that group

test<-subset(A_G_AG,A_G_AG$G_GQ==group)[,c(5,6,9)]

n<-sample(rownames(test),1)

ad_sim[j-4,1:3]<-test[n,]

}

if(gt_pos[i,]$alleles == "A/G" & gt_pos[i,j]=="GG"){

#choose gq group

group<-sample(c(1,2,3,4,5,6),1,replace=T,prob=f_GQ_A_G_GG)

#choose a gp randomly for that group

test<-subset(A_G_GG,A_G_GG$G_GQ==group)[,c(5,6,9)]

n<-sample(rownames(test),1)

ad_sim[j-4,1:3]<-test[n,]

}

if(gt_pos[i,]$alleles == "A/G" & gt_pos[i,j]=="AA"){

#choose gq group

group<-sample(c(1,2,3,4,5,6),1,replace=T,prob=f_GQ_A_G_AA)

#choose a gp randomly for that group

test<-subset(A_G_AA,A_G_AA$G_GQ==group)[,c(5,6,9)]

n<-sample(rownames(test),1)

ad_sim[j-4,1:3]<-test[n,]

}

if(gt_pos[i,]$alleles == "A/C" & gt_pos[i,j]=="AC"){

#choose gq group

group<-sample(c(1,2,3,4,5,6),1,replace=T,prob=f_GQ_A_C_AC)

#choose a gp randomly for that group

test<-subset(A_C_AC,A_C_AC$G_GQ==group)[,c(5,6,9)]

n<-sample(rownames(test),1)

ad_sim[j-4,1:3]<-test[n,]

}

if(gt_pos[i,]$alleles == "A/C" & gt_pos[i,j]=="CC"){

#choose gq group

group<-sample(c(1,2,3,4,5,6),1,replace=T,prob=f_GQ_A_C_CC)

#choose a gp randomly for that group

test<-subset(A_C_CC,A_C_CC$G_GQ==group)[,c(5,6,9)]

n<-sample(rownames(test),1)

ad_sim[j-4,1:3]<-test[n,]

}

if(gt_pos[i,]$alleles == "A/C" & gt_pos[i,j]=="AA"){

#choose gq group

group<-sample(c(1,2,3,4,5,6),1,replace=T,prob=f_GQ_A_C_AA)

#choose a gp randomly for that group

test<-subset(A_C_AA,A_C_AA$G_GQ==group)[,c(5,6,9)]

n<-sample(rownames(test),1)

ad_sim[j-4,1:3]<-test[n,]

}

}}

# ad[v]<-ad_sim

# print(k)

#} #fin k --- sim GQ AD1 AD2

data_2<-data.frame(GT_real=GT_q,ad_sim)

#names_ad<-paste(c("AD1","AD2","GQ"),rep(1:100, each=3),sep="")

#colnames(data_2)<-c("GT_real",names_ad)

#simulate 100 data set mixed

data_3<-data.frame(GT_real=data_1$GT_real,Phq=data_1$Phq,Phd=data_1$Phd,GQ=data_2$GQ,AD1=data_2$AD1,AD2=data_2$AD2,GT_estim=estim_gt(data_2[,-1])$GT)

data_3$Phq<-as.numeric(data_3$Phq)

data<-data.frame(data_3,S_AD=data_3$AD1+data_3$AD2)

#remove individual with 0,0 AD

data<-subset(data, data$S_AD!=0)

#GT in factor and group

for( i in 1:length(data$GT_estim)){

if(data$GT_estim[i]=="0/0"){data$GT_estim_g[i]<-0}

if(data$GT_estim[i]=="0/1"){data$GT_estim_g[i]<-1}

if(data$GT_estim[i]=="1/1"){data$GT_estim_g[i]<-2}

}

data$GT_estim_g<-as.factor(data$GT_estim_g)

data$GT_estim<-as.factor(data$GT_estim)

data$GT_real<-as.factor(data$GT_real)

data<-data.frame(data,ratio=data$AD2/data$S_AD)

data<-cbind(data,GT_realn=as.numeric(as.character(data$GT_real)))

data<-cbind(data,GT_estim_gn=as.numeric(as.character(data$GT_estim_g)))

# write.table(data_3,"data_3.txt")

#save contingency table

r0e0<-length(subset(data,data$GT_real==0 & data$GT_estim_g==0)[,1])

r0e1<-length(subset(data,data$GT_real==0 & data$GT_estim_g==1)[,1])

r0e2<-length(subset(data,data$GT_real==0 & data$GT_estim_g==2)[,1])

r1e0<-length(subset(data,data$GT_real==1 & data$GT_estim_g==0)[,1])

r1e1<-length(subset(data,data$GT_real==1 & data$GT_estim_g==1)[,1])

r1e2<-length(subset(data,data$GT_real==1 & data$GT_estim_g==2)[,1])

r2e0<-length(subset(data,data$GT_real==2 & data$GT_estim_g==0)[,1])

r2e1<-length(subset(data,data$GT_real==2 & data$GT_estim_g==1)[,1])

r2e2<-length(subset(data,data$GT_real==2 & data$GT_estim_g==2)[,1])

#save

medianGQ<-median(data$GQ)

medianSAD<-median(data$S_AD)

print("fitting models")

#######################################################################################################################

#################################### AD2~Phq ###############################################################

#######################################################################################################################

#standard poisson model AD2~Phq

cc <- try(glm(AD2~Phq, family=poisson,data=data, offset=log(S_AD)), silent=T)

if(is(cc,"try-error")) {ad2_ph_poisson<-NA

e_ad2_ph_poisson_beta0<-NA

e_ad2_ph_poisson_beta0_se<-NA

e_ad2_ph_poisson_beta1<-NA

e_ad2_ph_poisson_beta1_se<-NA}

if(is(cc,"try-error")==F) {ad2_ph_poisson<-summary(glm(AD2~Phq, family=poisson,data=data, offset=log(S_AD)))$coefficients[2,4]

e_ad2_ph_poisson_beta0<-summary(glm(AD2~Phq, family=poisson,data=data, offset=log(S_AD)))$coefficients[1,1]

e_ad2_ph_poisson_beta0_se<-summary(glm(AD2~Phq, family=poisson,data=data, offset=log(S_AD)))$coefficients[1,2]

e_ad2_ph_poisson_beta1<-summary(glm(AD2~Phq, family=poisson,data=data, offset=log(S_AD)))$coefficients[2,1]

e_ad2_ph_poisson_beta1_se<-summary(glm(AD2~Phq, family=poisson,data=data, offset=log(S_AD)))$coefficients[2,2]}

#negative binomial AD2~Phq

cc <- try(glm.nb(AD2~Phq+offset(log(S_AD)), data = data), silent=T)

if(is(cc,"try-error")) { ad2_ph_nb<-NA

e_ad2_ph_nb_beta0<-NA

e_ad2_ph_nb_beta0_se<-NA

e_ad2_ph_nb_beta1<-NA

e_ad2_ph_nb_beta1_se<-NA}

if(is(cc,"try-error")==F) { ad2_ph_nb<-summary(glm.nb(AD2~Phq+offset(log(S_AD)), data = data))$coefficients[2,4]

e_ad2_ph_nb_beta0<-summary(glm.nb(AD2~Phq+offset(log(S_AD)), data = data))$coefficients[1,1]

e_ad2_ph_nb_beta0_se<-summary(glm.nb(AD2~Phq+offset(log(S_AD)), data = data))$coefficients[1,2]

e_ad2_ph_nb_beta1<-summary(glm.nb(AD2~Phq+offset(log(S_AD)), data = data))$coefficients[2,1]

e_ad2_ph_nb_beta1_se<-summary(glm.nb(AD2~Phq+offset(log(S_AD)), data = data))$coefficients[2,2]}

#zero inflated negative binomial AD2~Phq

cc <- try(zeroinfl(AD2~Phq,data=data,offset=log(S_AD),dist = "negbin"), silent=T)

xx <- try(zeroinfl(AD2~1, data=data,offset=log(S_AD),dist = "negbin"),silent=T)

if(is(cc,"try-error")) {ad2_ph_nb_zi<-NA

e_ad2_ph_nb_zi_beta0c<-NA

e_ad2_ph_nb_zi_beta0c_se<-NA

e_ad2_ph_nb_zi_beta1c<-NA

e_ad2_ph_nb_zi_beta1c_se<-NA

e_ad2_ph_nb_zi_logthetac<-NA

e_ad2_ph_nb_zi_logthetac_se<-NA

e_ad2_ph_nb_zi_beta0z<-NA

e_ad2_ph_nb_zi_beta0z_se<-NA

e_ad2_ph_nb_zi_beta1z<-NA

e_ad2_ph_nb_zi_beta1z_se<-NA}

if(is(xx,"try-error")) {ad2_ph_nb_zi<-NA

e_ad2_ph_nb_zi_beta0c<-NA

e_ad2_ph_nb_zi_beta0c_se<-NA

e_ad2_ph_nb_zi_beta1c<-NA

e_ad2_ph_nb_zi_beta1c_se<-NA

e_ad2_ph_nb_zi_logthetac<-NA

e_ad2_ph_nb_zi_logthetac_se<-NA

e_ad2_ph_nb_zi_beta0z<-NA

e_ad2_ph_nb_zi_beta0z_se<-NA

e_ad2_ph_nb_zi_beta1z<-NA

e_ad2_ph_nb_zi_beta1z_se<-NA}

if(is(cc,"try-error")==F & is(xx,"try-error")==F) { ad2_ph_nb_zi<-1-pchisq(-2*(zeroinfl(AD2~1,data=data,offset=log(S_AD),dist = "negbin")$loglik-zeroinfl(AD2~Phq,data=data,offset=log(S_AD),dist = "negbin")$loglik),2)

e_ad2_ph_nb_zi_beta0c<-summary(zeroinfl(AD2~Phq,data=data,offset=log(S_AD),dist = "negbin"))$coefficients$count[1,1]

e_ad2_ph_nb_zi_beta0c_se<-summary(zeroinfl(AD2~Phq,data=data,offset=log(S_AD),dist = "negbin"))$coefficients$count[1,2]

e_ad2_ph_nb_zi_beta1c<-summary(zeroinfl(AD2~Phq,data=data,offset=log(S_AD),dist = "negbin"))$coefficients$count[2,1]

e_ad2_ph_nb_zi_beta1c_se<-summary(zeroinfl(AD2~Phq,data=data,offset=log(S_AD),dist = "negbin"))$coefficients$count[2,2]

e_ad2_ph_nb_zi_logthetac<-summary(zeroinfl(AD2~Phq,data=data,offset=log(S_AD),dist = "negbin"))$coefficients$count[3,1]

e_ad2_ph_nb_zi_logthetac_se<-summary(zeroinfl(AD2~Phq,data=data,offset=log(S_AD),dist = "negbin"))$coefficients$count[3,2]

e_ad2_ph_nb_zi_beta0z<-summary(zeroinfl(AD2~Phq,data=data,offset=log(S_AD),dist = "negbin"))$coefficients$zero[1,1]

e_ad2_ph_nb_zi_beta0z_se<-summary(zeroinfl(AD2~Phq,data=data,offset=log(S_AD),dist = "negbin"))$coefficients$zero[1,2]

e_ad2_ph_nb_zi_beta1z<-summary(zeroinfl(AD2~Phq,data=data,offset=log(S_AD),dist = "negbin"))$coefficients$zero[2,1]

e_ad2_ph_nb_zi_beta1z_se<-summary(zeroinfl(AD2~Phq,data=data,offset=log(S_AD),dist = "negbin"))$coefficients$zero[2,2]}

#hurdle negative binomial AD2~Phq

cc <- try(hurdle(AD2~Phq, data=data,offset=log(S_AD),dist = "negbin"), silent=T)

xx <- try(hurdle(AD2~1, data=data,offset=log(S_AD),dist = "negbin"),silent=T)

if(is(cc,"try-error")) {ad2_ph_nb_hur<-NA

e_ad2_ph_nb_hur_beta0c<-NA

e_ad2_ph_nb_hur_beta0c_se<-NA

e_ad2_ph_nb_hur_beta1c<-NA

e_ad2_ph_nb_hur_beta1c_se<-NA

e_ad2_ph_nb_hur_logthetac<-NA

e_ad2_ph_nb_hur_logthetac_se<-NA

e_ad2_ph_nb_hur_beta0z<-NA

e_ad2_ph_nb_hur_beta0z_se<-NA

e_ad2_ph_nb_hur_beta1z<-NA

e_ad2_ph_nb_hur_beta1z_se<-NA }

if(is(xx,"try-error")) {ad2_ph_nb_hur<-NA

e_ad2_ph_nb_hur_beta0c<-NA

e_ad2_ph_nb_hur_beta0c_se<-NA

e_ad2_ph_nb_hur_beta1c<-NA

e_ad2_ph_nb_hur_beta1c_se<-NA

e_ad2_ph_nb_hur_logthetac<-NA

e_ad2_ph_nb_hur_logthetac_se<-NA

e_ad2_ph_nb_hur_beta0z<-NA

e_ad2_ph_nb_hur_beta0z_se<-NA

e_ad2_ph_nb_hur_beta1z<-NA

e_ad2_ph_nb_hur_beta1z_se<-NA }

if(is(cc,"try-error")==F & is(xx,"try-error")==F) { ad2_ph_nb_hur<-1-pchisq(-2*(hurdle(AD2~1, data=data,offset=log(S_AD),dist = "negbin")$loglik-hurdle(AD2~Phq, data=data,offset=log(S_AD),dist = "negbin")$loglik),2)

e_ad2_ph_nb_hur_beta0c<-summary(hurdle(AD2~Phq, data=data,offset=log(S_AD),dist = "negbin"))$coefficients$count[1,1]

e_ad2_ph_nb_hur_beta0c_se<-summary(hurdle(AD2~Phq, data=data,offset=log(S_AD),dist = "negbin"))$coefficients$count[1,2]

e_ad2_ph_nb_hur_beta1c<-summary(hurdle(AD2~Phq, data=data,offset=log(S_AD),dist = "negbin"))$coefficients$count[2,1]

e_ad2_ph_nb_hur_beta1c_se<-summary(hurdle(AD2~Phq, data=data,offset=log(S_AD),dist = "negbin"))$coefficients$count[2,2]

e_ad2_ph_nb_hur_logthetac<-summary(hurdle(AD2~Phq, data=data,offset=log(S_AD),dist = "negbin"))$coefficients$count[3,1]

e_ad2_ph_nb_hur_logthetac_se<-summary(hurdle(AD2~Phq, data=data,offset=log(S_AD),dist = "negbin"))$coefficients$count[3,2]

e_ad2_ph_nb_hur_beta0z<-summary(hurdle(AD2~Phq, data=data,offset=log(S_AD),dist = "negbin"))$coefficients$zero[1,1]

e_ad2_ph_nb_hur_beta0z_se<-summary(hurdle(AD2~Phq, data=data,offset=log(S_AD),dist = "negbin"))$coefficients$zero[1,2]

e_ad2_ph_nb_hur_beta1z<-summary(hurdle(AD2~Phq, data=data,offset=log(S_AD),dist = "negbin"))$coefficients$zero[2,1]

e_ad2_ph_nb_hur_beta1z_se<-summary(hurdle(AD2~Phq, data=data,offset=log(S_AD),dist = "negbin"))$coefficients$zero[2,2] }

#######################################################################################################################

#################################### ratio~Phq ###############################################################

#######################################################################################################################

#standard linear model Phq~AD2

cc <- try(lm(ratio~Phq, data = data), silent=T)

xx<- try(summary(lm(ratio~Phq, data = data)), silent=T)

if(is(cc,"try-error")) { ratio_ph_lm<-NA

e_ratio_ph_lm_beta0<-NA

e_ratio_ph_lm_beta0_se<-NA

e_ratio_ph_lm_beta1<-NA

e_ratio_ph_lm_beta1_se<-NA}

if(is(xx,"try-error")) { ratio_ph_lm<-NA

e_ratio_ph_lm_beta0<-NA

e_ratio_ph_lm_beta0_se<-NA

e_ratio_ph_lm_beta1<-NA

e_ratio_ph_lm_beta1_se<-NA}

if(is(cc,"try-error")==F & is(xx,"try-error")==F) { ratio_ph_lm<-summary(lm(ratio~Phq, data = data))$coefficients[2,4]

e_ratio_ph_lm_beta0<-summary(lm(ratio~Phq, data = data))$coefficients[1,1]

e_ratio_ph_lm_beta0_se<-summary(lm(ratio~Phq, data = data))$coefficients[1,2]

e_ratio_ph_lm_beta1<-summary(lm(ratio~Phq, data = data))$coefficients[2,1]

e_ratio_ph_lm_beta1_se<-summary(lm(ratio~Phq, data = data))$coefficients[2,2]}

#######################################################################################################################

#################################### Phq~ratio ###############################################################

#######################################################################################################################

#standard linear model Phq~AD2

cc <- try(lm(Phq~ratio, data = data), silent=T)

xx<- try(summary(lm(Phq~ratio, data = data)), silent=T)

if(is(cc,"try-error")) { ph_ratio_lm<-NA

e_ph_ratio_lm_beta0<-NA

e_ph_ratio_lm_beta0_se<-NA

e_ph_ratio_lm_beta1<-NA

e_ph_ratio_lm_beta1_se<-NA}

if(is(xx,"try-error")) { ph_ratio_lm<-NA

e_ph_ratio_lm_beta0<-NA

e_ph_ratio_lm_beta0_se<-NA

e_ph_ratio_lm_beta1<-NA

e_ph_ratio_lm_beta1_se<-NA}

if(is(cc,"try-error")==F & is(xx,"try-error")==F) { ph_ratio_lm<-summary(lm(Phq~ratio, data = data))$coefficients[2,4]

e_ph_ratio_lm_beta0<-summary(lm(Phq~ratio, data = data))$coefficients[1,1]

e_ph_ratio_lm_beta0_se<-summary(lm(Phq~ratio, data = data))$coefficients[1,2]

e_ph_ratio_lm_beta1<-summary(lm(Phq~ratio, data = data))$coefficients[2,1]

e_ph_ratio_lm_beta1_se<-summary(lm(Phq~ratio, data = data))$coefficients[2,2]}

#######################################################################################################################

#################################### GT_estim~Phq ############################################################

#######################################################################################################################

#ordinal logistic standard model GT_estim~Phq

cc <- try(polr(GT_estim~Phq, data = data, Hess = TRUE), silent=T)

if(is(cc,"try-error")) {gt_ph_ordinal_logit<-NA

e_gt_ph_ordinal_logit_coef<-NA

e_gt_ph_ordinal_logit_coef_se<-NA

e_gt_ph_ordinal_logit_int1<-NA

e_gt_ph_ordinal_logit_int1_se<-NA

e_gt_ph_ordinal_logit_int2<-NA

e_gt_ph_ordinal_logit_int2_se<-NA}

if(is(cc,"try-error")==F) {gt_ph_ordinal_logit<-pnorm(abs(summary( polr(GT_estim~Phq, data = data, Hess = TRUE) )$coefficients[1,3]),lower.tail=F)*2

e_gt_ph_ordinal_logit_coef<-summary( polr(GT_estim~Phq, data = data, Hess = TRUE) )$coefficients[1,1]

e_gt_ph_ordinal_logit_coef_se<-summary( polr(GT_estim~Phq, data = data, Hess = TRUE) )$coefficients[1,2]

e_gt_ph_ordinal_logit_int1<-summary( polr(GT_estim~Phq, data = data, Hess = TRUE) )$coefficients[2,1]

e_gt_ph_ordinal_logit_int1_se<-summary( polr(GT_estim~Phq, data = data, Hess = TRUE) )$coefficients[2,2]

e_gt_ph_ordinal_logit_int2<-summary( polr(GT_estim~Phq, data = data, Hess = TRUE) )$coefficients[3,1]

e_gt_ph_ordinal_logit_int2_se<-summary( polr(GT_estim~Phq, data = data, Hess = TRUE) )$coefficients[3,2]}

#logistic regression standard GT_estim_g~Phq

cc <- try(glm(GT_estim_g~Phq, family=binomial(link=logit),data=data), silent=T)

xx<- try(summary(glm(GT_estim_g~Phq, family=binomial(link=logit),data=data), silent=T))

if(is(cc,"try-error")) { gt_ph_logit<-NA

e_gt_ph_logit_beta0<-NA

e_gt_ph_logit_beta0_se<-NA

e_gt_ph_logit_beta1<-NA

e_gt_ph_logit_beta1_se<-NA}

if(is(xx,"try-error")) { gt_ph_logit<-NA

e_gt_ph_logit_beta0<-NA

e_gt_ph_logit_beta0_se<-NA

e_gt_ph_logit_beta1<-NA

e_gt_ph_logit_beta1_se<-NA}

if(is(cc,"try-error")==F & is(xx,"try-error")==F) { gt_ph_logit<-summary(glm(GT_estim_g~Phq, family=binomial(link=logit),data=data))$coefficients[2,4]

e_gt_ph_logit_beta0<-summary(glm(GT_estim_g~Phq, family=binomial(link=logit),data=data))$coefficients[1,1]

e_gt_ph_logit_beta0_se<-summary(glm(GT_estim_g~Phq, family=binomial(link=logit),data=data))$coefficients[1,2]

e_gt_ph_logit_beta1<-summary(glm(GT_estim_g~Phq, family=binomial(link=logit),data=data))$coefficients[2,1]

e_gt_ph_logit_beta1_se<-summary(glm(GT_estim_g~Phq, family=binomial(link=logit),data=data))$coefficients[2,2]}

#######################################################################################################################

#################################### Phq~GT_estim ###########################################################

#######################################################################################################################

#standard linear model Phq~GT_estim_gn

cc <- try(lm(Phq~GT_estim_gn , data = data), silent=T)

xx<- try(summary(lm(Phq~GT_estim_gn , data = data)), silent=T)

if(is(cc,"try-error")) { ph_gt_lm<-NA

e_ph_gt_lm_beta0<-NA

e_ph_gt_lm_beta0_se<-NA

e_ph_gt_lm_beta1<-NA

e_ph_gt_lm_beta1_se<-NA}

if(is(xx,"try-error")) { ph_gt_lm<-NA

e_ph_gt_lm_beta0<-NA

e_ph_gt_lm_beta0_se<-NA

e_ph_gt_lm_beta1<-NA

e_ph_gt_lm_beta1_se<-NA}

if(is(cc,"try-error")==F & is(xx,"try-error")==F) { ph_gt_lm<-summary(lm(Phq~GT_estim_gn , data = data))$coefficients[2,4]

e_ph_gt_lm_beta0<-summary(lm(Phq~GT_estim_gn , data = data))$coefficients[1,1]

e_ph_gt_lm_beta0_se<-summary(lm(Phq~GT_estim_gn , data = data))$coefficients[1,2]

e_ph_gt_lm_beta1<-summary(lm(Phq~GT_estim_gn , data = data))$coefficients[2,1]

e_ph_gt_lm_beta1_se<-summary(lm(Phq~GT_estim_gn , data = data))$coefficients[2,2]}

#######################################################################################################################

#################################### GT_real~Phq ############################################################

#######################################################################################################################

#ordinal logistic standard model GT_real~Phq

cc <- try(polr(GT_real~Phq, data = data, Hess = TRUE), silent=T)

if(is(cc,"try-error")) {gtr_ph_ordinal_logit<-NA

e_gtr_ph_ordinal_logit_coef<-NA

e_gtr_ph_ordinal_logit_coef_se<-NA

e_gtr_ph_ordinal_logit_int1<-NA

e_gtr_ph_ordinal_logit_int1_se<-NA

e_gtr_ph_ordinal_logit_int2<-NA

e_gtr_ph_ordinal_logit_int2_se<-NA}

if(is(cc,"try-error")==F) {gtr_ph_ordinal_logit<-pnorm(abs(summary( polr(GT_real~Phq, data = data, Hess = TRUE) )$coefficients[1,3]),lower.tail=F)*2

e_gtr_ph_ordinal_logit_coef<-summary( polr(GT_real~Phq, data = data, Hess = TRUE) )$coefficients[1,1]

e_gtr_ph_ordinal_logit_coef_se<-summary( polr(GT_real~Phq, data = data, Hess = TRUE) )$coefficients[1,2]

e_gtr_ph_ordinal_logit_int1<-summary( polr(GT_real~Phq, data = data, Hess = TRUE) )$coefficients[2,1]

e_gtr_ph_ordinal_logit_int1_se<-summary( polr(GT_real~Phq, data = data, Hess = TRUE) )$coefficients[2,2]

e_gtr_ph_ordinal_logit_int2<-summary( polr(GT_real~Phq, data = data, Hess = TRUE) )$coefficients[3,1]

e_gtr_ph_ordinal_logit_int2_se<-summary( polr(GT_real~Phq, data = data, Hess = TRUE) )$coefficients[3,2]}

#logistic regression standard GT_real~Phq

cc <- try(glm(GT_real~Phq, family=binomial(link=logit),data=data), silent=T)

xx<- try(summary(glm(GT_real~Phq, family=binomial(link=logit),data=data), silent=T))

if(is(cc,"try-error")) { gtr_ph_logit<-NA

e_gtr_ph_logit_beta0<-NA

e_gtr_ph_logit_beta0_se<-NA

e_gtr_ph_logit_beta1<-NA

e_gtr_ph_logit_beta1_se<-NA}

if(is(xx,"try-error")) { gtr_ph_logit<-NA

e_gtr_ph_logit_beta0<-NA

e_gtr_ph_logit_beta0_se<-NA

e_gtr_ph_logit_beta1<-NA

e_gtr_ph_logit_beta1_se<-NA}

if(is(cc,"try-error")==F & is(xx,"try-error")==F) { gtr_ph_logit<-summary(glm(GT_real~Phq, family=binomial(link=logit),data=data))$coefficients[2,4]

e_gtr_ph_logit_beta0<-summary(glm(GT_real~Phq, family=binomial(link=logit),data=data))$coefficients[1,1]

e_gtr_ph_logit_beta0_se<-summary(glm(GT_real~Phq, family=binomial(link=logit),data=data))$coefficients[1,2]

e_gtr_ph_logit_beta1<-summary(glm(GT_real~Phq, family=binomial(link=logit),data=data))$coefficients[2,1]

e_gtr_ph_logit_beta1_se<-summary(glm(GT_real~Phq, family=binomial(link=logit),data=data))$coefficients[2,2]}

#######################################################################################################################

#################################### Phq~GT_real ###########################################################

#######################################################################################################################

#standard linear model Phq~GT_realn

cc <- try(lm(Phq~GT_realn , data = data), silent=T)

xx<- try(summary(lm(Phq~GT_realn , data = data)), silent=T)

if(is(cc,"try-error")) { ph_gtr_lm<-NA

e_ph_gtr_lm_beta0<-NA

e_ph_gtr_lm_beta0_se<-NA

e_ph_gtr_lm_beta1<-NA

e_ph_gtr_lm_beta1_se<-NA}

if(is(xx,"try-error")) { ph_gtr_lm<-NA

e_ph_gtr_lm_beta0<-NA

e_ph_gtr_lm_beta0_se<-NA

e_ph_gtr_lm_beta1<-NA

e_ph_gtr_lm_beta1_se<-NA}

if(is(cc,"try-error")==F & is(xx,"try-error")==F) { ph_gtr_lm<-summary(lm(Phq~GT_realn , data = data))$coefficients[2,4]

e_ph_gtr_lm_beta0<-summary(lm(Phq~GT_realn , data = data))$coefficients[1,1]

e_ph_gtr_lm_beta0_se<-summary(lm(Phq~GT_realn , data = data))$coefficients[1,2]

e_ph_gtr_lm_beta1<-summary(lm(Phq~GT_realn , data = data))$coefficients[2,1]

e_ph_gtr_lm_beta1_se<-summary(lm(Phq~GT_realn , data = data))$coefficients[2,2]}

##################################################################################################################

##################################################################################################################

##################################################################################################################

##################################################################################################################

##################################################################################################################

##################################################################################################################

##################################################################################################################

##################################################################################################################

##################################################################################################################

###################################### PHENOTYPE CATEGORICAL ############################################

##################################################################################################################

##################################################################################################################

##################################################################################################################

##################################################################################################################

##################################################################################################################

##################################################################################################################

##################################################################################################################

##################################################################################################################

##################################################################################################################

##################################################################################################################

##################################################################################################################

##################################################################################################################

#######################################################################################################################

#################################### AD2~Phd ###############################################################

#######################################################################################################################

#standard poisson model AD2~Phd

cc <- try(glm(AD2~Phd, family=poisson,data=data, offset=log(S_AD)), silent=T)

if(is(cc,"try-error")) {ad2_phd_poisson<-NA

e_ad2_phd_poisson_beta0<-NA

e_ad2_phd_poisson_beta0_se<-NA

e_ad2_phd_poisson_beta1<-NA

e_ad2_phd_poisson_beta1_se<-NA}

if(is(cc,"try-error")==F) {ad2_phd_poisson<-summary(glm(AD2~Phd, family=poisson,data=data, offset=log(S_AD)))$coefficients[2,4]

e_ad2_phd_poisson_beta0<-summary(glm(AD2~Phd, family=poisson,data=data, offset=log(S_AD)))$coefficients[1,1]

e_ad2_phd_poisson_beta0_se<-summary(glm(AD2~Phd, family=poisson,data=data, offset=log(S_AD)))$coefficients[1,2]

e_ad2_phd_poisson_beta1<-summary(glm(AD2~Phd, family=poisson,data=data, offset=log(S_AD)))$coefficients[2,1]

e_ad2_phd_poisson_beta1_se<-summary(glm(AD2~Phd, family=poisson,data=data, offset=log(S_AD)))$coefficients[2,2]}

#negative binomial AD2~Phd

cc <- try(glm.nb(AD2~Phd+offset(log(S_AD)), data = data), silent=T)

if(is(cc,"try-error")) { ad2_phd_nb<-NA

e_ad2_phd_nb_beta0<-NA

e_ad2_phd_nb_beta0_se<-NA

e_ad2_phd_nb_beta1<-NA

e_ad2_phd_nb_beta1_se<-NA}

if(is(cc,"try-error")==F) { ad2_phd_nb<-summary(glm.nb(AD2~Phd+offset(log(S_AD)), data = data))$coefficients[2,4]

e_ad2_phd_nb_beta0<-summary(glm.nb(AD2~Phd+offset(log(S_AD)), data = data))$coefficients[1,1]

e_ad2_phd_nb_beta0_se<-summary(glm.nb(AD2~Phd+offset(log(S_AD)), data = data))$coefficients[1,2]

e_ad2_phd_nb_beta1<-summary(glm.nb(AD2~Phd+offset(log(S_AD)), data = data))$coefficients[2,1]

e_ad2_phd_nb_beta1_se<-summary(glm.nb(AD2~Phd+offset(log(S_AD)), data = data))$coefficients[2,2]}

#zero inflated negative binomial AD2~Phd

cc <- try(zeroinfl(AD2~Phd,data=data,offset=log(S_AD),dist = "negbin"), silent=T)

xx <- try(zeroinfl(AD2~1, data=data,offset=log(S_AD),dist = "negbin"),silent=T)

if(is(cc,"try-error")) {ad2_phd_nb_zi<-NA

e_ad2_phd_nb_zi_beta0c<-NA

e_ad2_phd_nb_zi_beta0c_se<-NA

e_ad2_phd_nb_zi_beta1c<-NA

e_ad2_phd_nb_zi_beta1c_se<-NA

e_ad2_phd_nb_zi_logthetac<-NA

e_ad2_phd_nb_zi_logthetac_se<-NA

e_ad2_phd_nb_zi_beta0z<-NA

e_ad2_phd_nb_zi_beta0z_se<-NA

e_ad2_phd_nb_zi_beta1z<-NA

e_ad2_phd_nb_zi_beta1z_se<-NA}

if(is(xx,"try-error")) {ad2_phd_nb_zi<-NA

e_ad2_phd_nb_zi_beta0c<-NA

e_ad2_phd_nb_zi_beta0c_se<-NA

e_ad2_phd_nb_zi_beta1c<-NA

e_ad2_phd_nb_zi_beta1c_se<-NA

e_ad2_phd_nb_zi_logthetac<-NA

e_ad2_phd_nb_zi_logthetac_se<-NA

e_ad2_phd_nb_zi_beta0z<-NA

e_ad2_phd_nb_zi_beta0z_se<-NA

e_ad2_phd_nb_zi_beta1z<-NA

e_ad2_phd_nb_zi_beta1z_se<-NA}

if(is(cc,"try-error")==F & is(xx,"try-error")==F) { ad2_phd_nb_zi<-1-pchisq(-2*(zeroinfl(AD2~1,data=data,offset=log(S_AD),dist = "negbin")$loglik-zeroinfl(AD2~Phd,data=data,offset=log(S_AD),dist = "negbin")$loglik),2)

e_ad2_phd_nb_zi_beta0c<-summary(zeroinfl(AD2~Phd,data=data,offset=log(S_AD),dist = "negbin"))$coefficients$count[1,1]

e_ad2_phd_nb_zi_beta0c_se<-summary(zeroinfl(AD2~Phd,data=data,offset=log(S_AD),dist = "negbin"))$coefficients$count[1,2]

e_ad2_phd_nb_zi_beta1c<-summary(zeroinfl(AD2~Phd,data=data,offset=log(S_AD),dist = "negbin"))$coefficients$count[2,1]

e_ad2_phd_nb_zi_beta1c_se<-summary(zeroinfl(AD2~Phd,data=data,offset=log(S_AD),dist = "negbin"))$coefficients$count[2,2]

e_ad2_phd_nb_zi_logthetac<-summary(zeroinfl(AD2~Phd,data=data,offset=log(S_AD),dist = "negbin"))$coefficients$count[3,1]

e_ad2_phd_nb_zi_logthetac_se<-summary(zeroinfl(AD2~Phd,data=data,offset=log(S_AD),dist = "negbin"))$coefficients$count[3,2]

e_ad2_phd_nb_zi_beta0z<-summary(zeroinfl(AD2~Phd,data=data,offset=log(S_AD),dist = "negbin"))$coefficients$zero[1,1]

e_ad2_phd_nb_zi_beta0z_se<-summary(zeroinfl(AD2~Phd,data=data,offset=log(S_AD),dist = "negbin"))$coefficients$zero[1,2]

e_ad2_phd_nb_zi_beta1z<-summary(zeroinfl(AD2~Phd,data=data,offset=log(S_AD),dist = "negbin"))$coefficients$zero[2,1]

e_ad2_phd_nb_zi_beta1z_se<-summary(zeroinfl(AD2~Phd,data=data,offset=log(S_AD),dist = "negbin"))$coefficients$zero[2,2]}

#hurdle negative binomial AD2~Phd

cc <- try(hurdle(AD2~Phd, data=data,offset=log(S_AD),dist = "negbin"), silent=T)

xx <- try(hurdle(AD2~1, data=data,offset=log(S_AD),dist = "negbin"),silent=T)

if(is(cc,"try-error")) {ad2_phd_nb_hur<-NA

e_ad2_phd_nb_hur_beta0c<-NA

e_ad2_phd_nb_hur_beta0c_se<-NA

e_ad2_phd_nb_hur_beta1c<-NA

e_ad2_phd_nb_hur_beta1c_se<-NA

e_ad2_phd_nb_hur_logthetac<-NA

e_ad2_phd_nb_hur_logthetac_se<-NA

e_ad2_phd_nb_hur_beta0z<-NA

e_ad2_phd_nb_hur_beta0z_se<-NA

e_ad2_phd_nb_hur_beta1z<-NA

e_ad2_phd_nb_hur_beta1z_se<-NA }

if(is(xx,"try-error")) {ad2_phd_nb_hur<-NA

e_ad2_phd_nb_hur_beta0c<-NA

e_ad2_phd_nb_hur_beta0c_se<-NA

e_ad2_phd_nb_hur_beta1c<-NA

e_ad2_phd_nb_hur_beta1c_se<-NA

e_ad2_phd_nb_hur_logthetac<-NA

e_ad2_phd_nb_hur_logthetac_se<-NA

e_ad2_phd_nb_hur_beta0z<-NA

e_ad2_phd_nb_hur_beta0z_se<-NA

e_ad2_phd_nb_hur_beta1z<-NA

e_ad2_phd_nb_hur_beta1z_se<-NA }

if(is(cc,"try-error")==F & is(xx,"try-error")==F) { ad2_phd_nb_hur<-1-pchisq(-2*(hurdle(AD2~1, data=data,offset=log(S_AD),dist = "negbin")$loglik-hurdle(AD2~Phd, data=data,offset=log(S_AD),dist = "negbin")$loglik),2)

e_ad2_phd_nb_hur_beta0c<-summary(hurdle(AD2~Phd, data=data,offset=log(S_AD),dist = "negbin"))$coefficients$count[1,1]

e_ad2_phd_nb_hur_beta0c_se<-summary(hurdle(AD2~Phd, data=data,offset=log(S_AD),dist = "negbin"))$coefficients$count[1,2]

e_ad2_phd_nb_hur_beta1c<-summary(hurdle(AD2~Phd, data=data,offset=log(S_AD),dist = "negbin"))$coefficients$count[2,1]

e_ad2_phd_nb_hur_beta1c_se<-summary(hurdle(AD2~Phd, data=data,offset=log(S_AD),dist = "negbin"))$coefficients$count[2,2]

e_ad2_phd_nb_hur_logthetac<-summary(hurdle(AD2~Phd, data=data,offset=log(S_AD),dist = "negbin"))$coefficients$count[3,1]

e_ad2_phd_nb_hur_logthetac_se<-summary(hurdle(AD2~Phd, data=data,offset=log(S_AD),dist = "negbin"))$coefficients$count[3,2]

e_ad2_phd_nb_hur_beta0z<-summary(hurdle(AD2~Phd, data=data,offset=log(S_AD),dist = "negbin"))$coefficients$zero[1,1]

e_ad2_phd_nb_hur_beta0z_se<-summary(hurdle(AD2~Phd, data=data,offset=log(S_AD),dist = "negbin"))$coefficients$zero[1,2]

e_ad2_phd_nb_hur_beta1z<-summary(hurdle(AD2~Phd, data=data,offset=log(S_AD),dist = "negbin"))$coefficients$zero[2,1]

e_ad2_phd_nb_hur_beta1z_se<-summary(hurdle(AD2~Phd, data=data,offset=log(S_AD),dist = "negbin"))$coefficients$zero[2,2] }

#######################################################################################################################

#################################### ratio~Phd ###############################################################

#######################################################################################################################

#standard linear model Phd~AD2

cc <- try(lm(ratio~Phd, data = data), silent=T)

xx<- try(summary(lm(ratio~Phd, data = data)), silent=T)

if(is(cc,"try-error")) { ratio_phd_lm<-NA

e_ratio_phd_lm_beta0<-NA

e_ratio_phd_lm_beta0_se<-NA

e_ratio_phd_lm_beta1<-NA

e_ratio_phd_lm_beta1_se<-NA}

if(is(xx,"try-error")) { ratio_phd_lm<-NA

e_ratio_phd_lm_beta0<-NA

e_ratio_phd_lm_beta0_se<-NA

e_ratio_phd_lm_beta1<-NA

e_ratio_phd_lm_beta1_se<-NA}

if(is(cc,"try-error")==F & is(xx,"try-error")==F) { ratio_phd_lm<-summary(lm(ratio~Phd, data = data))$coefficients[2,4]

e_ratio_phd_lm_beta0<-summary(lm(ratio~Phd, data = data))$coefficients[1,1]

e_ratio_phd_lm_beta0_se<-summary(lm(ratio~Phd, data = data))$coefficients[1,2]

e_ratio_phd_lm_beta1<-summary(lm(ratio~Phd, data = data))$coefficients[2,1]

e_ratio_phd_lm_beta1_se<-summary(lm(ratio~Phd, data = data))$coefficients[2,2]}

#######################################################################################################################

#################################### Phd~ratio ###############################################################

#######################################################################################################################

#logistic regression standard Phd~ratio

cc <- try(glm(Phd~ratio, family=binomial(link=logit),data=data), silent=T)

xx<- try(summary(glm(Phd~ratio, family=binomial(link=logit),data=data), silent=T))

if(is(cc,"try-error")) { phd_ratio_logit<-NA

e_phd_ratio_logit_beta0<-NA

e_phd_ratio_logit_beta0_se<-NA

e_phd_ratio_logit_beta1<-NA

e_phd_ratio_logit_beta1_se<-NA}

if(is(xx,"try-error")) { phd_ratio_logit<-NA

e_phd_ratio_logit_beta0<-NA

e_phd_ratio_logit_beta0_se<-NA

e_phd_ratio_logit_beta1<-NA

e_phd_ratio_logit_beta1_se<-NA}

if(is(cc,"try-error")==F & is(xx,"try-error")==F) { phd_ratio_logit<-summary(glm(Phd~ratio, family=binomial(link=logit),data=data))$coefficients[2,4]

e_phd_ratio_logit_beta0<-summary(glm(Phd~ratio, family=binomial(link=logit),data=data))$coefficients[1,1]

e_phd_ratio_logit_beta0_se<-summary(glm(Phd~ratio, family=binomial(link=logit),data=data))$coefficients[1,2]

e_phd_ratio_logit_beta1<-summary(glm(Phd~ratio, family=binomial(link=logit),data=data))$coefficients[2,1]

e_phd_ratio_logit_beta1_se<-summary(glm(Phd~ratio, family=binomial(link=logit),data=data))$coefficients[2,2]}

#######################################################################################################################

#################################### GT_estim~Phd ############################################################

#######################################################################################################################

#ordinal logistic standard model GT_estim~Phd

cc <- try(polr(GT_estim~Phd, data = data, Hess = TRUE), silent=T)

if(is(cc,"try-error")) {gt_phd_ordinal_logit<-NA

e_gt_phd_ordinal_logit_coef<-NA

e_gt_phd_ordinal_logit_coef_se<-NA

e_gt_phd_ordinal_logit_int1<-NA

e_gt_phd_ordinal_logit_int1_se<-NA

e_gt_phd_ordinal_logit_int2<-NA

e_gt_phd_ordinal_logit_int2_se<-NA}

if(is(cc,"try-error")==F) {gt_phd_ordinal_logit<-pnorm(abs(summary( polr(GT_estim~Phd, data = data, Hess = TRUE) )$coefficients[1,3]),lower.tail=F)*2

e_gt_phd_ordinal_logit_coef<-summary( polr(GT_estim~Phd, data = data, Hess = TRUE) )$coefficients[1,1]

e_gt_phd_ordinal_logit_coef_se<-summary( polr(GT_estim~Phd, data = data, Hess = TRUE) )$coefficients[1,2]

e_gt_phd_ordinal_logit_int1<-summary( polr(GT_estim~Phd, data = data, Hess = TRUE) )$coefficients[2,1]

e_gt_phd_ordinal_logit_int1_se<-summary( polr(GT_estim~Phd, data = data, Hess = TRUE) )$coefficients[2,2]

e_gt_phd_ordinal_logit_int2<-summary( polr(GT_estim~Phd, data = data, Hess = TRUE) )$coefficients[3,1]

e_gt_phd_ordinal_logit_int2_se<-summary( polr(GT_estim~Phd, data = data, Hess = TRUE) )$coefficients[3,2]}

#logistic regression standard GT_estim_g~Phd

cc <- try(glm(GT_estim_g~Phd, family=binomial(link=logit),data=data), silent=T)

xx<- try(summary(glm(GT_estim_g~Phd, family=binomial(link=logit),data=data), silent=T))

if(is(cc,"try-error")) { gt_phd_logit<-NA

e_gt_phd_logit_beta0<-NA

e_gt_phd_logit_beta0_se<-NA

e_gt_phd_logit_beta1<-NA

e_gt_phd_logit_beta1_se<-NA}

if(is(xx,"try-error")) { gt_phd_logit<-NA

e_gt_phd_logit_beta0<-NA

e_gt_phd_logit_beta0_se<-NA

e_gt_phd_logit_beta1<-NA

e_gt_phd_logit_beta1_se<-NA}

if(is(cc,"try-error")==F & is(xx,"try-error")==F) { gt_phd_logit<-summary(glm(GT_estim_g~Phd, family=binomial(link=logit),data=data))$coefficients[2,4]

e_gt_phd_logit_beta0<-summary(glm(GT_estim_g~Phd, family=binomial(link=logit),data=data))$coefficients[1,1]

e_gt_phd_logit_beta0_se<-summary(glm(GT_estim_g~Phd, family=binomial(link=logit),data=data))$coefficients[1,2]

e_gt_phd_logit_beta1<-summary(glm(GT_estim_g~Phd, family=binomial(link=logit),data=data))$coefficients[2,1]

e_gt_phd_logit_beta1_se<-summary(glm(GT_estim_g~Phd, family=binomial(link=logit),data=data))$coefficients[2,2]}

#######################################################################################################################

#################################### Phd~GT_estim ###########################################################

#######################################################################################################################

#logistic regression standard Phd~GT_estim

cc <- try(glm(Phd~GT_estim, family=binomial(link=logit),data=data), silent=T)

xx<- try(summary(glm(Phd~GT_estim, family=binomial(link=logit),data=data), silent=T))

if(is(cc,"try-error")) { phd_gt_logit<-NA

e_phd_gt_logit_beta0<-NA

e_phd_gt_logit_beta0_se<-NA

e_phd_gt_logit_beta1<-NA

e_phd_gt_logit_beta1_se<-NA}

if(is(xx,"try-error")) { phd_gt_logit<-NA

e_phd_gt_logit_beta0<-NA

e_phd_gt_logit_beta0_se<-NA

e_phd_gt_logit_beta1<-NA

e_phd_gt_logit_beta1_se<-NA}

if(is(cc,"try-error")==F & is(xx,"try-error")==F) { phd_gt_logit<-summary(glm(Phd~GT_estim, family=binomial(link=logit),data=data))$coefficients[2,4]

e_phd_gt_logit_beta0<-summary(glm(Phd~GT_estim, family=binomial(link=logit),data=data))$coefficients[1,1]

e_phd_gt_logit_beta0_se<-summary(glm(Phd~GT_estim, family=binomial(link=logit),data=data))$coefficients[1,2]

e_phd_gt_logit_beta1<-summary(glm(Phd~GT_estim, family=binomial(link=logit),data=data))$coefficients[2,1]

e_phd_gt_logit_beta1_se<-summary(glm(Phd~GT_estim, family=binomial(link=logit),data=data))$coefficients[2,2]}

#######################################################################################################################

#################################### GT_real~Phd ############################################################

#######################################################################################################################

#ordinal logistic standard model GT_real~Phd

cc <- try(polr(GT_real~Phd, data = data, Hess = TRUE), silent=T)

if(is(cc,"try-error")) {gtr_phd_ordinal_logit<-NA

e_gtr_phd_ordinal_logit_coef<-NA

e_gtr_phd_ordinal_logit_coef_se<-NA

e_gtr_phd_ordinal_logit_int1<-NA

e_gtr_phd_ordinal_logit_int1_se<-NA

e_gtr_phd_ordinal_logit_int2<-NA

e_gtr_phd_ordinal_logit_int2_se<-NA}

if(is(cc,"try-error")==F) {gtr_phd_ordinal_logit<-pnorm(abs(summary( polr(GT_real~Phd, data = data, Hess = TRUE) )$coefficients[1,3]),lower.tail=F)*2

e_gtr_phd_ordinal_logit_coef<-summary( polr(GT_real~Phd, data = data, Hess = TRUE) )$coefficients[1,1]

e_gtr_phd_ordinal_logit_coef_se<-summary( polr(GT_real~Phd, data = data, Hess = TRUE) )$coefficients[1,2]

e_gtr_phd_ordinal_logit_int1<-summary( polr(GT_real~Phd, data = data, Hess = TRUE) )$coefficients[2,1]

e_gtr_phd_ordinal_logit_int1_se<-summary( polr(GT_real~Phd, data = data, Hess = TRUE) )$coefficients[2,2]

e_gtr_phd_ordinal_logit_int2<-summary( polr(GT_real~Phd, data = data, Hess = TRUE) )$coefficients[3,1]

e_gtr_phd_ordinal_logit_int2_se<-summary( polr(GT_real~Phd, data = data, Hess = TRUE) )$coefficients[3,2]}

#logistic regression standard GT_real~Phd

cc <- try(glm(GT_real~Phd, family=binomial(link=logit),data=data), silent=T)

xx<- try(summary(glm(GT_real~Phd, family=binomial(link=logit),data=data), silent=T))

if(is(cc,"try-error")) { gtr_phd_logit<-NA

e_gtr_phd_logit_beta0<-NA

e_gtr_phd_logit_beta0_se<-NA

e_gtr_phd_logit_beta1<-NA

e_gtr_phd_logit_beta1_se<-NA}

if(is(xx,"try-error")) { gtr_phd_logit<-NA

e_gtr_phd_logit_beta0<-NA

e_gtr_phd_logit_beta0_se<-NA

e_gtr_phd_logit_beta1<-NA

e_gtr_phd_logit_beta1_se<-NA}

if(is(cc,"try-error")==F & is(xx,"try-error")==F) { gtr_phd_logit<-summary(glm(GT_real~Phd, family=binomial(link=logit),data=data))$coefficients[2,4]

e_gtr_phd_logit_beta0<-summary(glm(GT_real~Phd, family=binomial(link=logit),data=data))$coefficients[1,1]

e_gtr_phd_logit_beta0_se<-summary(glm(GT_real~Phd, family=binomial(link=logit),data=data))$coefficients[1,2]

e_gtr_phd_logit_beta1<-summary(glm(GT_real~Phd, family=binomial(link=logit),data=data))$coefficients[2,1]

e_gtr_phd_logit_beta1_se<-summary(glm(GT_real~Phd, family=binomial(link=logit),data=data))$coefficients[2,2]}

#######################################################################################################################

#################################### Phd~GT_real ###########################################################

#######################################################################################################################

#logistic regression standard Phd~GT_real

cc <- try(glm(Phd~GT_real, family=binomial(link=logit),data=data), silent=T)

xx<- try(summary(glm(Phd~GT_real, family=binomial(link=logit),data=data), silent=T))

if(is(cc,"try-error")) { phd_gtr_logit<-NA

e_phd_gtr_logit_beta0<-NA

e_phd_gtr_logit_beta0_se<-NA

e_phd_gtr_logit_beta1<-NA

e_phd_gtr_logit_beta1_se<-NA}

if(is(xx,"try-error")) { phd_gtr_logit<-NA

e_phd_gtr_logit_beta0<-NA

e_phd_gtr_logit_beta0_se<-NA

e_phd_gtr_logit_beta1<-NA

e_phd_gtr_logit_beta1_se<-NA}

if(is(cc,"try-error")==F & is(xx,"try-error")==F) { phd_gtr_logit<-summary(glm(Phd~GT_real, family=binomial(link=logit),data=data))$coefficients[2,4]

e_phd_gtr_logit_beta0<-summary(glm(Phd~GT_real, family=binomial(link=logit),data=data))$coefficients[1,1]

e_phd_gtr_logit_beta0_se<-summary(glm(Phd~GT_real, family=binomial(link=logit),data=data))$coefficients[1,2]

e_phd_gtr_logit_beta1<-summary(glm(Phd~GT_real, family=binomial(link=logit),data=data))$coefficients[2,1]

e_phd_gtr_logit_beta1_se<-summary(glm(Phd~GT_real, family=binomial(link=logit),data=data))$coefficients[2,2]}

test_df<-data.frame(rs=gt_pos$rs,

alleles=gt_pos$alleles,

chrom=gt_pos$chrom,

pos=gt_pos$pos,

ad2_ph_poisson,

ad2_ph_nb,

ad2_ph_nb_zi,

ad2_ph_nb_hur,

ratio_ph_lm,

ph_ratio_lm,

gt_ph_ordinal_logit,

gt_ph_logit,

ph_gt_lm,

gtr_ph_ordinal_logit,

gtr_ph_logit,

ph_gtr_lm,

ad2_phd_poisson,

ad2_phd_nb,

ad2_phd_nb_zi,

ad2_phd_nb_hur,

ratio_phd_lm,

phd_ratio_logit,

gt_phd_ordinal_logit,

gt_phd_logit,

phd_gt_logit,

gtr_phd_ordinal_logit,

gtr_phd_logit,

phd_gtr_logit)

results_pvalue<-rbind(results_pvalue,test_df)

estim_df<-data.frame(rs=gt_pos$rs,

alleles=gt_pos$alleles,

chrom=gt_pos$chrom,

pos=gt_pos$pos,

e_ad2_phd_poisson_beta0 ,

e_ad2_phd_poisson_beta0_se ,

e_ad2_phd_poisson_beta1 ,

e_ad2_phd_poisson_beta1_se ,

e_ad2_phd_nb_beta0 ,

e_ad2_phd_nb_beta0_se ,

e_ad2_phd_nb_beta1 ,

e_ad2_phd_nb_beta1_se ,

e_ad2_phd_nb_zi_beta0c ,

e_ad2_phd_nb_zi_beta0c_se ,

e_ad2_phd_nb_zi_beta1c ,

e_ad2_phd_nb_zi_beta1c_se ,

e_ad2_phd_nb_zi_logthetac ,

e_ad2_phd_nb_zi_logthetac_se ,

e_ad2_phd_nb_zi_beta0z ,

e_ad2_phd_nb_zi_beta0z_se ,

e_ad2_phd_nb_zi_beta1z ,

e_ad2_phd_nb_zi_beta1z_se ,

e_ad2_phd_nb_hur_beta0c ,

e_ad2_phd_nb_hur_beta0c_se ,

e_ad2_phd_nb_hur_beta1c ,

e_ad2_phd_nb_hur_beta1c_se ,

e_ad2_phd_nb_hur_logthetac ,

e_ad2_phd_nb_hur_logthetac_se ,

e_ad2_phd_nb_hur_beta0z ,

e_ad2_phd_nb_hur_beta0z_se ,

e_ad2_phd_nb_hur_beta1z ,

e_ad2_phd_nb_hur_beta1z_se ,

e_ratio_phd_lm_beta0 ,

e_ratio_phd_lm_beta0_se ,

e_ratio_phd_lm_beta1 ,

e_ratio_phd_lm_beta1_se ,

e_phd_ratio_logit_beta0 ,

e_phd_ratio_logit_beta0_se ,

e_phd_ratio_logit_beta1 ,

e_phd_ratio_logit_beta1_se ,

e_gt_phd_ordinal_logit_coef ,

e_gt_phd_ordinal_logit_coef_se ,

e_gt_phd_ordinal_logit_int1 ,

e_gt_phd_ordinal_logit_int1_se ,

e_gt_phd_ordinal_logit_int2 ,

e_gt_phd_ordinal_logit_int2_se ,

e_gt_phd_logit_beta0 ,

e_gt_phd_logit_beta0_se ,

e_gt_phd_logit_beta1 ,

e_gt_phd_logit_beta1_se ,

e_phd_gt_logit_beta0 ,

e_phd_gt_logit_beta0_se ,

e_phd_gt_logit_beta1 ,

e_phd_gt_logit_beta1_se ,

e_gtr_phd_ordinal_logit_coef ,

e_gtr_phd_ordinal_logit_coef_se ,

e_gtr_phd_ordinal_logit_int1 ,

e_gtr_phd_ordinal_logit_int1_se ,

e_gtr_phd_ordinal_logit_int2 ,

e_gtr_phd_ordinal_logit_int2_se ,

e_gtr_phd_logit_beta0 ,

e_gtr_phd_logit_beta0_se ,

e_gtr_phd_logit_beta1 ,

e_gtr_phd_logit_beta1_se ,

e_phd_gtr_logit_beta0 ,

e_phd_gtr_logit_beta0_se ,

e_phd_gtr_logit_beta1 ,

e_phd_gtr_logit_beta1_se )

results_estim<-rbind(results_estim,estim_df)

test_variant<-data.frame(rs=gt_pos$rs,

alleles=gt_pos$alleles,

chrom=gt_pos$chrom,

pos=gt_pos$pos,medianGQ,

medianSAD)

test_table<-data.frame(rs=gt_pos$rs,

alleles=gt_pos$alleles,

chrom=gt_pos$chrom,

pos=gt_pos$pos,r0e0,

r0e1,

r0e2,

r1e0,

r1e1,

r1e2,

r2e0,

r2e1,

r2e2)

results_variant<-rbind(results_variant,test_variant)

results_table<-rbind(results_table,test_table)

write.table(results_pvalue,"results_pvalue_a1.txt")

write.table(results_estim,"results_estim_a1.txt")

write.table(results_variant,"results_variant_a1.txt")

write.table(results_table,"results_table_a1.txt")

b<-Sys.time()

print(b-a)

} #fin position p

#Tables and plots

Sys.setenv(LANG = "en")

freq<- read.csv("plink.frq", sep="")

data_a<- read.csv("~/AA_MY_PHD/AAanalysis/chr20/all_variants/00_PREPROCESING/results_pvalue_a.txt", sep="")

data_n<- read.csv("~/AA_MY_PHD/AAanalysis/chr20/all_variants/00_PREPROCESING/results_pvalue_na.txt", sep="")

#var_a<- read.csv("results_variant_a.txt", sep="")

#var_na<- read.csv("results_variant_na.txt", sep="")

#consider pv=0 as NA

for(j in 5:28){

for(i in 1:length(data_a[,1])){

if( is.na(data_a[i,j])){data_a[i,j]<-NA}

else{

if(data_a[i,j]==0){data_a[i,j]<-NA}

}

}

print(j)

}

for(j in 5:28){

for(i in 1:length(data_n[,1])){

if( is.na(data_n[i,j])){data_n[i,j]<-NA}

else{

if(data_n[i,j]==0){data_n[i,j]<-NA}

}

}

print(j)

}

#remove positions out of rank

rownames(data_n)<-data_n$rs

rownames(data_a)<-data_a$rs

#overlapped

library(VennDiagram)

l1<-list(data_a$rs,data_n$rs)

Intersect <- function (x) {

# Multiple set version of intersect

# x is a list

if (length(x) == 1) {

unlist(x)

} else if (length(x) == 2) {

intersect(x[[1]], x[[2]])

} else if (length(x) > 2){

intersect(x[[1]], Intersect(x[-1]))

}

}

names_rs<-Intersect(l1)

data_a<-data_a[names_rs,]

data_n<-data_n[names_rs,]

write.table(data_a,"data_a.txt")

write.table(data_n,"data_n.txt")

create.tables<-function(data_a,data_n){

#na

na.a<-apply(data_a[,-c(1,2,3,4)], 2,function(x) {sum(is.na(x))})

#tot

tot.n.na.a<-length(data_a[,1])-na.a

#percemntaghe

per.n.na.a<-tot.n.na.a*100/length(data_a[,1])

#sig

sig.a<-apply(data_a[,-c(1,2,3,4)], 2,function(x) {sum(x<=0.05,na.rm=TRUE)})

#sig perc

per.a<-sig.a/tot.n.na.a

#t1errorrate no assoc

#na

na.n<-apply(data_n[,-c(1,2,3,4)], 2,function(x) {sum(is.na(x))})

#tot

tot.n.na.n<-length(data_n[,1])-na.n

#percemntaghe

per.n.na.n<-tot.n.na.n*100/length(data_n[,1])

#sig

sig.n<-apply(data_n[,-c(1,2,3,4)], 2,function(x) {sum(x<=0.05,na.rm=TRUE)})

#sig perc

per.n<-sig.n/tot.n.na.n

results<-data.frame(pv.a= rep(length(data_a[,1]),24),tot.n.na.a=tot.n.na.a,per.n.na.a=per.n.na.a, assoc=per.a,pv.n= rep(length(data_n[,1]),24),tot.n.na.n=tot.n.na.n,per.n.na.n=per.n.na.n, no_assoc=per.n)

return(results)

}

a<-create.tables(data_a,data_n)

View(a)

write.table(a,"table1.txt")

#add maf to data_a

rownames(freq)<-freq$SNP

rownames(data_a)<-data_a$rs

maf<-freq[rownames(data_a),]

data_a<-data.frame(data_a,maf=0)

for(i in data_a$rs){

data_a[i,29]<-maf[i,5]

print(i)

}

#add maf to data_na

rownames(freq)<-freq$SNP

rownames(data_n)<-data_n$rs

maf<-freq[rownames(data_n),]

data_n<-data.frame(data_n,maf=0)

for(i in data_n$rs){

data_n[i,29]<-maf[i,5]

print(i)

}

#stratify by maf

maf1_a<-subset(data_a,data_a$maf<=0.05)[-29]

maf2_a<-subset(data_a,data_a$maf>0.05 & data_a$maf<=0.1)[-29]

maf3_a<-subset(data_a,data_a$maf>0.1 & data_a$maf<=0.2)[-29]

maf4_a<-subset(data_a,data_a$maf>0.2)[-29]

maf1_n<-subset(data_n,data_n$maf<=0.05)[-29]

maf2_n<-subset(data_n,data_n$maf>0.05 & data_n$maf<=0.1)[-29]

maf3_n<-subset(data_n,data_n$maf>0.1 & data_n$maf<=0.2)[-29]

maf4_n<-subset(data_n,data_n$maf>0.2)[-29]

maf1<-create.tables(maf1_a,maf1_n)

maf2<-create.tables(maf2_a,maf2_n)

maf3<-create.tables(maf3_a,maf3_n)

maf4<-create.tables(maf4_a,maf4_n)

maf<-rbind( maf1[1,],maf2[1,],maf3[1,],maf4[1,],maf1[2,],maf2[2,],maf3[2,],maf4[2,],maf1[3,], maf2[3,], maf3[3,], maf4[3,], maf1[4,],maf2[4,], maf3[4,], maf4[4,], maf1[5,], maf2[5,], maf3[5,],maf4[5,],maf1[6,], maf2[6,], maf3[6,], maf4[6,], maf1[7,], maf2[7,], maf3[7,], maf4[7,], maf1[8,], maf2[8,], maf3[8,], maf4[8,], maf1[9,], maf2[9,], maf3[9,], maf4[9,], maf1[10,],maf2[10,],maf3[10,],maf4[10,],maf1[11,], maf2[11,], maf3[11,], maf4[11,], maf1[12,], maf2[12,], maf3[12,], maf4[12,], maf1[13,], maf2[13,], maf3[13,], maf4[13,], maf1[14,], maf2[14,], maf3[14,], maf4[14,], maf1[15,], maf2[15,], maf3[15,], maf4[15,], maf1[16,], maf2[16,], maf3[16,], maf4[16,], maf1[17,], maf2[17,], maf3[17,], maf4[17,], maf1[18,], maf2[18,], maf3[18,], maf4[18,], maf1[19,], maf2[19,], maf3[19,], maf4[19,], maf1[20,], maf2[20,], maf3[20,], maf4[20,], maf1[21,], maf2[21,], maf3[21,], maf4[21,], maf1[22,], maf2[22,], maf3[22,], maf4[22,], maf1[23,], maf2[23,], maf3[23,], maf4[23,], maf1[24,], maf2[24,], maf3[24,], maf4[24,], maf1[25,], maf2[25,], maf3[25,], maf4[25,], maf1[26,], maf2[26,], maf3[26,], maf4[26,], maf1[27,], maf2[27,], maf3[27,], maf4[27,], maf1[28,], maf2[28,], maf3[28,], maf4[28,] )

View(maf)

write.table(maf,"table2_maf_4g.txt")

#stratify by alleles

data_a<-data_a[,-29]

data_n<-data_n[,-29]

AC_a<-subset(data_a,data_a$alleles=="A/C")

AG_a<-subset(data_a,data_a$alleles=="A/G")

AT_a<-subset(data_a,data_a$alleles=="A/T")

CG_a<-subset(data_a,data_a$alleles=="C/G")

CT_a<-subset(data_a,data_a$alleles=="C/T")

GT_a<-subset(data_a,data_a$alleles=="G/T")

AC_n<-subset(data_n,data_n$alleles=="A/C")

AG_n<-subset(data_n,data_n$alleles=="A/G")

AT_n<-subset(data_n,data_n$alleles=="A/T")

CG_n<-subset(data_n,data_n$alleles=="C/G")

CT_n<-subset(data_n,data_n$alleles=="C/T")

GT_n<-subset(data_n,data_n$alleles=="G/T")

AC<-create.tables(AC_a,AC_n)

AG<-create.tables(AG_a,AG_n)

AT<-create.tables(AT_a,AT_n)

CG<-create.tables(CG_a,CG_n)

CT<-create.tables(CT_a,CT_n)

GT<-create.tables(GT_a,GT_n)

alleles<-rbind(AC[1,], AG[1,], AT[1,], CG[1,], CT[1,], GT[1,], AC[2,], AG[2,], AT[2,], CG[2,], CT[2,], GT[2,], AC[3,], AG[3,], AT[3,], CG[3,], CT[3,], GT[3,], AC[4,], AG[4,], AT[4,], CG[4,], CT[4,], GT[4,], AC[5,], AG[5,], AT[5,], CG[5,], CT[5,], GT[5,], AC[6,], AG[6,], AT[6,], CG[6,], CT[6,], GT[6,], AC[7,], AG[7,], AT[7,], CG[7,], CT[7,], GT[7,], AC[8,], AG[8,], AT[8,], CG[8,], CT[8,], GT[8,], AC[9,], AG[9,], AT[9,], CG[9,], CT[9,], GT[9,], AC[10,], AG[10,], AT[10,], CG[10,], CT[10,], GT[10,], AC[11,], AG[11,], AT[11,], CG[11,], CT[11,], GT[11,], AC[12,], AG[12,], AT[12,], CG[12,], CT[12,], GT[12,], AC[13,], AG[13,], AT[13,], CG[13,], CT[13,], GT[13,], AC[14,], AG[14,], AT[14,], CG[14,], CT[14,], GT[14,], AC[15,], AG[15,], AT[15,], CG[15,], CT[15,], GT[15,], AC[16,], AG[16,], AT[16,], CG[16,], CT[16,], GT[16,], AC[17,], AG[17,], AT[17,], CG[17,], CT[17,], GT[17,], AC[18,], AG[18,], AT[18,], CG[18,], CT[18,], GT[18,], AC[19,], AG[19,], AT[19,], CG[19,], CT[19,], GT[19,], AC[20,], AG[20,], AT[20,], CG[20,], CT[20,], GT[20,], AC[21,], AG[21,], AT[21,], CG[21,], CT[21,], GT[21,], AC[22,], AG[22,], AT[22,], CG[22,], CT[22,], GT[22,], AC[23,], AG[23,], AT[23,], CG[23,], CT[23,], GT[23,], AC[24,], AG[24,], AT[24,], CG[24,], CT[24,], GT[24,], AC[25,], AG[25,], AT[25,], CG[25,], CT[25,], GT[25,], AC[26,], AG[26,], AT[26,], CG[26,], CT[26,], GT[26,], AC[27,], AG[27,], AT[27,], CG[27,], CT[27,], GT[27,], AC[28,], AG[28,], AT[28,])

View(alleles)

write.table(alleles,"table3.txt")

###############################################################################################################################################

############ add coverage

info_a<- read.csv("~/AA_MY_PHD/AAanalysis/chr20/all_variants/00_PREPROCESING/results_variant_a.txt", sep="")

data_a<- read.csv("~/AA_MY_PHD/AAanalysis/chr20/all_variants/00_PREPROCESING/results_pvalue_a.txt", sep="")

data_n<- read.csv("~/AA_MY_PHD/AAanalysis/chr20/all_variants/00_PREPROCESING/results_pvalue_na.txt", sep="")

#stratify by coverage

data_a<-data.frame(data_a,coverage=info_a$medianSAD)

data_n<-data.frame(data_n,coverage=info_a$medianSAD)

coverage1_a<-subset(data_a,data_a$coverage<=summary(data_a$coverage)[2])[,-29]

coverage2_a<-subset(data_a,data_a$coverage<=summary(data_a$coverage)[3] & data_a$coverage>summary(data_a$coverage)[2])[,-29]

coverage3_a<-subset(data_a,data_a$coverage<=summary(data_a$coverage)[5] & data_a$coverage>summary(data_a$coverage)[3])[,-29]

coverage4_a<-subset(data_a,data_a$coverage>summary(data_a$coverage)[5])[,-29]

coverage1_n<-subset(data_n,data_n$coverage<=summary(data_n$coverage)[2])[,-29]

coverage2_n<-subset(data_n,data_n$coverage<=summary(data_n$coverage)[3] & data_n$coverage>summary(data_n$coverage)[2])[,-29]

coverage3_n<-subset(data_n,data_n$coverage<=summary(data_n$coverage)[5] & data_n$coverage>summary(data_n$coverage)[3])[,-29]

coverage4_n<-subset(data_n,data_n$coverage>summary(data_n$coverage)[5])[,-29]

coverage1<-create.tables(coverage1_a,coverage1_n)

coverage2<-create.tables(coverage2_a,coverage2_n)

coverage3<-create.tables(coverage3_a,coverage3_n)

coverage4<-create.tables(coverage4_a,coverage4_n)

coverage<-rbind( coverage1[1,],coverage2[1,],coverage3[1,],coverage4[1,],coverage1[2,],coverage2[2,],coverage3[2,],coverage4[2,],coverage1[3,], coverage2[3,], coverage3[3,], coverage4[3,], coverage1[4,],coverage2[4,], coverage3[4,], coverage4[4,], coverage1[5,], coverage2[5,], coverage3[5,],coverage4[5,],coverage1[6,], coverage2[6,], coverage3[6,], coverage4[6,], coverage1[7,], coverage2[7,], coverage3[7,], coverage4[7,], coverage1[8,], coverage2[8,], coverage3[8,], coverage4[8,], coverage1[9,], coverage2[9,], coverage3[9,], coverage4[9,], coverage1[10,],coverage2[10,],coverage3[10,],coverage4[10,],coverage1[11,], coverage2[11,], coverage3[11,], coverage4[11,], coverage1[12,], coverage2[12,], coverage3[12,], coverage4[12,], coverage1[13,], coverage2[13,], coverage3[13,], coverage4[13,], coverage1[14,], coverage2[14,], coverage3[14,], coverage4[14,], coverage1[15,], coverage2[15,], coverage3[15,], coverage4[15,], coverage1[16,], coverage2[16,], coverage3[16,], coverage4[16,], coverage1[17,], coverage2[17,], coverage3[17,], coverage4[17,], coverage1[18,], coverage2[18,], coverage3[18,], coverage4[18,], coverage1[19,], coverage2[19,], coverage3[19,], coverage4[19,], coverage1[20,], coverage2[20,], coverage3[20,], coverage4[20,], coverage1[21,], coverage2[21,], coverage3[21,], coverage4[21,], coverage1[22,], coverage2[22,], coverage3[22,], coverage4[22,], coverage1[23,], coverage2[23,], coverage3[23,], coverage4[23,], coverage1[24,], coverage2[24,], coverage3[24,], coverage4[24,], coverage1[25,], coverage2[25,], coverage3[25,], coverage4[25,], coverage1[26,], coverage2[26,], coverage3[26,], coverage4[26,], coverage1[27,], coverage2[27,], coverage3[27,], coverage4[27,], coverage1[28,], coverage2[28,], coverage3[28,], coverage4[28,] )

View(coverage)

write.table(coverage,"table2_coverage_4g.txt")

###############################################################################################################################################

############ add gq

info_a<- read.csv("~/AA_MY_PHD/AAanalysis/chr20/all_variants/00_PREPROCESING/results_variant_a.txt", sep="")

data_a<- read.csv("~/AA_MY_PHD/AAanalysis/chr20/all_variants/00_PREPROCESING/results_pvalue_a.txt", sep="")

data_n<- read.csv("~/AA_MY_PHD/AAanalysis/chr20/all_variants/00_PREPROCESING/results_pvalue_na.txt", sep="")

#

#stratify by gq

data_a<-data.frame(data_a,gq=info_a$medianGQ)

data_n<-data.frame(data_n,gq=info_a$medianGQ)

gq1_a<-subset(data_a,data_a$gq<=summary(data_a$gq)[2])[,-29]

gq2_a<-subset(data_a,data_a$gq<=summary(data_a$gq)[3] & data_a$gq>summary(data_a$gq)[2])[,-29]

gq3_a<-subset(data_a,data_a$gq<=summary(data_a$gq)[5] & data_a$gq>summary(data_a$gq)[3])[,-29]

gq4_a<-subset(data_a,data_a$gq>summary(data_a$gq)[5])[,-29]

gq1_n<-subset(data_n,data_n$gq<=summary(data_n$gq)[2])[,-29]

gq2_n<-subset(data_n,data_n$gq<=summary(data_n$gq)[3] & data_n$gq>summary(data_n$gq)[2])[,-29]

gq3_n<-subset(data_n,data_n$gq<=summary(data_n$gq)[5] & data_n$gq>summary(data_n$gq)[3])[,-29]

gq4_n<-subset(data_n,data_n$gq>summary(data_n$gq)[5])[,-29]

gq1<-create.tables(gq1_a,gq1_n)

gq2<-create.tables(gq2_a,gq2_n)

gq3<-create.tables(gq3_a,gq3_n)

gq4<-create.tables(gq4_a,gq4_n)

gq<-rbind( gq1[1,],gq2[1,],gq3[1,],gq4[1,],gq1[2,],gq2[2,],gq3[2,],gq4[2,],gq1[3,], gq2[3,], gq3[3,], gq4[3,], gq1[4,],gq2[4,], gq3[4,], gq4[4,], gq1[5,], gq2[5,], gq3[5,],gq4[5,],gq1[6,], gq2[6,], gq3[6,], gq4[6,], gq1[7,], gq2[7,], gq3[7,], gq4[7,], gq1[8,], gq2[8,], gq3[8,], gq4[8,], gq1[9,], gq2[9,], gq3[9,], gq4[9,], gq1[10,],gq2[10,],gq3[10,],gq4[10,],gq1[11,], gq2[11,], gq3[11,], gq4[11,], gq1[12,], gq2[12,], gq3[12,], gq4[12,], gq1[13,], gq2[13,], gq3[13,], gq4[13,], gq1[14,], gq2[14,], gq3[14,], gq4[14,], gq1[15,], gq2[15,], gq3[15,], gq4[15,], gq1[16,], gq2[16,], gq3[16,], gq4[16,], gq1[17,], gq2[17,], gq3[17,], gq4[17,], gq1[18,], gq2[18,], gq3[18,], gq4[18,], gq1[19,], gq2[19,], gq3[19,], gq4[19,], gq1[20,], gq2[20,], gq3[20,], gq4[20,], gq1[21,], gq2[21,], gq3[21,], gq4[21,], gq1[22,], gq2[22,], gq3[22,], gq4[22,], gq1[23,], gq2[23,], gq3[23,], gq4[23,], gq1[24,], gq2[24,], gq3[24,], gq4[24,], gq1[25,], gq2[25,], gq3[25,], gq4[25,], gq1[26,], gq2[26,], gq3[26,], gq4[26,], gq1[27,], gq2[27,], gq3[27,], gq4[27,], gq1[28,], gq2[28,], gq3[28,], gq4[28,] )

View(gq)

write.table(gq,"table2_gq_4g.txt")

################################################

######################################################################################################

#plots

#stratify by maf

d_pow<-data.frame(ideal=0,standard=0,new=0)

d_pow<-rbind(d_pow,

c(maf1[22,4],maf1[19,4],maf1[17,4]),

c(maf2[22,4],maf2[19,4],maf2[17,4]),

c(maf3[22,4],maf3[19,4],maf3[17,4]),

c(maf4[22,4],maf4[19,4],maf4[17,4]))

d_pow<-d_pow[-1,]

d_t1er<-data.frame(ideal=0,standard=0,new=0)

d_t1er<-rbind(d_t1er,

c(maf1[22,8],maf1[19,8],maf1[17,8]),

c(maf2[22,8],maf2[19,8],maf2[17,8]),

c(maf3[22,8],maf3[19,8],maf3[17,8]),

c(maf4[22,8],maf4[19,8],maf4[17,8]))

d_t1er<-d_t1er[-1,]

row.names(d_pow)<-c("\u2264 0.05","(0.05,0.10]","(0.10,0.20]",">0.20")

row.names(d_t1er)<-c("\u2264 0.05","(0.05,0.10]","(0.10,0.20]",">0.20")

png("power_maf_dis_paper_4g_2018.png")

barCenters_a <- barplot(height = t(as.matrix(d_pow)),beside = TRUE, ylim = c(0, 1), cex.axis=1.5,cex.names = 1,

,ylab = "Statistical Power",xlab = "Minor Allele Frequency",border = NA,axes = TRUE,col="transparent",axisnames=T,cex.lab=1.5)

rect(4.5,-1, 8.5,1, col = "gray90",border="transparent") # coloured

rect(12.5,-1, 16.5,1, col = "gray90",border="transparent") # coloured

for(i in seq(0,0.9,0.1)){

abline(h=i,lty=2,col="azure3")

}

legend("topleft", legend=c("Ideal Situation", "Called Genotype","Allele Counts"),col=c("hotpink1","dodgerblue1","limegreen"), pch=16,bg='white', box.lty = 0)

box()

n<-c(length(maf1_a[,1]),length(maf2_a[,1]),length(maf3_a[,1]),length(maf4_a[,1]))

n_pow<-rbind(n,n,n)

p<-t(d_pow)

q<-1-p

ci.up<-p+1.96*sqrt(p*q/n_pow)

ci.low<-p-1.96*sqrt(p*q/n_pow)

segments(barCenters_a[1,1], ci.up[1,1], barCenters_a[1,1],ci.low[1,1], lwd = 2,col="hotpink1")

segments(barCenters_a[2,1], ci.up[2,1], barCenters_a[2,1],ci.low[2,1], lwd = 2,col="dodgerblue1")

segments(barCenters_a[3,1], ci.up[3,1], barCenters_a[3,1],ci.low[3,1], lwd = 2,col="limegreen")

segments(barCenters_a[1,2], ci.up[1,2], barCenters_a[1,2],ci.low[1,2], lwd = 2,col="hotpink1")

segments(barCenters_a[2,2], ci.up[2,2], barCenters_a[2,2],ci.low[2,2], lwd = 2,col="dodgerblue1")

segments(barCenters_a[3,2], ci.up[3,2], barCenters_a[3,2],ci.low[3,2], lwd = 2,col="limegreen")

segments(barCenters_a[1,3], ci.up[1,3], barCenters_a[1,3],ci.low[1,3], lwd = 2,col="hotpink1")

segments(barCenters_a[2,3], ci.up[2,3], barCenters_a[2,3],ci.low[2,3], lwd = 2,col="dodgerblue1")

segments(barCenters_a[3,3], ci.up[3,3], barCenters_a[3,3],ci.low[3,3], lwd = 2,col="limegreen")

segments(barCenters_a[1,4], ci.up[1,4], barCenters_a[1,4],ci.low[1,4], lwd = 2,col="hotpink1")

segments(barCenters_a[2,4], ci.up[2,4], barCenters_a[2,4],ci.low[2,4], lwd = 2,col="dodgerblue1")

segments(barCenters_a[3,4], ci.up[3,4], barCenters_a[3,4],ci.low[3,4], lwd = 2,col="limegreen")

arrows(barCenters_a[1,1], ci.low[1,1], barCenters_a[1,1],ci.up[1,1], lwd =2, angle = 90, code = 3, length = 0.05,col="hotpink1")

arrows(barCenters_a[2,1], ci.low[2,1], barCenters_a[2,1],ci.up[2,1], lwd =2, angle = 90, code = 3, length = 0.05,col="dodgerblue1")

arrows(barCenters_a[3,1], ci.low[3,1], barCenters_a[3,1],ci.up[3,1], lwd =2, angle = 90, code = 3, length = 0.05,col="limegreen")

arrows(barCenters_a[1,2], ci.low[1,2], barCenters_a[1,2],ci.up[1,2], lwd =2, angle = 90, code = 3, length = 0.05,col="hotpink1")

arrows(barCenters_a[2,2], ci.low[2,2], barCenters_a[2,2],ci.up[2,2], lwd =2, angle = 90, code = 3, length = 0.05,col="dodgerblue1")

arrows(barCenters_a[3,2], ci.low[3,2], barCenters_a[3,2],ci.up[3,2], lwd =2, angle = 90, code = 3, length = 0.05,col="limegreen")

arrows(barCenters_a[1,3], ci.low[1,3], barCenters_a[1,3],ci.up[1,3], lwd =2, angle = 90, code = 3, length = 0.05,col="hotpink1")

arrows(barCenters_a[2,3], ci.low[2,3], barCenters_a[2,3],ci.up[2,3], lwd =2, angle = 90, code = 3, length = 0.05,col="dodgerblue1")

arrows(barCenters_a[3,3], ci.low[3,3], barCenters_a[3,3],ci.up[3,3], lwd =2, angle = 90, code = 3, length = 0.05,col="limegreen")

arrows(barCenters_a[1,4], ci.low[1,4], barCenters_a[1,4],ci.up[1,4], lwd =2, angle = 90, code = 3, length = 0.05,col="hotpink1")

arrows(barCenters_a[2,4], ci.low[2,4], barCenters_a[2,4],ci.up[2,4], lwd =2, angle = 90, code = 3, length = 0.05,col="dodgerblue1")

arrows(barCenters_a[3,4], ci.low[3,4], barCenters_a[3,4],ci.up[3,4], lwd =2, angle = 90, code = 3, length = 0.05,col="limegreen")

points(barCenters_a[1,1], d_pow[1,1] , pch = 20,col="hotpink1")

points(barCenters_a[2,1], d_pow[1,2] , pch = 20,col="dodgerblue1")

points(barCenters_a[3,1], d_pow[1,3] , pch = 20,col="limegreen")

points(barCenters_a[1,2], d_pow[2,1] , pch = 20,col="hotpink1")

points(barCenters_a[2,2], d_pow[2,2] , pch = 20,col="dodgerblue1")

points(barCenters_a[3,2], d_pow[2,3] , pch = 20,col="limegreen")

points(barCenters_a[1,3], d_pow[3,1] , pch = 20,col="hotpink1")

points(barCenters_a[2,3], d_pow[3,2] , pch = 20,col="dodgerblue1")

points(barCenters_a[3,3], d_pow[3,3] , pch = 20,col="limegreen")

points(barCenters_a[1,4], d_pow[4,1] , pch = 20,col="hotpink1")

points(barCenters_a[2,4], d_pow[4,2] , pch = 20,col="dodgerblue1")

points(barCenters_a[3,4], d_pow[4,3] , pch = 20,col="limegreen")

dev.off()

##t1er

png("t1er_maf_dis_paper_4g_2018.png")

barCenters_a <- barplot(height = t(as.matrix(d_t1er)),beside = TRUE, ylim = c(-0.01, 0.25), cex.axis=1.5,cex.names = 1,

,ylab = "Type I Error Rate",xlab = "Minor Allele Frequency",border = NA,axes = TRUE,col="transparent",axisnames=T,cex.lab=1.5)

rect(4.5,-1, 8.5,1, col = "gray90",border="transparent") # coloured

rect(12.5,-1, 16.5,1, col = "gray90",border="transparent") # coloured

legend("topleft", legend=c("Ideal Situation", "Called Genotype","Allele Counts"),col=c("hotpink1","dodgerblue1","limegreen"), pch=16,bg='white', box.lty = 0)

box()

n<-c(length(maf1_n[,1]),length(maf2_n[,1]),length(maf3_n[,1]),length(maf4_n[,1]))

n_t1er<-rbind(n,n,n)

p<-t(d_t1er)

q<-1-p

ci.up<-p+1.96*sqrt(p*q/n_t1er)

ci.low<-p-1.96*sqrt(p*q/n_t1er)

abline(h=0.05,lty=2,col="azure4")

segments(barCenters_a[1,1], ci.up[1,1], barCenters_a[1,1],ci.low[1,1], lwd = 2,col="hotpink1")

segments(barCenters_a[2,1], ci.up[2,1], barCenters_a[2,1],ci.low[2,1], lwd = 2,col="dodgerblue1")

segments(barCenters_a[3,1], ci.up[3,1], barCenters_a[3,1],ci.low[3,1], lwd = 2,col="limegreen")

segments(barCenters_a[1,2], ci.up[1,2], barCenters_a[1,2],ci.low[1,2], lwd = 2,col="hotpink1")

segments(barCenters_a[2,2], ci.up[2,2], barCenters_a[2,2],ci.low[2,2], lwd = 2,col="dodgerblue1")

segments(barCenters_a[3,2], ci.up[3,2], barCenters_a[3,2],ci.low[3,2], lwd = 2,col="limegreen")

segments(barCenters_a[1,3], ci.up[1,3], barCenters_a[1,3],ci.low[1,3], lwd = 2,col="hotpink1")

segments(barCenters_a[2,3], ci.up[2,3], barCenters_a[2,3],ci.low[2,3], lwd = 2,col="dodgerblue1")

segments(barCenters_a[3,3], ci.up[3,3], barCenters_a[3,3],ci.low[3,3], lwd = 2,col="limegreen")

segments(barCenters_a[1,4], ci.up[1,4], barCenters_a[1,4],ci.low[1,4], lwd = 2,col="hotpink1")

segments(barCenters_a[2,4], ci.up[2,4], barCenters_a[2,4],ci.low[2,4], lwd = 2,col="dodgerblue1")

segments(barCenters_a[3,4], ci.up[3,4], barCenters_a[3,4],ci.low[3,4], lwd = 2,col="limegreen")

arrows(barCenters_a[1,1], ci.low[1,1], barCenters_a[1,1],ci.up[1,1], lwd =2, angle = 90, code = 3, length = 0.05,col="hotpink1")

arrows(barCenters_a[2,1], ci.low[2,1], barCenters_a[2,1],ci.up[2,1], lwd =2, angle = 90, code = 3, length = 0.05,col="dodgerblue1")

arrows(barCenters_a[3,1], ci.low[3,1], barCenters_a[3,1],ci.up[3,1], lwd =2, angle = 90, code = 3, length = 0.05,col="limegreen")

arrows(barCenters_a[1,2], ci.low[1,2], barCenters_a[1,2],ci.up[1,2], lwd =2, angle = 90, code = 3, length = 0.05,col="hotpink1")

arrows(barCenters_a[2,2], ci.low[2,2], barCenters_a[2,2],ci.up[2,2], lwd =2, angle = 90, code = 3, length = 0.05,col="dodgerblue1")

arrows(barCenters_a[3,2], ci.low[3,2], barCenters_a[3,2],ci.up[3,2], lwd =2, angle = 90, code = 3, length = 0.05,col="limegreen")

arrows(barCenters_a[1,3], ci.low[1,3], barCenters_a[1,3],ci.up[1,3], lwd =2, angle = 90, code = 3, length = 0.05,col="hotpink1")

arrows(barCenters_a[2,3], ci.low[2,3], barCenters_a[2,3],ci.up[2,3], lwd =2, angle = 90, code = 3, length = 0.05,col="dodgerblue1")

arrows(barCenters_a[3,3], ci.low[3,3], barCenters_a[3,3],ci.up[3,3], lwd =2, angle = 90, code = 3, length = 0.05,col="limegreen")

arrows(barCenters_a[1,4], ci.low[1,4], barCenters_a[1,4],ci.up[1,4], lwd =2, angle = 90, code = 3, length = 0.05,col="hotpink1")

arrows(barCenters_a[2,4], ci.low[2,4], barCenters_a[2,4],ci.up[2,4], lwd =2, angle = 90, code = 3, length = 0.05,col="dodgerblue1")

arrows(barCenters_a[3,4], ci.low[3,4], barCenters_a[3,4],ci.up[3,4], lwd =2, angle = 90, code = 3, length = 0.05,col="limegreen")

points(barCenters_a[1,1], d_t1er[1,1] , pch = 20,col="hotpink1")

points(barCenters_a[2,1], d_t1er[1,2] , pch = 20,col="dodgerblue1")

points(barCenters_a[3,1], d_t1er[1,3] , pch = 20,col="limegreen")

points(barCenters_a[1,2], d_t1er[2,1] , pch = 20,col="hotpink1")

points(barCenters_a[2,2], d_t1er[2,2] , pch = 20,col="dodgerblue1")

points(barCenters_a[3,2], d_t1er[2,3] , pch = 20,col="limegreen")

points(barCenters_a[1,3], d_t1er[3,1] , pch = 20,col="hotpink1")

points(barCenters_a[2,3], d_t1er[3,2] , pch = 20,col="dodgerblue1")

points(barCenters_a[3,3], d_t1er[3,3] , pch = 20,col="limegreen")

points(barCenters_a[1,4], d_t1er[4,1] , pch = 20,col="hotpink1")

points(barCenters_a[2,4], d_t1er[4,2] , pch = 20,col="dodgerblue1")

points(barCenters_a[3,4], d_t1er[4,3] , pch = 20,col="limegreen")

dev.off()

#mannhattan

results3<-read.table("results3.txt",header=T)

gt_a_na3<-read.table("gt_a_na3.txt",header=T,stringsAsFactors=FALSE)

library(HardyWeinberg)

library(LDheatmap)

library(genetics)

library(ggplot2)

apply(gt_a_na3[,-c(1,2)],1,table())/apply(apply(gt_a_na3[,-c(1,2)],1,table()),1,sum())

maf<-data.frame(pos=0,AA=0,BA=0,BB=0,maf=0)

for(i in 1:length(gt_a_na3$CHROM)){

maf[i,]<-c(gt_a_na3[i,]$POS,table(as.character(gt_a_na3[i,-c(1,2)]))["A/A"],table(as.character(gt_a_na3[i,-c(1,2)]))["B/A"],table(as.character(gt_a_na3[i,-c(1,2)]))["B/B"],0)

if(is.na(maf[i,2])){maf[i,2]<-0}

if(is.na(maf[i,3])){maf[i,3]<-0}

if(is.na(maf[i,4])){maf[i,4]<-0}

maf[i,5]<-maf(c(maf[i,2],maf[i,3],maf[i,4])/sum(maf[i,2:4]))

}

rownames(gt_a_na3)<-gt_a_na3$POS

pos_maf<-subset(maf,maf>=0.20)$pos

gt_a_na_maf<-gt_a_na3[as.character(pos_maf),]

posn<-gt_a_na_maf$POS

rownames(gt_a_na_maf)<-NULL

########################################################################################################

#######################################################################################################

# Dprime only in region of interest LDplot

gt_a_na_maf1<-subset(gt_a_na_maf,gt_a_na_maf$POS>=34186492)

gt_a_na_maf2<-subset(gt_a_na_maf1,gt_a_na_maf1$POS<=34269343)

test<-t(gt_a_na_maf2)

colnames(test)<-test[2,]

test<-test[-c(1,2),]

test<-as.data.frame(test)

for(i in 1:length(gt_a_na_maf2[,1])){

test[,i]<-as.genotype(test[,i])

}

testDist<-as.vector(as.numeric(colnames(test)))

MyLDheatmap_D<-LDheatmap(test,testDist,LDmeasure = "D'", add.map = TRUE, name = "myLDgrob", add.key = TRUE, flip=T,geneMapLocation =0.25,color=heat.colors(20),newpage=FALSE)

#LDheatmap.highlight(MyLDheatmap_D, i = 23, j = 43, col = "black", fill = "grey" )

LDheatmap.marks(MyLDheatmap_D, 72, gp=gpar(cex=2), pch = "*")

LDheatmap.marks(MyLDheatmap_D, 65, gp=gpar(cex=2), pch = "(")

LDheatmap.marks(MyLDheatmap_D, 92, gp=gpar(cex=2), pch = ")")

#old plot region

results3_1<-subset(results3,results3$pos>=34186492)

results3_2<-subset(results3_1,results3_1$pos<=34269343)

region1<-subset(results3_2,results3_2$pos>=34235868)

region<-subset(region1,region1$pos<=34258692)

png("manhattan92CEU_101YRI_0pca_region_col_peak_paper.png")

#plot(results3_2$pos[-1],-log10(results3_2$ratio_phd_lm[-1]),col="#2c7fb8",pch=20,xlab="Position",ylab="-log(pvalue)",ylim=c(0,10))

#points(results3_2$pos[-1],-log10(results3_2$gt_phd_ordinal_logit [-1]),col="gray",pch=20)

#lines(rep(34236000,2),c(0,120),lty=1,lwd=2,col="red")

#lines(rep(34236311,2),c(0,120),lty=1,lwd=2,col="blue")

#lines(rep(34235961,2),c(0,120),lty=1,lwd=2,col="blue")

#lines(rep(34235868,2),c(0,120),lty=1,lwd=2)

#lines(rep(34258692,2),c(0,120),lty=1,lwd=2)

plot(results3_2$pos,-log10(results3_2$gt_phd_ordinal_logit),col="lightskyblue",pch=20,xlab="Position",ylab="-log(pvalue)",ylim=c(0,55),cex.lab=1.5,cex.axis=1.5)

points(results3_2$pos,-log10(results3_2$ratio_phd_lm),col="darkseagreen1",pch=20)

points(region$pos,-log10(region$gt_phd_ordinal_logit),col="dodgerblue1",pch=20)

points(region$pos,-log10(region$ratio_phd_lm),col="limegreen",pch=20)

legend("topright", legend=c("Called Genotype","Allele Counts"),col=c("dodgerblue1","limegreen"), pch=16,bg='white', box.lty = 0)

box()

dev.off()
